# Mild traumatic brain injury increases cortical iron: evidence from individual susceptibility mapping

**DOI:** 10.1093/braincomms/fcaf110

**Published:** 2025-03-12

**Authors:** Christi A Essex, Devon K Overson, Jenna L Merenstein, Trong-Kha Truong, David J Madden, Mayan J Bedggood, Catherine Morgan, Helen C Murray, Samantha J Holdsworth, Ashley W Stewart, Richard L M Faull, Patria Hume, Alice Theadom, Mangor Pedersen

**Affiliations:** Department of Psychology and Neuroscience, Auckland University of Technology, Auckland 0627, New Zealand; Brain Imaging and Analysis Center, Duke University Medical Center, Durham, NC 27710, USA; Brain Imaging and Analysis Center, Duke University Medical Center, Durham, NC 27710, USA; Brain Imaging and Analysis Center, Duke University Medical Center, Durham, NC 27710, USA; Brain Imaging and Analysis Center, Duke University Medical Center, Durham, NC 27710, USA; Department of Psychology and Neuroscience, Auckland University of Technology, Auckland 0627, New Zealand; Center for Advanced MRI, The University of Auckland, Auckland 1023, New Zealand; School of Psychology, The University of Auckland, Auckland 1142, New Zealand; Center for Brain Research, The University of Auckland, Auckland 1023, New Zealand; Center for Brain Research, The University of Auckland, Auckland 1023, New Zealand; Center for Brain Research, The University of Auckland, Auckland 1023, New Zealand; Mātai Medical Research Institute, Gisborne 4010, New Zealand; Faculty of Medical and Health Sciences, The University of Auckland, Auckland 1023, New Zealand; Center for Advanced Imaging, The University of Queensland, Queensland 4067, Australia; Center for Brain Research, The University of Auckland, Auckland 1023, New Zealand; School of Sport and Recreation, Faculty of Health and Environmental Science, Sports Performance Research Institute New Zealand, Auckland University of Technology, Auckland 0627, New Zealand; Department of Psychology and Neuroscience, Auckland University of Technology, Auckland 0627, New Zealand; Department of Psychology and Neuroscience, Auckland University of Technology, Auckland 0627, New Zealand

**Keywords:** brain iron, cerebral cortex, individualized profiles, mild traumatic brain injury, quantitative susceptibility mapping

## Abstract

Quantitative susceptibility mapping has been applied to map brain iron distribution after mild traumatic brain injury to understand properties of neural tissue which may be related to cellular dyshomeostasis. However, this is a heterogeneous injury associated with microstructural brain changes, and ‘traditional’ group-wise statistical approaches may lead to a loss of clinically relevant information, as subtle alterations at the individual level can be obscured by averages and confounded by within-group variability. More precise and individualized approaches are needed to characterize mild traumatic brain injury better and elucidate potential cellular mechanisms to improve intervention and rehabilitation. To address this issue, we use quantitative MRI to build individualized profiles of regional positive (iron-related) magnetic susceptibility across 34 bilateral cortical ROIs following mild traumatic brain injury. Healthy population templates were constructed for each cortical area using standardized *Z*-scores derived from 25 age-matched male controls aged between 16 and 32 years (*M* = 21.10, SD = 4.35), serving as a reference against which *Z*-scores of 35 males with acute (<14 days) sports-related mild traumatic brain injury were compared [*M* = 21.60 years (range: 16–33), SD = 4.98]. Secondary analyses sensitive to cortical depth and curvature were also generated to approximate the location of iron accumulation in the cortical laminae and the effect of gyrification. Primary analyses indicated that approximately one-third (11/35; 31%) of injured participants exhibited elevated positive susceptibility indicative of abnormal iron profiles relative to the healthy population, a finding that was mainly concentrated in regions within the temporal lobe. Injury severity was significantly higher (*P* = 0.02) for these participants than their iron-normal counterparts, suggesting a link between injury severity, symptom burden, and elevated cortical iron. Secondary exploratory analyses of cortical depth and curvature profiles revealed abnormal iron accumulation in 83% (29/35) of mild traumatic brain injury participants, enabling better localization of injury-related changes in iron content to specific loci within each region and identifying effects that may be more subtle and lost in region-wise averaging. Our findings suggest that individualized approaches can further elucidate the clinical relevance of iron in mild head injury. Differences in injury severity between iron-normal and iron-abnormal mild traumatic brain injury participants identified in our primary analysis highlight not only why precise investigation is required to understand the link between objective changes in the brain and subjective symptomatology, but also identify iron as a candidate biomarker for tissue pathology after mild traumatic brain injury.

## Teaser

Essex *et al*. report that individualized quantitative susceptibility mapping reveals depth- and curvature-specific cortical iron abnormalities following mild traumatic brain injury. Elevated iron was evident primarily in the temporal lobe, and regional iron accumulation was associated with greater injury severity. These findings implicate cortical iron dysregulation in post-injury pathophysiology.

## Graphical Abstract

**Graphical Abstract:**
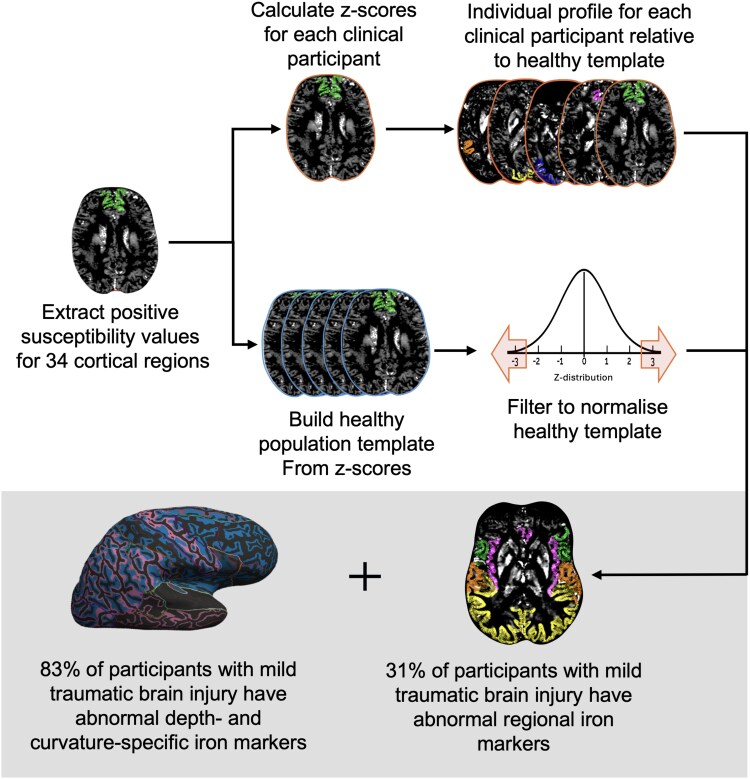

## Introduction

Exposure to mild traumatic brain injury (mTBI) is a significant public and personal health concern, accounting for ∼90% of the 50–60 million annual cases of traumatic brain injury (TBI) worldwide.^1^ Global financial losses related to TBI are estimated at ∼USD $400 billion per year,^1,2^ however, beyond the economic impacts mTBI can increase the risk of neurodegeneration, dementia^3,4^ and premature death.^5^ In the short term, mTBI can result in a range of symptoms with significant inter-individual variability, including cognitive, emotional and physiological disturbances such as sleep disruption, light sensitivity, fatigue, headaches, vertigo, vestibular problems, depression and anxiety, which significantly impact quality of life and participation in day-to-day activities for many.^4^ In some cases, these symptoms can persist up to three decades post-injury.^6,7^ Numerous factors contribute to differences in injury severity, symptom burden, *in vivo* brain tissue pathology and even autopsy findings. These include individual differences prior to injury such as genetic predispositions, age, gender, IQ, psychiatric history, prior mTBI exposure and substance use history, as well as differences in the mechanisms and loci of injury.^8^ In sports-related mTBI (sr-mTBI), for example, variability in the sport and even player position can affect injury severity, lead to diverse effects on brain structure and function, and divergence in symptom burden and cluster.^8^

The heterogeneity of mTBI is apparent at even the cellular level. The rapid changes in inertia (acceleration/deceleration/rotation) or exogenous skull impact associated with mTBI cause the transmission of mechanical forces to the brain, resulting in a mechanistically specific primary insult and microstructural tissue damage.^8,9^ This initiates a variable cascade of secondary cellular processes, including disruption of the blood–brain barrier (BBB), cerebrovascular dysfunction, oxidative stress, axonal degeneration and neuroinflammation^9,10^ which can propagate for months after the initial impact.^11^ However, the pathophysiology of mTBI remains poorly understood, and specific biomarkers indicative of mTBI remain, to date, elusive. Unlike moderate-to-severe TBI (ms-TBI), where lesions, haemorrhages or macroscopic morphological abnormalities can be detected, routine MRI methods are often insensitive to mTBI-related neuropathology.^12,13^ This limitation necessitates the use of advanced MRI techniques not typically employed in conventional medical settings to identify the subtle changes in brain structure characteristic of this ‘mild’ injury.^14,15^ Integrating these advanced imaging modalities into routine patient care requires further validation and the establishment of clinically and individually relevant biomarkers for mTBI diagnosis and treatment.

Iron accumulation is increasingly recognized as a component of neuropathology following mTBI, contributing not only to acute-phase secondary injury and later cell death,^9^ but also cognitive dysfunction after mTBI.^16,17^ Quantitative susceptibility mapping (QSM) is an advanced MRI technique that can be used to estimate the magnetic susceptibility of tissue, such as paramagnetism exhibited by iron in response to an applied magnetic field.^18-23^ Non-heme iron (particularly ferritin-bound iron), is the main source of paramagnetism on QSM^18,24-26^ and widely recognized as the form of iron most involved in secondary injury after mTBI.^9,27,28^ Iron dyshomeostasis can trigger auto-toxic circuits that drive neurodegenerative processes,^29^ including the generation of reactive oxygen species, which at high levels can lead to cytotoxic oxidative stress,^30^ lipid damage and increased permeability of the cell membrane,^9^ as well as iron-regulated cell death (ferroptosis).^31^ As such, elevated levels of iron in cortical regions would suggest localization of injury-related pathological processes and changes in brain structure. These changes may be related, but not limited to, mTBI-induced permeability of the BBB^32^ and neuroinflammation,^33^ both of which are known to be involved in iron accumulation.^9,30^ Iron has also been implicated in the hyperphosphorylation of tau proteins (p-tau)^28^ observed in mTBI-related tauopathies; its co-localization with p-tau thus identifies it as a promising early indicator of neurodegeneration.^34,35^

A limited number of studies have employed QSM to investigate the role of brain iron in microstructural tissue damage following mTBI, focusing mainly on subcortical nuclei or global grey and/or white matter (WM),^36-44^ with only a few studies including cortical regions of interest (ROIs)^38,41^ or investigating the relevance of cortical morphology.^45^ However, the diversity of mTBI effects may not be discernible at the group level, which currently constitutes the standard statistical approach. Individual-level investigations of injury-specific effects may better characterize mTBI-related neuropathology, and are increasingly recognized as providing more biologically informative data than group-level studies, especially in clinical populations where targeted interventions are both useful and necessary,^46^ such as mTBI. Personalized profiles can be generated by leveraging *Z*-scores to compare the results of individual quantitative measures to the distribution of a healthy normative population. This approach allows for a clearer understanding of where the individual falls relative to normal ranges for selected metrics.^47^ Individual analytic approaches have been successfully applied in the context of mTBI using T2 relaxometry as a marker of neuroinflammation,^48^ and diffusion-weighted imaging (DWI) to investigate WM fibre tracts.^49^ Under the TBI umbrella more broadly, individual analyses have been applied to fixel-based analysis of diffusion MRI^50^ and diffusion tensor imaging (DTI)^51^ to investigate WM integrity, as well as structural connectomics,^52^ in ms-TBI. One study has used QSM to generate individualized profiles of iron deposition in ms-TBI,^47^ however, dedicated personalized investigations of iron deposition at the individual level following *mild* TBI are, to the authors knowledge, lacking.

To address these research gaps, we conducted the first dedicated individual-level investigation of iron-related mTBI effects. This study aimed to: (i) generate individual profiles of cortical iron deposition following sr-mTBI, and; (ii) extend these findings by deriving profiles sensitive to cortical architectonics (depth and curvature) as a supplemental, secondary approach. Our prior research suggests that elevated magnetic susceptibility should be most evident in ROIs in the temporal lobe.^45^ However, given the preliminary and exploratory nature of this study, we did not have specific *a priori* hypotheses about the direction of effects in all cortical regions.

## Materials and methods

Ethical approval for this research was obtained from the Health and Disabilities Ethics Committee (HDEC) (Date: 18/02/2022, Reference: 2022 EXP 11078) and institutional approval was also obtained from the Auckland University of Technology Ethics Committee (AUTEC) (Date: 18/02/2022, Reference: 22/12). In accordance with the Declaration of Helsinki, all participants provided written informed consent prior to data collection.

### Participants

Thirty-five male contact sports players [*M* = 21.60 years (range: 16–33), SD = 4.98] with acute sr-mTBI [sustained within 14 days of MRI scanning (*M* = 10.40 days, SD = 3.01)] and 25 age-matched male controls [*M* = 21.10 years (range: 16–32), SD = 4.35] were recruited for this observational study (see Table 1). To mitigate potential age-related confounds, we ensured that ages were not significantly different between groups [*t*(58) = −0.44, *P* = 0.66]. Clinical (sr-mTBI) participants were recruited through three Axis Sports Medicine Clinics (Auckland, New Zealand), via print and social media advertisements, word-of-mouth and through community-based referrals from healthcare professionals and sports team management. Each clinical participant was required to have a confirmed sr-mTBI diagnosis by a licensed physician as a prerequisite for study inclusion, and symptom severity was assessed using the Brain Injury Screening Tool (BIST)^53^ either upon presentation to Axis Clinics or electronically following recruitment. Healthy controls (HC) were recruited through print and social media advertisements, and word-of-mouth. Exclusion criteria for all the participants included a history of significant medical or neurological conditions unrelated to the study’s objectives and contraindications for MRI. Additionally, controls were excluded if they had any recent history of mTBI events (<12 months) or were living with any long-term effects of previous mTBI. All participants completed a brief demographic questionnaire and attended a 1-h MRI scan at The Centre for Advanced MRI (CAMRI), Auckland, New Zealand. All scans were reviewed by a certified neuroradiologist consultant for clinically significant findings. No findings from MRI in either group were considered clinically significant (see Table 1).

**Table 1:** Summary of sr-mTBI participant clinical characteristics

ID	Age	DSI	BIST	MOI	MRI findings
mTBI-01	<20	5	140	Rugby	None
mTBI-02	<20	5	12	Rugby	None
mTBI-03	20s	6	78	Rugby	None
mTBI-04	<20	13	18	Rugby	Small fluid signal spaces in R peritrigonal WM—normal. R caudate cleft along ventricular surface—possibly developmental or from old ischaemic insult
mTBI-05	20s	13	42	Football	None
mTBI-06	20s	13	13	Hockey	Minor artifactual T1 signal in pons
mTBI-07	20s	12	6	Rugby	None
mTBI-08	20s	6	56	Rugby	Minor R orbital fracture (old)
mTBI-09	<20	12	54	Rugby	None
mTBI-10	20s	10	52	Rugby	None
mTBI-11	30s	13	13	Football	None
mTBI-12	<20	5	79	Rugby	None
mTBI-13	20s	13	2	Rugby	Small focus of susceptibility in L superior frontal gyrus possibly vascular or nonspecific haemosiderin
mTBI-14	<20	13	22	Rugby	None
mTBI-15	<20	8	117	Futsal	Tiny cleft of fluid signal in R cingulate gyrus—minor developmental anomaly or mature gliosis
mTBI-16	20s	13	^a^	Rugby	None
mTBI-17	20s	10	34	Gymnastics	None
mTBI-18	20s	13	28	Jiu-jitsu	Some artifactual DWI signal in pons
mTBI-19	20s	11	69	Surfing	Tiny susceptibility site in R temporal lobe—may be vascular
mTBI-20	<20	7	14	Rugby	Minor susceptibility in transverse sulcus in R mid temporal lobe—nonspecific, may be vascular or reflect haemosiderin deposition from prior small volume haemorrhage
mTBI-21	20s	14	47	Rugby	None
mTBI-22	<20	12	28	Football	None
mTBI-23	<20	13	39	Judo	None
mTBI-24	<20	9	34	Rugby	None
mTBI-25	<20	12	68	Rugby	None
mTBI-26	20s	12	17	Rugby	7 mm pineal cyst—normal limits. Some T1 hyperintensity in R cerebellum—artifact compatible
mTBI-27	<20	12	12	Rugby	None
mTBI-28	20s	12	25	Rugby	Mildly prominent cisterna magna
mTBI-29	30s	7	30	Football	A few mildly prominent biparietal and L cerebral peduncle perivascular spaces—normal variant
mTBI-30	30s	12	51	Swimming	None
mTBI-31	20s	5	6	Rugby	None
mTBI-32	<20	12	2	Rugby	Some DWI signal disturbance anterior to pons—likely artifactual
mTBI-33	<20	14	22	Rugby	2–3 tiny foci of susceptibility in R frontal lobe—nonspecific, possible site of prior microhaemorrhage. A punctate focus of T1 hypointensity/T2 hyperintensity superolateral to the frontal horn of R lateral ventricle
mTBI-34	20s	8	58	Football	Bifrontal developmental venous anomaly noted—normal variants
mTBI-35	<20	8	8	Rugby	Minuscule foci of susceptibility in R cerebellar hemisphere/posterior to R aspect of the splenium of CC—non-specific. Minor susceptibility in R sylvian fissure—vascular
Mean mTBI	21.60 (4.98) years of age	10.4 (3.01) DSI	38.1 (32.0)/160 BIST		No findings considered clinically relevant
Mean HC	21.10 (4.35) years of age				No findings considered clinically relevant

Diagnostic assessment is limited to the volume T1, SWI and DWI sequences with only limited interpretation of the multi-echo T2 stack. Clinical assessments are relevant to the identification of micro-haemorrhages, areas of siderosis, T1 appearance, gliosis, volume, ventricular volumes and non-neurological findings. The possible range of BIST scores is 0 (min)–160 (max). Clinical group data correspond to the date of MRI only, except for BIST scores acquired >24 h post-injury and prior to MRI (<14 days post). ID, unique identifier; DSI, days since injury; BIST, Brain Injury Screening Tool; MOI, mechanism of injury; WM, white matter; CC, corpus callosum; DWI, diffusion-weighted imaging; L, left; R, right. ^a^Missing data (BIST incomplete on the Axis Sport Medicine Clinic patient portal, reason unknown).

### Neuroimaging

Details on image acquisition and processing have been previously reported,^45^ and are summarized here for brevity.

#### Acquisition

MRI data were acquired on a 3T Siemens MAGNETOM Vida Fit scanner (Siemens Healthcare, Erlangen, Germany) equipped with a 20-channel head coil. A 3D flow-compensated Gradient Echo sequence was used to obtain magnitude and unfiltered phase images for QSM reconstruction. Data were collected at 1 mm isotropic voxel size with matrix size = 180 × 224 × 160 mm, Repetition Time (TR) = 30 ms; Echo Time (TE) = 20 ms; Flip Angle (FA) = 15°; Field of View (FoV) = 180 mm (Left-Right)×224 mm (Anterior-Posterior), in a total acquisition time of ∼3.43 min. For each participant, a high-resolution 3D T1-weighted (T1w) anatomical image volume was acquired for coregistration and parcellation using a Magnetization-Prepared Rapid Acquisition Gradient Echo (MPRAGE) sequence (TR = 1940.0 ms; TE = 2.49 ms, FA = 9°; slice thickness = 0.9 mm; FoV = 230 mm; matrix size = 192 × 512 × 512 mm; GRAPPA = 2; voxel size 0.45 × 0.45 × 0.90 mm) for a total acquisition time of ∼4.31 min. Digital Imaging and Communications in Medicine files were converted to Neuroimaging Informatics Technology Initiative (NIfTI) files and transformed to brain imaging data structure^54^ for further processing using *Dcm2Bids*^55^ version 3.1.1, which is a wrapper for *dcm2niix*^56^ (v1.0.20230411).

#### Image processing

Bias field-corrected^57,58^ T1w images were processed in FreeSurfer^59^ to: (i) delineate pial and grey matter/WM (GM/WM) boundary meshes and (ii) generate estimates of cortical thickness and curvature for each vertex.^60^ QSM images were reconstructed using a rapid open-source minimum spanning tree algorithm (ROMEO),^61^ background field removal with projection onto dipole fields^62^ and sparsity-based rapid two-step dipole inversion^63^; a pipeline congruent with recent consensus statement recommendations for best-practice QSM reconstruction.^64^ Whilst the optimal reference region for susceptibility estimation remains debated,^65^ recent consensus guidelines recommend quantifying QSM relative to a specific reference structure.^64^ To comply with these guidelines and ensure stability and reproducibility, QSM was referenced to whole-brain susceptibility as smaller regions can be more vulnerable to artefacts, distortions and inhomogeneities, affecting the final brain map.^64^ All QSM reconstruction was carried out via QSM×T^66^ v6.4.2 (https://qsmxt.github.io/QSMxT/) and used a robust two-pass combination method for artefact reduction.^67^

Subsequent processing was performed using the Functional Neuroimaging Research Group (FMRIB) Software Library.^68-70^ For each subject, the raw magnitude image was skull-stripped^71^ and binarised. These binary masks were used to erode non-brain signal around the brain perimeter using *fslmaths*. Magnitude images were linearly coregistered to the T1w image using FMRIB’s Linear Transformation Tool (*FLIRT*)^72-74^ with 12 degrees of freedom (DoF). Due to variability in acquisition type, field-of-view and matrix size between subjects’ QSM and T1w images, the 12 DoF linear registrations provided more accurate alignment compared to the six DoF alternative, allowing for better compensation of non-rigid anatomical variations upon visual inspection. The resulting transformation matrix was used for spatial normalization of the QSM images to T1w space. In line with prior research,^60^ QSM maps were thresholded into separate inter-voxel sign (positive and negative) maps with *fslmaths*. Traditional QSM maps represent average intra-voxel susceptibility values,^75^ which may obscure individual susceptibility sources. Further confounding effects may be introduced via inter-voxel averaging during analysis. This thresholding approach may help address the latter limitation by isolating voxels containing predominantly paramagnetic substrates, such as iron, which could enable more targeted analyses of susceptibility sources. Only positive sign maps were used in analyses to target cortical iron distribution.

#### Cortical column generation

To generate cortical columns and sample positive susceptibility values, we adapted a pipeline previously applied to DWI^76^ and QSM^45,60^ for depth- and curvature-specific cortical analysis. First, the T1w FreeSurfer^59^ recon served as an input into the *easy_lausanne* tool (https://github.com/mattcieslak/easy_lausanne.git), based on the open-source Connectome Mapper,^77^ to separate the cortex into 34 ROIs per hemisphere according to the Lausanne multi-scale atlas (equivalent to the Desikan-Killiany atlas^78^ native to FreeSurfer).^59^

##### Depth

Cortical columns were created for each hemisphere in T1w space with *write_mrtrix_tracks*^79^ in MATLAB (version R2024a), which was used to connect vertex pairs between pial and GM/WM boundary surface meshes. Each cortical column was segmented into six equidistant depths extending from the pial surface to the GM/WM boundary using MRtrix3 *tckresample*.^79^ This approach differs from previous studies using a 21-depth approach.^45,60,76^ Here, we used six depths to decrease the number of depth-wise comparisons, and to better approximate the structure of the intracortical layers.^80,81^ It should be noted that these depths represent equidistant segmentations rather than specific cellular laminae (layers I–VI) of the cortex, differing from ultra-high-field investigations of cortical cyto- and myelo-architecture. Results described here are related to cortical *depth*, rather than *layer*.

##### Curvature

The columns were also categorized based on cortical curvature, derived from FreeSurfer’s^59^ Gaussian curvature values at each GM/WM boundary vertex^82^ and quantified in units of 1/mm^2^. The categories included the gyral crown (curvature values: −0.6 to −0.1), sulcal bank (−0.1 to 0.1), sulcal fundus (0.1–0.6), or whole ROI (−0.6 to 0.6).^60^ Positive curvature values indicated sulci, while negative values indicated gyri, with higher values corresponding to deeper curvatures.^60^ Only columns ranging from 0.5 to 6 mm in length were included in the analysis to capture plausible cortical morphology.^83^ Depth was measured in percentage of cortical thickness rather than absolute metrics (millimetre) to mitigate variability between the participants.

### Personalized QSM profiles

**Figure 1:**
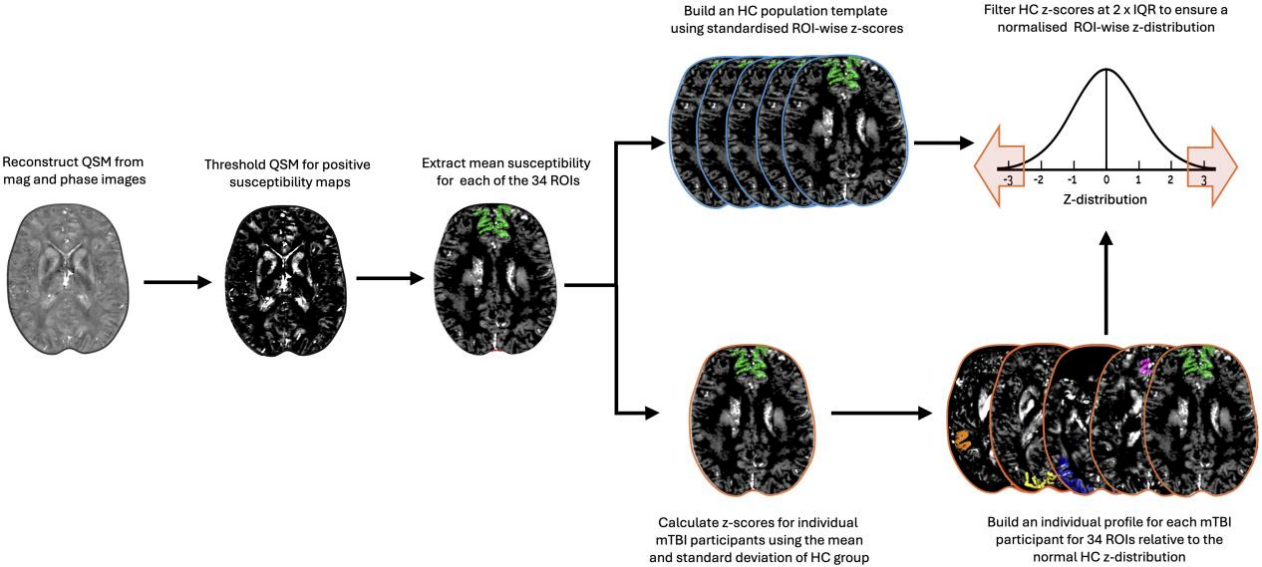
**QSM post-processing and generation of individual iron profiles.** Steps are performed after QSM image reconstruction using QSM×T. QSM images were thresholded to create a positive sign map, and mean susceptibility values were extracted for each ROI, as well as for each cortical depth (1–6) and curvature (crown, bank, and fundus). Z-scores were calculated using the mean and SD of the HC group, and standardized around a mean of zero. The HC distribution was then filtered to remove outliers exceeding two times IQR, normalizing the distribution. Individual profiles for mTBI participants were constructed by comparing each participant’s *Z*-scores to the healthy normal distribution, while controlling for multiple comparisons across the 34 cortical ROIs. Abbreviations are as follows: QSM, quantitative susceptibility mapping; ROI, region of interest; HC, healthy control; IQR, interquartile range.

We generated individual QSM profiles for each ROI at the bilateral level using MATLAB (2024a) (see Fig. 1 for visualization). Mean positive susceptibility values were extracted across the whole ROI (curvature and depth combined), as well as three curvature bins (gyral crown, sulcal bank and sulcal fundus) and six cortical depths independently for all 34 ROIs. For the whole-ROI profiles, *Z*-scores were calculated for all participants (HC and mTBI), by subtracting the HC group mean from each individual’s susceptibility value and dividing by the HC group SD; a method commonly used in prior research.^47,48,51,52^ To bring the HC data closer to a normal distribution, outlier scores for the HC group were filtered^47^ if they fell outside two times the interquartile range (IQR); a more stringent criterion than methods used to identify mild outliers at 1.5 times the IQR, but less extreme than the more conservative filter of three times the IQR.^84^ As a result, data from *N = 1* HC participants were excluded in three of the 34 ROIs, and data from *N = 2* HC participants were excluded in one of the 34 ROIs. After filtering, the Shapiro–Wilk normality test yielded an average *W*-value of *M* = 0.96 (SD = 0.02) across *Z*-distributions for all ROIs, indicating that the data distribution within each ROI was close to normal. The final equation for deriving the whole-ROI *Z*-scores for individual mTBI participants was as follows:


$$Z_{\mathrm{mTBI}}\;=\;\frac{X_{\mathrm{mTBI}}\;-\;\mu_{\mathrm{HCnorm}}}{\sigma HC_{\mathrm{norm}}},$$

where *Z*_mTBI_ represents the ROI-wise *Z*-score for each mTBI participant; *X*_mTBI_ is the ROI-wise mean QSM value for each mTBI participant; *µ*_HCnorm_ is the mean ROI-wise QSM value of the HC group after outlier filtering, and; *σ*_HCnorm_ is the ROI-wise SD of the HC group QSM values after filtering. This approach ensures that mTBI participants’ susceptibility values are directly comparable to the healthy range reflecting a normalized distribution. The same process was repeated for each depth at each curvature bin, how*e*ver, the IQR filter was omitted due to the number of comparison points.

### Statistical analysis

To assess statistical significance for whole-ROI mTBI *Z*-scores, two-tailed *P*-values were calculated from the *Z*-scores using the cumulative distribution function of the standard normal distribution. A false discovery rate (FDR) correction^85^ was applied to the *P*-values for 34 ROI-wise comparisons.

To conduct exploratory statistical tests, we divided the mTBI group into two subgroups: those whose *Z*-scores significantly deviated from HC norms (i.e. iron-abnormal) and those whose scores did not (i.e. iron-normal) as identified via primary and secondary analyses, respectively. Although there was no statistically significant difference in age between mTBI participants and controls, we performed an ANOVA for the new groupings to confirm that age was not driving the results. Additionally, we used nonparametric Mann–Whitney U-tests to assess whether injury severity (BIST^53^ scores) differed significantly between iron-abnormal and iron-normal mTBI participants, excluding mTBI-16 for these analyses only due to missing injury severity data.

## Results

### Regional individualized cortical iron profiles

We calculated personalized profiles of iron-related differences in positive susceptibility across 34 cortical ROIs for each mTBI participant, to understand the effects of mild brain trauma at the individual level. Of the 35 mTBI participants, 11 (31%) exhibited significantly elevated positive susceptibility for at least one ROI relative to the HC population template (see Table 2), likely indicating elevated iron. No clinical participants’ *Z*-scores were significantly lower than the HC population.

**Table 2:** Summary of affected ROI, *Z*-score, and symptomatology for iron-abnormal sr-mTBI participants

ID	Lobe(s)	ROI	*Z*-score	pFDR	Presenting symptoms
mTBI-01	Temporal: Occipital:	Bank, STS Lingual gyrus	+3.1 + 4.1	0.036 0.002	Initial emesis. Severe tinnitus and phonophobia, vertigo, cephalalgia, cervicalgia, photophobia, visual disturbances, cognitive impairment with confusion, concentration issues, memory deficits, irritability, restlessness, fatigue, sleep disturbances. Moderate vestibular dysfunction and ataxia noted.
mTBI-03	Temporal:	Parahippocampal	+3.7	0.008	Moderate cervicalgia, cephalalgia, photophobia, phonophobia, vertigo, vestibular dysfunction, cognitive impairment with confusion, concentration issues, memory deficits, irritability, restlessness, fatigue, sleep disturbances. Mild visual disturbance and ataxia present. Additional clinical notes include myalgia.
mTBI-04	Temporal: Occipital:	Fusiform Lingual gyrus	+3.0 + 6.0	0.039 5 × 10^−8^	Moderate cervicalgia and photophobia. Mild cephalalgia, ataxia, mild cognitive impairment with confusion, concentration issues, memory deficits, sleep disturbance. Clinical notes include vertigo and confusion at onset.
mTBI-05	Temporal: Occipital:	Middle temporal LOC Lingual gyrus	+3.5 + 3.5 + 3.2	0.007 0.007 0.014	Moderate cognitive impairment, concentration issues, memory deficits, fatigue, sleep disturbance. Mild cephalalgia, phonophobia, photophobia, visual disturbance, ataxia, confusion. Clinical notes include mild vertigo with visuomotor sensitivity and light cranial pressure.
mTBI-15	Temporal:	Middle temporal Entorhinal	+3.0 + 5.7	0.047 3 × 10^−6^	Severe phonophobia, photophobia, vestibular dysfunction, cognitive impairment with confusion, concentration issues, memory deficits, irritability, fatigue, sleep disturbance. Moderate cephalalgia, cervicalgia, vertigo, restlessness. Mild visual disturbance and ataxia.
mTBI-16	Temporal:	Temporal pole	+3.7	0.009	No BIST. Clinical notes include transient mental fog, bradyphrenia, indecisiveness, vestibular dysfunction.
mTBI-17	Temporal:	Temporal pole	+3.4	0.020	Moderate phonophobia, photophobia, visual disturbance, concentration issues. Mild cephalalgia, vertigo, vestibular dysfunction, ataxia, cognitive impairment, memory deficits, irritability, fatigue, sleep disturbance. Clinical notes include disorientation, nausea, and impaired thought.
mTBI-18	Occipital: Cingulate:	Lingual gyrus Posterior cingulate Isthmus, Cingulate	+2.9 + 3.2 + 3.8	0.045 0.021 0.005	Moderate cognitive impairment, concentration issues, fatigue, sleep disturbance. Mild cephalalgia, photophobia, memory deficits, confusion. Clinical notes include being dazed at time of injury.
mTBI-29	Frontal: Parietal: Temporal: Occipital: Insula/cingulate:	Pars triangularis Caudal mPFC Pars opercularis Superior frontal Precentral Superior parietal Inferior parietal Supramarginal Precuneus STS Transverse temporal LOC Insula Rostral ACC	+3.6 + 3.3 + 3.4 + 3.1 + 4.6 + 3.3 + 4.0 + 5.4 + 4.9 + 2.9 + 2.9 + 4.6 + 2.9 + 3.7	0.001 0.004 0.003 0.006 4 × 10^−5^ 0.004 4 × 10^−4^ 3 × 10^−6^ 2 × 10^−5^ 0.011 0.010 4 × 10^−5^ 0.010 0.001	Severe fatigue and sleep disturbance. Moderate cognitive impairment. Mild cephalalgia, photophobia, phonophobia, vestibular dysfunction, memory deficits, concentration issues. Clinical notes include anxiety.
mTBI-30	Frontal:	Caudal mPFC Pars opercularis Superior frontal	+3.7 +3.5 + 3.1	0.007 0.007 0.021	Moderate cephalalgia, photophobia, phonophobia, restlessness, fatigue, sleep disturbance. Mild cervicalgia, vertigo, vestibular dysfunction, ataxia, cognitive impairment, concentration issues, memory deficits. Clinical notes include nausea, reduced tolerance to physical/cognitive exertion, and impaired coordination.
mTBI-34	Frontal: Temporal:	Lateral OFC Middle temporal	+7.2 + 4.6	3 × 10^−12^ 7 × 10^−5^	Moderate cephalalgia, cervicalgia, phonophobia, cognitive impairment, confusion, concentration issues, memory deficits, restlessness, fatigue. Mild photophobia, vertigo, vestibular dysfunction, visual disturbance, ataxia, irritability, sleep disturbance. Clinical notes include being dazed.

Presenting symptoms are derived from BIST injury severity assessments and supplemented with additional clinical patient notes made upon presentation of participants to Axis Sports Medicine clinics. Only participants with abnormal iron profiles are included for brevity and relevance. ID, unique identifier; BIST, Brain Injury Screening Tool; ROI, region of interest; OFC, orbitofrontal cortex; mPFC, middle prefrontal cortex; STS, superior temporal sulcus; LOC, lateral occipital cortex; ACC, anterior cingulate cortex; *p*FDR, statistical significance (*P*-value) after FDR correction.

**Figure 2:**
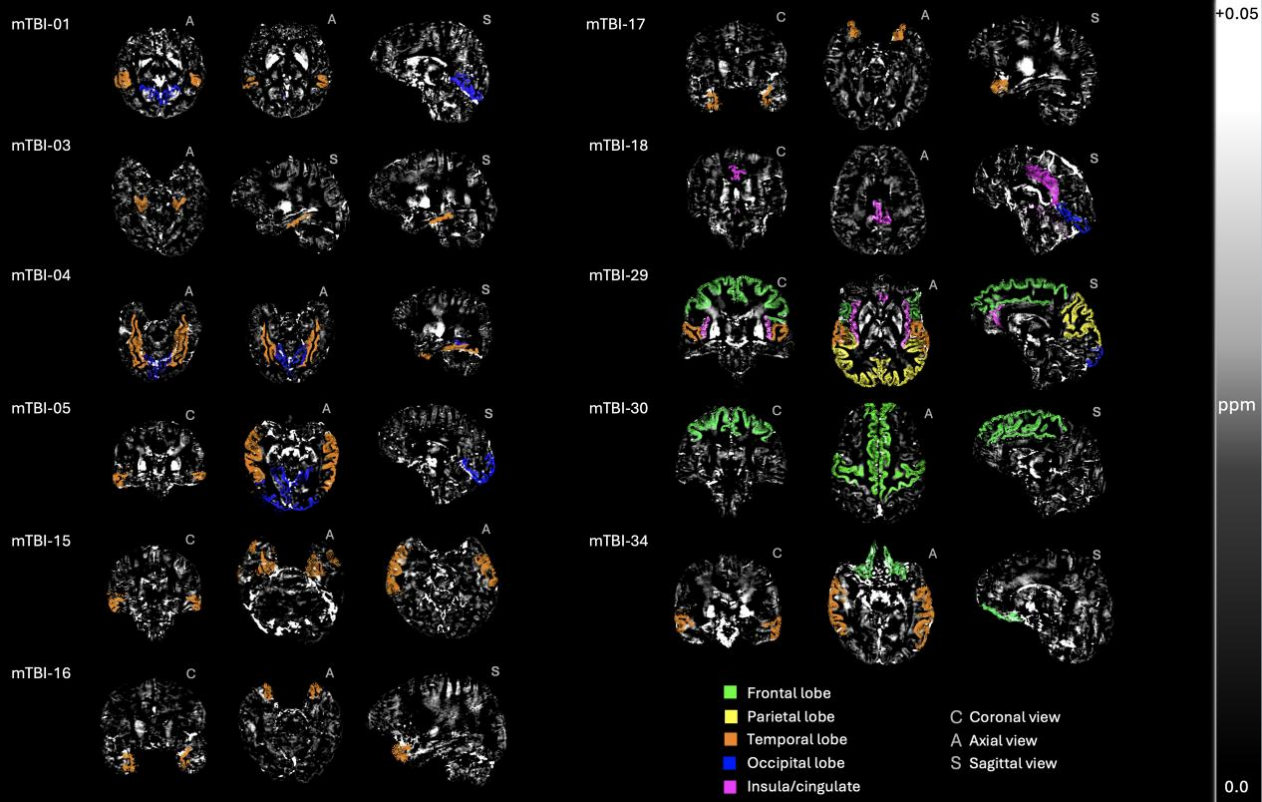
**Individualized profiles of abnormal iron accumulation sites following mTBI.** Visualization of the specific ROIs and lobes where mTBI participants’ (*N* = 1) *Z*-scores significantly deviate from the HC population (*N* = 25–23), highlighting the individualized profiles of iron accumulation following mTBI. The selected orientations (C, coronal; A, axial; S, sagittal) are for best visualization of each participants’ result. These maps have been threshold for positive susceptibility values (iron-related) and are expressed in parts per million (ppm) from 0.0 to +0.05. Z-scores and correspondent *P*-values (after FDR correction: *p*FDR) are detailed in Table 2.

In these 11 clinical participants, injury-related elevated susceptibility was evident across all cortical lobes, however, these were predominantly localized to either a single lobe (45%) or two lobes (45%). Only one participant (9%) exhibited widespread, multi-focal abnormalities across the cortex (see Table 2 and Fig. 2). Notably, a high density of affected ROIs was observed in the temporal lobe for 82% (9 out of 11) of participants with abnormal iron profiles (see Table 2). In contrast, 45% (5/11) had abnormal iron in occipital ROIs, 27% (3/11) in frontal ROIs, 18% (2/11) in the insula or cingulate and only one participant (9%) had an abnormal profile inclusive of parietal ROIs.

**Figure 3:**
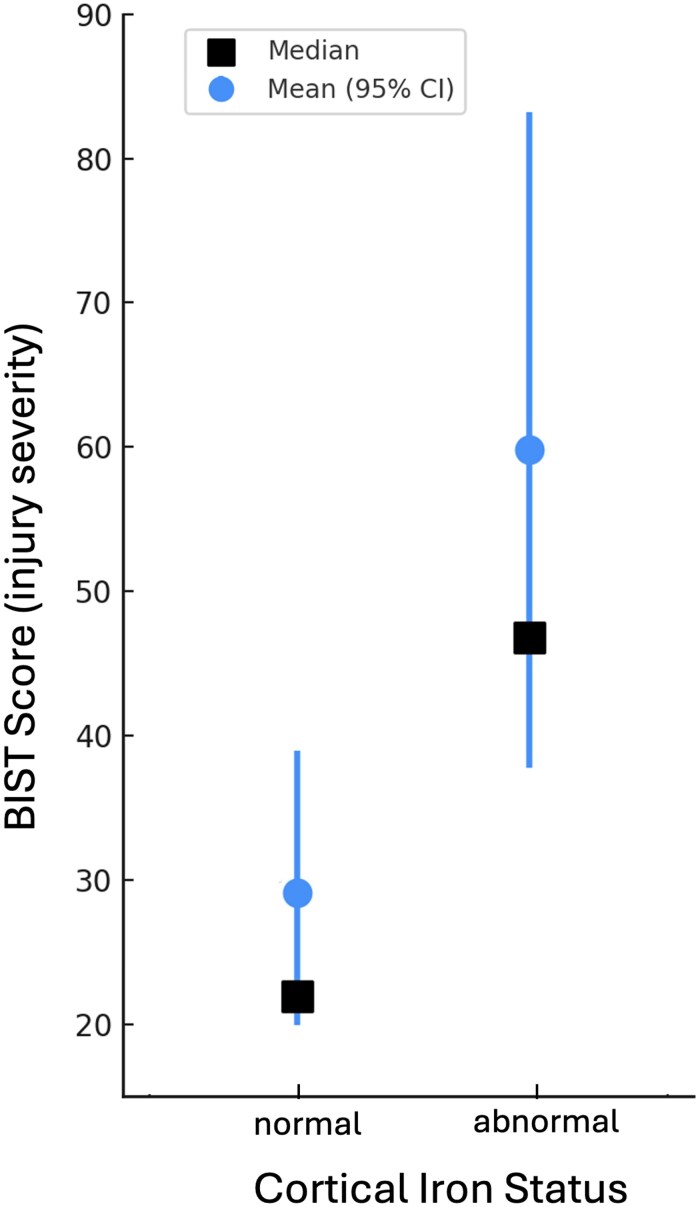
**BIST scores by cortical iron status.** A Mann–Whitney U-test revealed that injury severity scores were significantly higher for iron-abnormal (*N* = 10; *M* = 59.6, SD = 40.5) than iron-normal (*N* = 24; *M* = 29.2, SD = 23.3) mTBI participants, *U* = 57, *P* = 0.02. Median and mean scores are depicted alongside 95% CI.

After subdividing the mTBI participants into iron-normal (24/35; 69%) and iron-abnormal (11/35; 31%) based on their individual ROI-wise profiles, a one-way ANOVA showed no significant effect of age between these groups and controls, *F*(2, 26) = 2.0, *P* = 0.2. The Mann–Whitney U-test revealed that injury severity (BIST^53^) scores were significantly worse for the iron-abnormal mTBI group (*M* = 59.6, SD = 40.5) than the iron-normal mTBI group (*M* = 29.2, SD = 23.3), *U* = 57, *P* = 0.02 (see Fig. 3).

### Symptoms and cortical iron-related markers

To provide a descriptive overview of cortical iron profiles and corresponding symptomatology (see Table 2), we note observational relationships between abnormalities in the lingual gyrus (four participants) and cephalalgia, photophobia, cognitive impairment (including confusion), concentration issues, memory deficits and sleep disturbances. The middle temporal region (three participants) appeared to be linked to a broader symptom profile, including cognitive impairment (with confusion), concentration issues, memory deficits, fatigue, sleep disturbances, cephalalgia, phonophobia, photophobia, vertigo, ataxia and visual disturbances. Although other ROIs were less commonly affected (less than or equal to two participants), some descriptive inferences include the involvement of the superior temporal region [including the superior temporal sulcus (STS) and its bank] with cognitive impairment, headaches, photophobia, phonophobia, vestibular dysfunction, memory deficits, concentration issues, fatigue, sleep disturbances, and in one participant, severe tinnitus and irritability.

### Secondary depth- and curvature-specific iron profiles

**Figure 4:**
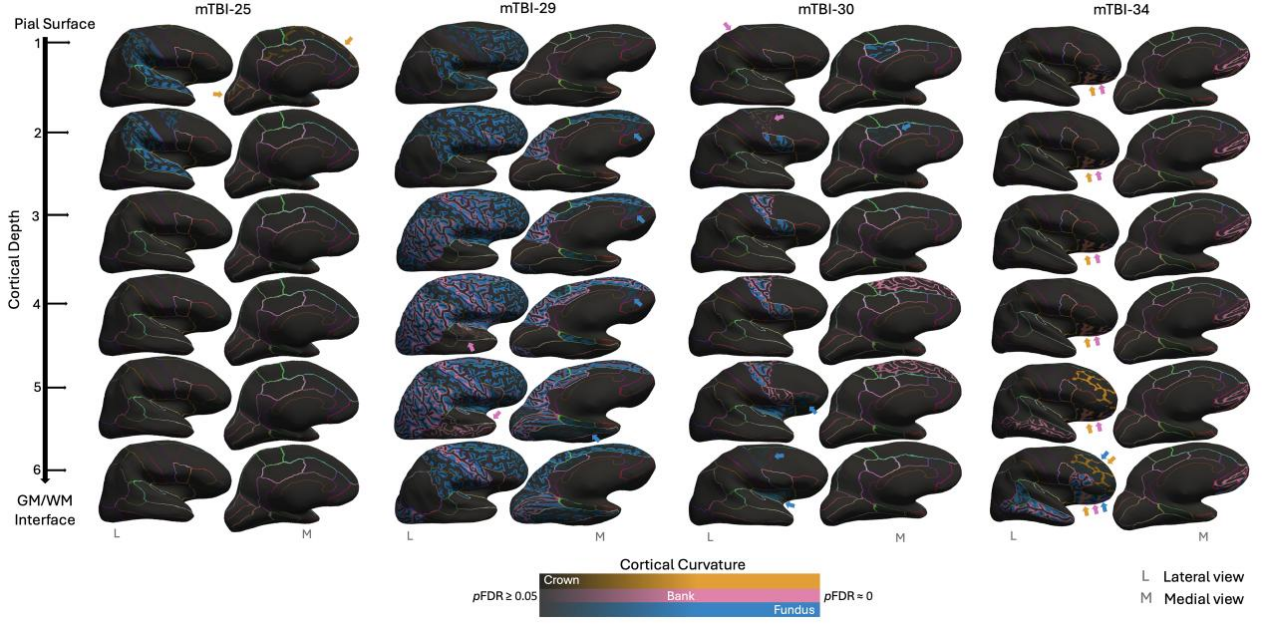
**Cortical depth- and curvature-specific profiles of mTBI-related abnormal iron accumulation.** Cortical depth- and curvature-specific iron profiles across five representative sr-mTBI participants. Inflated surfaces show each of the six cortical depths, from the pial surface (depth 1) to the GM/WM interface (depth 6). Regions of abnormal iron deposition are colour-coded according to cortical curvature. Colour intensity shows level of statistical significance (*p*FDR < 0.05). Regions with lower statistical significance that are harder to visually distinguish are indicated by arrows in the colour of the correspondent cortical curvature. Lateral (L) and medial (M) views are used to visualize the whole brain. Boundaries between ROIs are delineated using coloured lines. To derive *Z*-scores, individual (*N* = 1) mTBI participants’ data were compared to the HC normal distribution (*N* = 25), at each depth (*N* = 6), for each curvature (*N* = 3), within each ROI (*N* = 34). Full statistical results, including *Z*-scores and *P*-values (after FDR correction) are available in Supplementary Data 1. Abbreviations are as follows: *p*FDR = statistical significance (*P*-value) after FDR correction.

Secondary exploratory analyses sensitive to six cortical depths and three curvatures further highlighted the heterogeneity of iron deposition in mTBI. Only 17% (6/35) of participants retained normal iron profiles; the remaining 83% (29/35) showed elevated susceptibility in at least one ROI, for at least one depth and for at least one curvature. Isolated instances of negative *Z*-scores (indicating lower iron compared to HC) were observed in 7 of the 29 participants but were typically limited to a single ROI/depth combination. Overall, abnormal iron accumulation was most pronounced in the sulcal fundus, followed by the sulcal bank, and was least evident in the gyral crown. However, there was significant inter-individual variability in ROI/curvature/depth combinations (see Fig. 4).

For the 11 participants who demonstrated increased iron in the whole-ROI analyses, a similar ROI-wise distribution of elevated iron was observed in depth- and curvature-specific analyses. For example, in the whole-ROI analysis mTBI-30 showed elevated susceptibility in the caudal mPFC, pars opercularis and superior frontal gyrus (see Table 2 and Fig. 2), which was localized to specific depths and curvatures in the secondary analyses. In addition, regions that did not appear in the initial analyses, including the insula, lateral occipital cortex (LOC), pars triangularis, posterior cingulate, precentral and superior parietal areas, exhibited elevated iron scores in depth- and curvature-specific analyses (see Fig. 4). Conversely, mTBI-25 exemplifies a case where no abnormal iron profile was detected in the whole-ROI analysis but became evident in the analyses sensitive to anatomical morphology (see Fig. 4).

After grouping mTBI participants by iron status based on secondary depth- and curvature-specific results, a Mann–Whitney U-test revealed no significant difference in BIST scores between iron-abnormal (*M* = 40.6, SD = 33.5) and iron-normal (*M* = 26.3, SD = 22.4) mTBI participants, *U* = 63, *P* = 0.35. An ANOVA showed no significant difference in age between these groups and controls, *F*(2, 15) = 1.11, *P* = 0.36.

## Discussion

Previous studies using QSM to examine the role of brain iron following mTBI have primarily focused on subcortical brain areas or global grey and/or WM.^36-44^ Only three investigations have included cortical ROIs and, of these, only our previous work^45^ accounted for anatomical variations in cortical depth and curvature. However, these studies relied on group-level statistical analyses, which can obscure individual brain changes due to the subtle nature of cell damage associated with mTBI. This approach may limit our understanding of this heterogeneous injury, hindering the implementation of individualized rehabilitation strategies and treatments. We believe, therefore, that research incorporating comparisons of individual clinical participant data to healthy normative ranges can play a key role in informing targeted neural and pharmacological interventions. We conducted the first investigation of individual differences in cortical magnetic susceptibility after sr-mTBI across 34 cortical ROIs, using a healthy population template as a reference. Secondary exploratory analyses sensitive to cortical depth and curvature were included to better characterize injury profiles for each participant.

Our findings revealed that a substantial subset of individuals with mTBI exhibit elevated levels of cortical magnetic susceptibility, indicating injury-related iron accumulation. Our primary investigation evidenced abnormal iron profiles in just under one-third (31%; 11/35) of participants relative to the HC population. Additionally, we found that mTBI participants with abnormal iron accumulation identified in primary ROI-wise analyses experienced more severe symptoms. In these 11 iron-abnormal clinical participants, elevated iron profiles were predominantly focal to specific lobes or bi-focal (affecting two lobes). Only one participant exhibited multi-focal cortex-wide elevated *Z*-scores. The high density of affected temporal lobe ROIs indicates that, despite significant inter-subject variability, iron accumulation following mTBI is preferential to temporal regions. Taken together, our findings support an iron-related mechanism of secondary injury that modulates symptom severity and may influence symptom presentation.

Iron dyshomeostasis in the basal nuclei is known to impair cognitive function after mTBI,^16,17^ however, little is known about the effect of ‘cortical’ iron aggregation on mTBI symptomatology or severity. In our previous work,^45^ we reported few correlations between regions of cortical iron accumulation and BIST^53^ scores, a measure of injury severity and dominant symptom cluster. This may be accounted for by variability in the accuracy of symptom reporting or purposeful underreporting of symptoms, a common phenomenon among sports players.^86^ However, group-level examinations may also obscure individual differences and inhibit the implementation of more targeted statistical approaches. By assessing the effect of mTBI at the individual level, we were able to facilitate precise between group analyses of injury severity that differentiated between iron-normal and iron-abnormal mTBI participants. Results from our primary analysis revealed a significantly higher symptom burden for participants with mTBI when their iron profiles were also abnormal.

The field generally lacks reliable correlations between subjective assessments of injury severity and objective measures of brain injury and recovery,^87^ as well as alignment between cognitive or clinical findings and neuroimaging results.^88^ Identifying reliable, objective markers of structural changes that are related to subjective self-reported symptoms is crucial because individual variations in brain injury location and severity can lead to disparate clinical presentations and recovery trajectories but may be missed in group-level analyses.^8^ Although research indicates that most individuals recover well from mTBI, between 15^11^ and 30%^8^ of patients experience significant, and in some cases life-changing, long-term clinical sequelae. Understanding the underlying pathophysiological drivers of these poorer outcomes is essential for enabling precise, patient-specific clinical interventions. Our finding that 31% of participants exhibited abnormal iron profiles substantial enough to be detected in the primary ROI-wise analyses, which were linked to poorer outcomes, aligns with both evidence of structural brain changes detected with advanced MRI in up to 30% of mTBI cases,^1^ as well as evidence from individualized studies reporting a similar percentage (28%) of subacute-phase WM anomalies and associations with worse cognitive outcomes in ms-TBI.^51^ In addition, standardized susceptibility values in the basal nuclei are reported to correlate with mTBI symptom duration, but only in a sub-group of participants with persistent symptomatology for at least a week.^40^ In keeping with these findings, our results reinforce the importance of individualized analyses in revealing not only associations between the extent of microstructural pathology and negative outcomes following mTBI, but for identifying at-risk cohorts. Here, the value of personalized approaches to understanding mTBI becomes strikingly apparent. Further longitudinal studies that track participants through recovery could help to determine whether elevated ROI-wise cortical iron levels are associated with prolonged recovery, persistent post-concussive symptoms, or adverse outcomes later in life. Such research may offer insights into why a subset of individuals with mTBI fails to recover fully.

The promise of individualized assessments to identify biomarkers for mild brain injury is particularly salient given the current absence of objective markers for mTBI diagnosis. Diagnostic decisions are limited to subjective self-report and assessments of physiological function,^13,87^ as the heterogeneity of mTBI complicates efforts to identify reliable biomarkers or imaging signatures that can be applied universally across patients. Whether cortical iron accumulation reflects inflammatory processes, BBB disruption,^9^ ferroptosis,^31,89^ or other cytotoxic processes is beyond the scope of the current research. However, we posit that the significantly higher symptom burden (BIST^53^ score) observed in the iron-abnormal mTBI cohort, as identified via whole-ROI analyses, supports an iron-mediated mechanism of brain changes related to injury severity and functional impairment, and marks iron as a promising mTBI biomarker. Of particular note, many of the symptoms observed in mTBI resemble those seen in other iron-mediated neurodegenerative processes, such as cognitive decline in normal aging,^90^ and the cognitive and motor dysfunctions characteristic of diseases like Alzheimer’s, Huntington’s, Parkinson’s and Friedrich’s ataxia, as well as multiple sclerosis, where iron dysregulation is a hallmark feature.^9,91^ Genetic disorders of iron overload, such as neuroferritinopathy, also present with cognitive and motor symptoms.^92^

The susceptibility of the temporal lobe to iron accumulation post-mTBI, evident in this and previous research,^45^ aligns with the memory impairments characteristic of mTBI.^93^ Individual-level data from our mTBI sample emphasizes the link between high temporal iron accumulation and memory deficits, as exemplified by BIST^53^ scores related to memory (see Table 2). In addition, specific distributions of p-tau focal to temporal (and frontal) cortex are considered features of chronic traumatic encephalopathy (CTE),^94,95^ an mTBI-related neurodegenerative disorder. Of particular concern, co-localization of iron with p-tau in CTE has been highlighted in histological examinations.^34^ Whilst the precise relationship between iron overload and the downstream hyperphosphorylation of tau proteins remains an active area of research, further exploration of the interplay between acute cortical iron elevation, symptom burden, temporal recovery dynamics and long-term brain health outcomes is warranted.

The diversity of mechanistic antecedents, pathological mechanisms and clinical outcomes associated with mTBI reflects the complex underlying pathophysiology.^8^ mTBI is not a uniform injury and structural indicators, such as those commonly observed in other forms of TBI, do not always correlate with clinical symptoms or outcomes.^96^ Here, we speculate that abnormal iron accumulation in specific cortical ROIs may be related to participant symptomatology. For instance, the STS plays a key role in social cognition, empathy, mentalising about others’ emotional states and ‘theory of mind.’^97-99^ Structural changes to this region may explain the severe irritability reported by mTBI-01 (see Table 2), along with complaints of severe tinnitus.^100^ As a hub for audiovisual integration,^101^ the superior temporal region could also be involved in phonophobia (sound sensitivity), visual disturbances and vestibular dysfunction^102^; symptoms experienced by both mTBI participants whose profiles indicated local iron elevation. Similarly, the lingual gyrus is active during migraine episodes and responds to luminous stimuli, suggesting its involvement in photophobia, visual processing anomalies and cephalalgia (headache).^103^ These symptoms were observed in all clinical participants with iron aggregation in this region (see Table 2). Our findings allude to specific areas of cortical iron accumulation that show a relationship to clinical sequelae, suggesting that regions with higher iron burden may be evidence of microstructural cell damage that disrupts normal function. Here, it should be noted that multiple cortical regions with abnormal iron markers were observed for most mTBI participants, making it challenging to delineate a one-to-one relationship between a specific ROI and symptoms reported. As such, future research should integrate functional MRI to improve the mapping of structural changes to deficits in functional connectivity and well-established brain networks that may overlap with abnormalities in cortical grey matter regions. Without additional research, observations about regions of iron accumulation and symptom presentation are speculative and less convincing than case-matching between brain lesions in gross TBI and neurobehavioural symptom presentation.^104^

### Depth- and curvature-specific iron accumulation

Secondary exploratory analyses revealed significant inter-individual heterogeneity in depth- and curvature-specific cortical iron accumulation. Although these subtle effects were not associated with injury severity, they provide evidence of widespread minor tissue alterations following mTBI. A consistent trend for iron deposition was observed in the sulci, with the highest concentration at the fundus, followed by the sulcal banks. This pattern may be attributed to the heightened vulnerability of the fundus to mTBI-related injury, which is susceptible to mechanical deformation due to the ‘water hammer effect,’ where CSF is forced into the depths of the sulci, causing local damage.^105^ Supporting this, previous research has demonstrated that mTBI increases cortical curvature in the sulcus,^106^ as well as widens the sulci and causes focal vascular injury and microhaemorrhages in the fundus, as evidenced by susceptibility-weighted imaging.^105^ Conversely, in more severe TBI, contusions are often concentrated at the gyri,^93^ suggesting that gyral iron accumulation in ‘mild’ TBI may represent a less severe version of this type of injury. An understanding of injury biomechanics may thus prove crucial to explaining variance in loci of neuropathological changes. Research shows that different types of head impacts can result in varying brain deformations and injury patterns, with sulci being particularly vulnerable to mechanical strain, which is consistent with, and can be predicted by, patterns of tauopathy observed in neurodegenerative conditions.^107^ Personalized iron accumulation patterns may provide insight into the specific injury mechanisms and related cellular disruption experienced by each individual participant at the acute stage. Elucidating this link, as well as how this may be related to mTBI-induced neuropathology in later life, should be a focus for future research.

Histological studies, including Perl’s iron staining and ultra-high-field (7T) R_2_* mapping of tissue samples^108^ have localized iron deposition to specific cortical layers, reflecting distinct cyto- and myelo-architectural features with layer-specific distributions that show congruence with *in vivo* QSM.^109^ In healthy populations, iron concentrations typically increase from the pial surface towards the GM/WM boundary; deviations away from baselines for each layer suggest an injury-specific model of cortical cellular trauma. For instance, layer I primarily contains axons and dendrites, with the cell bodies in deeper layers^110^; iron accumulation in different layers may point to diverse pathologies affecting different parts of the cell. Depth-wise patterns may also be related to injury biomechanics: superficial iron accumulation may be a result of perivascular trauma, which is often linked to microhaemorrhages and microglial activation after mTBI,^27,28,93^ whereas deeper iron deposition may reflect more severe shear forces, which are known to cause significant damage near the GM/WM interface in mTBI.^111^ This is supported by computational modelling showing that shear forces are concentrated in this region,^107^ which is also a common site of microbleeds.^112^ Tying this in with investigations of cortical curvature, these effects would likely be concentrated at the sulcal fundus.^105,107,113^ However, evidence from TBI research supports contusions of the gyri that often follow a layer-specific pattern, with damage prominent at the superficial crest but extending through the cortical mantle to the GM/WM boundary in a ‘wedge’ of haemorrhage and necrotic tissue.^93^ Iron-dependent cell death, ferroptosis,^31,89^ could plausibly account for instances of iron-related gyral pathology in mTBI that exhibit similar depth-specific patterns.

It is important to note that both injuries at the pial surface^113^ and closer to the GM/WM border^111^ have been related to adverse outcomes after mTBI. However, subpial iron deposition near small blood vessels in the fundus is congruent with distributions of sulcal tauopathy seen in CTE,^113^ which may be related to breaches in the BBB.^107^ Injury-induced microvascular dysfunction may increase BBB permeability^114,115^ which likely increases active transport of non-heme iron via vascular endothelial cells into the superficial layers of the cortex^9,29,116^ which could account for perivascular accumulations.^93,94^ The potential long-term effects of iron deposition in this young cohort warrants further investigation, given the known relationship between iron accumulation and tau pathology.^34^

### Limitations and future research

The ability of neuroimaging modalities to infer underlying biological processes is limited by spatial constraints and inference about tissue composition from indirect markers. While the use of QSM can provide insight into potential iron accumulation, it remains a surrogate measure of iron-tissue content. Integrating complementary modalities such as PET, or protein assays, would offer a more comprehensive understanding of the underlying neurobiology. Additionally, because analyses related to cortical thickness involve sampling multiple tissue types across a small cross-section, higher resolutions become particularly important.^117^ Although ≤1 mm isotropic resolution is recommended to mitigate partial volume effects,^64^ such artefacts may be problematic at the cortical surface and GM/WM tissue interface. While erosion steps were used to reduce non-brain signals around the outer cortex, acquiring higher-resolution images, assuming an adequate field-of-view,^60^ may further reduce partial volume effects. It is worth noting that QSM studies conducted at similar resolution found that discarding the outer depths did not significantly alter susceptibility measurements,^60^ suggesting that the influence of partial volume-induced distortions may be relatively modest under these conditions. The 1 mm isotropic voxel resolution also inherently restricts depth-wise analysis of the ∼1–4.5 mm thick cerebral cortex.^83^ However, studies employing comparable depth-specific methodologies have utilized data from QSM,^60^ DTI,^76^ T1w/T2w imaging^118^ and quantitative T1 and QSM,^119^ to sample between 10 and 21 cortical depths with meaningful results. In addition, the DTI-based study^76^ reported the identification of features with 0.9 mm isotropic resolution, single-shell data similar to those found with 92 µm isotropic, high-angular-resolution data,^120^ underscoring the ability of these technique to conserve features even at coarser resolutions. Whilst the model presented here is more conservative than previous approaches, sampling fewer depths (e.g. three) could arguably capture overall cortical thickness and avoid redundancy; however, sampling fewer depths appears to forfeit distinct susceptibility features uniquely revealed by a six-depth model (see Supplementary Figs 1–34). Given inter-individual variability in cortical thickness, there is need for individualized approaches that are sensitive to heterogeneity. These inherent constraints should nonetheless be considered when interpreting the presented data.

It should be noted that this study did not control for several potential confounding factors, including prior injuries, genetic predispositions and environmental influences, all known to affect injury severity and recovery trajectories.^8^ Moreover, natural age-related increases in cortical iron are particularly pronounced within this specific age range.^121^ In principle, within a sample so closely age-matched, the normal distribution reflects typical variation in cortical susceptibility values, but age effects may still not be fully accounted for. The power of *Z*-scores to regress these variables is limited, particularly at small sample sizes and future research should consider recruiting large control cohorts that enable more robust approaches, such as normative modelling.^122^ This would also confer the added benefit of sampling across broader populations, simultaneously improving generalizability. This is particularly salient for female athletes, for whom hormonal variations,^123^ the use of oral contraceptives^124^ and differences in neck musculature^125^ lend themselves to further heterogeneity in injury severity and outcome. In lieu of normative modelling, investigations using female cohorts are planned to address this gap. Lastly, the current study relied on a single-echo QSM sequence, constraining susceptibility thresholding to ‘between’ voxels. Multi-echo sequences enable the separation of susceptibility sources ‘within’ voxels,^109,126,127^ and should be considered for future research.

To conclude, our results suggest that iron-mediated cell damage plays a key role in mTBI pathology. In keeping with the heterogeneity of mTBI, accumulation of cortical iron after injury is person-specific and may be influenced by cortical morphology. These results highlight the importance of examining mTBI at an individual level rather than relying on group-level analyses. This variability likely also complicates the search for universal biomarkers, further underscoring the need for personalized approaches that integrate advanced imaging and detailed symptom profiling.

## Supplementary Material

fcaf110_Supplementary_Data

## Data Availability

De-identified MRI data can be made available upon request to the corresponding author (C.A.E.). Codes used for *Z*-score generation and analysis are available on the GitHub of the corresponding author (https://github.com/ChristiEssex/QSMindividualised). Parent codes for cortical column generation are hosted on GitHub (https://github.com/devonko) and can be available upon request from co-authors (J.L.M. and T.-K.T.) based at the Brain Imaging and Analysis Center at Duke University Medical Center. QSM data were generated using QSMxT (https://qsmxt.github.io/QSMxT/). Additional data processing, including pial surface estimation, was conducted with FreeSurfer (https://surfer.nmr.mgh.harvard.edu).

## References

[1] Maas AI, Menon DK, Manley GT, et al Traumatic brain injury: Progress and challenges in prevention, clinical care, and research. Lancet Neurol. 2022;21(11):1004–1060. 36183712 10.1016/S1474-4422(22)00309-X PMC10427240

[2] Howe EI, Andelic N, Fure SC, et al Cost-effectiveness analysis of combined cognitive and vocational rehabilitation in patients with mild-to-moderate TBI: Results from a randomized controlled trial. BMC Health Serv Res. 2022;22(1):185. 35151285 10.1186/s12913-022-07585-3 PMC8840547

[3] Guskiewicz KM, Marshall SW, Bailes J, et al Recurrent concussion and risk of depression in retired professional football players. Med Sci Sports Exerc. 2007;39(6):903–909. 17545878 10.1249/mss.0b013e3180383da5

[4] McInnes K, Friesen CL, MacKenzie DE, et al Mild traumatic brain injury (mTBI) and chronic cognitive impairment: A scoping review. PLoS One. 2017;12(4):e0174847. 28399158 10.1371/journal.pone.0174847 PMC5388340

[5] Mackay DF, Russell ER, Stewart K, et al Neurodegenerative disease mortality among former professional soccer players. N Engl J Med. 2019;381:1801–1808. 31633894 10.1056/NEJMoa1908483 PMC8747032

[6] De Beaumont L, Thoret H, Mongeon D, et al Brain function decline in healthy retired athletes who sustained their last sports concussion in early adulthood. Brain. 2009;132(3):695–708. 19176544 10.1093/brain/awn347

[7] Guskiewicz KM, Marshall SW, Bailes J, et al Association between recurrent concussion and late-life cognitive impairment in retired professional football players. Neurosurgery. 2005;57(4):719–726. 16239884 10.1093/neurosurgery/57.4.719

[8] Rosenbaum SB, Lipton ML. Embracing chaos: The scope and importance of clinical and pathological heterogeneity in mTBI. Brain Imaging Behav. 2012;6(2):255–282. 22549452 10.1007/s11682-012-9162-7

[9] Gozt A, Hellewell S, Ward PG, et al Emerging applications for quantitative susceptibility mapping in the detection of traumatic brain injury pathology. Neuroscience. 2021;467:218–236. 34087394 10.1016/j.neuroscience.2021.05.030

[10] Giza CC, Hovda DA. The new neurometabolic cascade of concussion. Neurosurgery. 2014;75(Suppl 4):S24–S33. 25232881 10.1227/NEU.0000000000000505 PMC4479139

[11] Walker KR, Tesco G. Molecular mechanisms of cognitive dysfunction following traumatic brain injury. Front Aging Neurosci. 2013;5:29. 23847533 10.3389/fnagi.2013.00029 PMC3705200

[12] Hier DB, Obafemi-Ajayi T, Thimgan MS, et al Blood biomarkers for mild traumatic brain injury: A selective review of unresolved issues. Biomark Res. 2021;9(1):70. 34530937 10.1186/s40364-021-00325-5 PMC8447604

[13] Lunkova E, Guberman GI, Ptito A, et al Noninvasive magnetic resonance imaging techniques in mild traumatic brain injury research and diagnosis. Hum Brain Mapp. 2021;42(16):5477–5494. 34427960 10.1002/hbm.25630 PMC8519871

[14] Cook GA, Hawley JS. A review of mild traumatic brain injury diagnostics: Current perspectives, limitations, and emerging technology. Mil Med. 2014;179(10):1083–1089. 25269125 10.7205/MILMED-D-13-00435

[15] Wintermark M, Sanelli PC, Anzai Y, et al Imaging evidence and recommendations for traumatic brain injury: Advanced neuro- and neurovascular imaging techniques. AJNR Am J Neuroradiol. 2015;36(2):E1–E11. 25424870 10.3174/ajnr.A4181 PMC7965673

[16] Lu L, Cao H, Wei X, et al Iron deposition is positively related to cognitive impairment in patients with chronic mild traumatic brain injury: Assessment with susceptibility weighted imaging. Biomed Res Int. 2015;2015:470676. 26798636 10.1155/2015/470676 PMC4698517

[17] Raz E, Jensen JH, Ge Y, et al Brain iron quantification in mild traumatic brain injury: A magnetic field correlation study. AJNR Am J Neuroradiol. 2011;32(10):1851–1856. 21885717 10.3174/ajnr.A2637 PMC3848044

[18] Duyn JH, Schenck J. Contributions to magnetic susceptibility of brain tissue. NMR Biomed. 2017;30(4):10.1002/nbm.3546. 10.1002/nbm.3546 PMC5131875 27240118

[19] Jang J, Nam Y, Jung SW, et al Paradoxical paramagnetic calcifications in the globus pallidus: An ex vivo MR investigation and histological validation study. NMR Biomed. 2021;34(10):e4571. 34129267 10.1002/nbm.4571

[20] Kim S, Lee Y, Jeon CY, et al Quantitative magnetic susceptibility assessed by 7T magnetic resonance imaging in Alzheimer’s disease caused by streptozotocin administration. Quant Imaging Med Surg. 2020;10(3):789–797. 32269937 10.21037/qims.2020.02.08 PMC7136729

[21] Wang Y, Spincemaille P, Liu Z, et al Clinical quantitative susceptibility mapping (QSM): Biometal imaging and its emerging roles in patient care. J Magn Reson Imaging. 2017;46(4):951–971. 28295954 10.1002/jmri.25693 PMC5592126

[22] Gong NJ, Dibb R, Bulk M, et al Imaging beta amyloid aggregation and iron accumulation in Alzheimer’s disease using quantitative susceptibility mapping MRI. NeuroImage. 2019;191:176–185. 30739060 10.1016/j.neuroimage.2019.02.019

[23] Zhao Z, Zhang L, Wen Q, et al The effect of beta-amyloid and tau protein aggregations on magnetic susceptibility of anterior hippocampal laminae in Alzheimer’s diseases. NeuroImage. 2021;244:118584. 34537383 10.1016/j.neuroimage.2021.118584

[24] Langkammer C, Schweser F, Krebs N, et al Quantitative susceptibility mapping (QSM) as a means to measure brain iron? A post mortem validation study. NeuroImage. 2012;62(3):1593–1599. 22634862 10.1016/j.neuroimage.2012.05.049 PMC3413885

[25] Liu C, Li W, Tong KA, et al Susceptibility-weighted imaging and quantitative susceptibility mapping in the brain. J Magn Reson Imaging. 2015;42(1):23–41. 25270052 10.1002/jmri.24768 PMC4406874

[26] Ravanfar P, Loi SM, Syeda WT, et al Systematic review: Quantitative susceptibility mapping (QSM) of brain iron profile in neurodegenerative diseases. Front Neurosci. 2021;15:618435. 33679303 10.3389/fnins.2021.618435 PMC7930077

[27] Huang S, Li S, Feng H, et al Iron metabolism disorders for cognitive dysfunction after mild traumatic brain injury. Front Neurosci. 2021;15:587197. 33796002 10.3389/fnins.2021.587197 PMC8007909

[28] Nisenbaum EJ, Novikov DS, Lui YW. The presence and role of iron in mild traumatic brain injury: An imaging perspective. J Neurotrauma. 2014;31(4):301–307. 24295521 10.1089/neu.2013.3102 PMC3922137

[29] Levi S, Ripamonti M, Moro AS, et al Iron imbalance in neurodegeneration. Mol Psychiatry. 2024;29:1139–1152. 38212377 10.1038/s41380-023-02399-z PMC11176077

[30] Daglas M, Adlard PA. The involvement of iron in traumatic brain injury and neurodegenerative disease. Front Neurosci. 2018;12:981. 30618597 10.3389/fnins.2018.00981 PMC6306469

[31] Tang S, Gao P, Chen H, et al The role of iron, its metabolism and ferroptosis in traumatic brain injury. Front Cell Neurosci. 2020;14:590789. 33100976 10.3389/fncel.2020.590789 PMC7545318

[32] Neuwelt E, Abbott J, Abrey L, et al Strategies to advance translational research into brain barriers. Lancet Neurol. 2008;7(1):84–96. 18093565 10.1016/S1474-4422(07)70326-5

[33] Morganti-Kossmann MC, Satgunaseelan L, Bye N, et al Modulation of immune response by head injury. Injury. 2007;38(12):1392–1400. 18048036 10.1016/j.injury.2007.10.005

[34] Bouras C, Giannakopoulos P, Good PF, et al A laser microprobe mass analysis of brain aluminum and iron in dementia pugilistica: Comparison with Alzheimer’s disease. Eur Neurol. 1997;38(1):53–58. 9252800 10.1159/000112903

[35] Zecca L, Youdim MB, Riederer P, et al Iron, brain ageing and neurodegenerative disorders. Nat Rev Neurosci. 2004;5:863–873. 15496864 10.1038/nrn1537

[36] Bell TK, Ansari M, Joyce JM, et al Quantitative susceptibility mapping in adults with persistent-post concussion symptoms after mild traumatic brain injury: An exploratory study. AJNR Am J Neuroradiol. 2025;46(2):435–442. 39151958 10.3174/ajnr.A8454 PMC11878970

[37] Brett BL, Koch KM, Muftuler LT, et al Association of head impact exposure with white matter macrostructure and microstructure metrics. J Neurotrauma. 2021;38(4):474–484. 33003979 10.1089/neu.2020.7376 PMC7875606

[38] Gong NJ, Kuzminski S, Clark M, et al Microstructural alterations of cortical and deep gray matter over a season of high school football revealed by diffusion kurtosis imaging. Neurobiol Dis. 2018;119:79–87. 30048802 10.1016/j.nbd.2018.07.020

[39] Koch KM, Meier TB, Karr R, et al Quantitative susceptibility mapping after sports-related concussion. AJNR Am J Neuroradiol. 2018;39(7):1215–1221. 29880474 10.3174/ajnr.A5692 PMC6055518

[40] Koch KM, Nencka AS, Swearingen B, et al Acute post-concussive assessments of brain tissue magnetism using magnetic resonance imaging. J Neurotrauma. 2021;38(7):848–857. 33066712 10.1089/neu.2020.7322 PMC8432603

[41] Pinky NN, Debert CT, Dukelow SP, et al Multimodal magnetic resonance imaging of youth sport-related concussion reveals acute changes in the cerebellum, basal ganglia, and corpus callosum that resolve with recovery. Front Hum Neurosci. 2022;16:976013. 36337852 10.3389/fnhum.2022.976013 PMC9626521

[42] Weber AM, Pukropski A, Kames C, et al Pathological insights from quantitative susceptibility mapping and diffusion tensor imaging in ice hockey players pre and post-concussion. Front Neurol. 2018;9:575. 30131752 10.3389/fneur.2018.00575 PMC6091605

[43] Wright DK, O’Brien TJ, Shultz SR. Sub-acute changes on MRI measures of cerebral blood flow and venous oxygen saturation in concussed Australian rules footballers. Sports Med Open. 2022;8:45. 35362855 10.1186/s40798-022-00435-w PMC8975948

[44] Zivadinov R, Polak P, Schweser F, et al Multimodal imaging of retired professional contact sport athletes does not provide evidence of structural and functional brain damage. J Head Trauma Rehabil. 2018;33(5):E24–E32. 10.1097/HTR.0000000000000422 PMC6126951 30080799

[45] Essex CA, Merenstein JL, Overson DK, et al Characterizing positive and negative quantitative susceptibility values in the cortex following mild traumatic brain injury: a depth- and curvature-based study. Cereb Cortex. 2025;35(3):bhaf059. 10.1093/cercor/bhaf059 40099836 PMC11915090

[46] Mito R, Pedersen M, Pardoe H, et al Exploring individual fixel-based white matter abnormalities in epilepsy. Brain Commun. 2023;6(1):fcad352. 38187877 10.1093/braincomms/fcad352 PMC10768884

[47] Domínguez JF, Stewart A, Burmester A, et al Improving quantitative susceptibility mapping for the identification of traumatic brain injury neurodegeneration at the individual level. Z Med Phys. Published online 8 February 2024. doi: 10.1016/j.zemedi.2024.01.00138336583

[48] Bedggood MJ, Essex CA, Theadom A, et al Individual-level analysis of MRI T2 relaxometry in mild traumatic brain injury: Possible indications of brain inflammation. Neuroimage Clin. 2024;43:103647. 39068788 10.1016/j.nicl.2024.103647 PMC11663787

[49] Attye´ A, Renard F, Baciu M, et al TractLearn: A geodesic learning framework for quantitative analysis of brain bundles. NeuroImage. 2021;233:117927. 33689863 10.1016/j.neuroimage.2021.117927

[50] Clemente A, Attye´ A, Renard F, et al Individualised profiling of white matter organisation in moderate-to-severe traumatic brain injury patients using TractLearn: A proof-of-concept study. Brain Res. 2023;1806:48289. 10.1016/j.brainres.2023.148289 36813064

[51] Jolly AE, Bălăeţ M, Azor A, et al Detecting axonal injury in individual patients after traumatic brain injury. Brain. 2021;144(1):92–113. 33257929 10.1093/brain/awaa372 PMC7880666

[52] Imms P, Clemente A, Deutscher E, et al Exploring personalized structural connectomics for moderate to severe traumatic brain injury. Network Neurosci. 2023;1(7):160–183. 10.1162/netn_a_00277 PMC10270710 37334004

[53] Theadom A, Hardaker N, Bray C, et al The brain injury screening tool (BIST): Tool development, factor structure and validity. PLoS One. 2021;16(2):e0246512. 33539482 10.1371/journal.pone.0246512 PMC7861451

[54] Gorgolewski KJ, Auer T, Calhoun VD, et al The brain imaging data structure, a format for organizing and describing outputs of neuroimaging experiments. Sci Data. 2016;3:160044. 27326542 10.1038/sdata.2016.44 PMC4978148

[55] Bore A, Guay S, Bedetti C, et al 2023. https://github.com/cbedetti/Dcm2Bids

[56] Li X, Morgan PS, Ashburner J, et al The first step for neuroimaging data analysis: DICOM to NIfTI conversion. J Neurosci Methods. 2016;264:47–56. 26945974 10.1016/j.jneumeth.2016.03.001

[57] Avants BB, Tustison NJ, Song G, et al A reproducible evaluation of ANTs similarity metric performance in brain image registration. NeuroImage. 2011;54(3):2033–2044. 20851191 10.1016/j.neuroimage.2010.09.025 PMC3065962

[58] Tustison NJ, Avants BB, Cook PA, et al N4ITK: Improved N3 bias correction. IEEE Trans Med Imaging. 2010;29(6):1310–1320. 20378467 10.1109/TMI.2010.2046908 PMC3071855

[59] Fischl B . FreeSurfer. NeuroImage. 2012;62(2):774–781. 22248573 10.1016/j.neuroimage.2012.01.021 PMC3685476

[60] Merenstein JL, Zhao J, Overson DK, et al Depth- and curvature-based quantitative susceptibility mapping analyses of cortical iron in Alzheimer’s disease. Cereb Cortex. 2024;34(2):bhad525. 38185996 10.1093/cercor/bhad525 PMC10839848

[61] Dymerska B, Eckstein K, Bachrata B, et al Phase unwrapping with a rapid open-source minimum spanning tree algorithm (ROMEO). Magn Reson Med. 2020;85(4):2294–2308. 33104278 10.1002/mrm.28563 PMC7821134

[62] Liu T, Khalidov I, de Rochefort L, et al A novel background field removal method for MRI using projection onto dipole fields (PDF). NMR Biomed. 2011;24(9):1129–1136. 21387445 10.1002/nbm.1670 PMC3628923

[63] Kames C, Wiggermann V, Rauscher A. Rapid two-step dipole inversion for susceptibility mapping with sparsity priors. NeuroImage. 2018;167:276–283. 29138089 10.1016/j.neuroimage.2017.11.018

[64] Bilgic B, Costagli M, Chan KS, et al Recommended implementation of quantitative susceptibility mapping for clinical research in the brain: A consensus of the ISMRM Electro-Magnetic Tissue Properties Study Group. Magn Reson Med. 2024;91(5):1834–1862. 38247051 10.1002/mrm.30006 PMC10950544

[65] Straub S, Schneider TM, Emmerich J, et al Suitable reference tissues for quantitative susceptibility mapping of the brain. Magn Reson Med. 2017;78(1):204–214. 27529579 10.1002/mrm.26369

[66] Stewart AW, Bollman S. 2022. https://qsmxt.github.io/QSMxT/

[67] Stewart AW, Robinson SD, O’Brien K, et al QSMxT: Robust masking and artifact reduction for quantitative susceptibility mapping. Magn Reson Med. 2022;87(3):1289–1300. 34687073 10.1002/mrm.29048 PMC7612305

[68] Jenkinson M, Beckmann CF, Behrens TE, et al FSL. NeuroImage. 2012;62(2):782–790. 21979382 10.1016/j.neuroimage.2011.09.015

[69] Smith SM, Jenkinson M, Woolrich MW, et al Advances in functional and structural MR image analysis and implementation as FSL. NeuroImage. 2004;23(Suppl 1):S208–S219. 15501092 10.1016/j.neuroimage.2004.07.051

[70] Woolrich MW, Jbabdi S, Patenaude B, et al Bayesian analysis of neuroimaging data in FSL. NeuroImage. 2009;45(Suppl 1):S173–S185. 19059349 10.1016/j.neuroimage.2008.10.055

[71] Smith SM . Fast robust automated brain extraction. Hum Brain Mapp. 2002;17(3):143–155. 12391568 10.1002/hbm.10062 PMC6871816

[72] Greve DN, Fischl B. Accurate and robust brain image alignment using boundary-based registration. NeuroImage. 2009;48(1):63–72. 19573611 10.1016/j.neuroimage.2009.06.060 PMC2733527

[73] Jenkinson M, Bannister P, Brady M, et al Improved optimization for the robust and accurate linear registration and motion correction of brain images. NeuroImage. 2002;17(2):825–841. 12377157 10.1016/s1053-8119(02)91132-8

[74] Jenkinson M, Smith S. A global optimization method for robust affine registration of brain images. Med Image Anal. 2001;5(2):143–156. 11516708 10.1016/s1361-8415(01)00036-6

[75] Reichenbach JR . The future of susceptibility contrast for assessment of anatomy and function. NeuroImage. 2012;62(2):1311–1315. 22245644 10.1016/j.neuroimage.2012.01.004

[76] Ma Y, Bruce IP, Yeh CH, et al Column-based cortical depth analysis of the diffusion anisotropy and radiality in submillimeter whole-brain diffusion tensor imaging of the human cortical gray matter in vivo. NeuroImage. 2023;270:119993. 36863550 10.1016/j.neuroimage.2023.119993 PMC10037338

[77] Daducci A, Gerhard S, Griffa A, et al The connectome mapper: An open-source processing pipeline to map connectomes with MRI. PLoS One. 2012;7(12):e48121. 23272041 10.1371/journal.pone.0048121 PMC3525592

[78] Desikan RS, Segonne F, Fischl B, et al An automated labeling system for subdividing the human cerebral cortex on MRI scans into gyral based regions of interest. NeuroImage. 2006;31(3):968–980. 16530430 10.1016/j.neuroimage.2006.01.021

[79] Tournier DJ, Smith R, Raffelt D, et al MRtrix3: A fast, flexible and open software framework for medical image processing and visualisation. NeuroImage. 2019;202:116137. 31473352 10.1016/j.neuroimage.2019.116137

[80] Waehnert MD, Dinse J, Weiss M, et al Anatomically motivated modeling of cortical laminae. NeuroImage. 2014;93(Pt 2):210–220. 23603284 10.1016/j.neuroimage.2013.03.078

[81] Waehnert MD, Dinse J, Schäfer A, et al A subject-specific framework for in vivo myeloarchitectonic analysis using high resolution quantitative MRI. NeuroImage. 2016;125:94–107. 26455795 10.1016/j.neuroimage.2015.10.001

[82] Pienaar R, Rojczyk P, Seitz-Holland J, et al A methodology for analyzing curvature in the developing brain from preterm to adult. Int J Imaging Syst Technol. 2008;18(1):42–68. 19936261 10.1002/ima.v18:1 PMC2779548

[83] Fischl B, Dale AM. Measuring the thickness of the human cerebral cortex from magnetic resonance images. Proc Natl Acad Sci U S A. 2000;97(20):11050–11055. 10984517 10.1073/pnas.200033797 PMC27146

[84] Tukey JW . Exploratory data analysis. Addison-Wesley Publishing Company; 1977.

[85] Benjamini Y, Hochberg Y. Controlling the false discovery rate: A practical and powerful approach to multiple testing. J R Stat Soc Ser B (Methodol). 1995;57(1):289–300.

[86] Kroshus E, Garnett B, Hawrilenko M, et al Concussion under-reporting and pressure from coaches, teammates, fans, and parents. Soc Sci Med. 2015;134:66–75. 25917137 10.1016/j.socscimed.2015.04.011 PMC4651185

[87] McCrea M, Meier T, Huber D, et al Role of advanced neuroimaging, fluid biomarkers and genetic testing in the assessment of sport-related concussion: A systematic review. Br J Sports Med. 2017;51(12):919–929. 28455364 10.1136/bjsports-2016-097447

[88] Shenton ME, Hamoda HM, Schneiderman JS, et al A review of magnetic resonance imaging and diffusion tensor imaging findings in mild traumatic brain injury. Brain Imaging Behav. 2012;6(2):137–192. 22438191 10.1007/s11682-012-9156-5 PMC3803157

[89] Ma H, Dong Y, Chu Y, et al The mechanisms of ferroptosis and its role in Alzheimer’s disease. Front Mol Biosci. 2022;9:965064. 36090039 10.3389/fmolb.2022.965064 PMC9459389

[90] Ghadery C, Pirpamer L, Hofer E, et al R2* mapping for brain iron: Associations with cognition in normal aging. Neurobiol Aging. 2015;36(2):925–932. 25443291 10.1016/j.neurobiolaging.2014.09.013

[91] Stankiewicz J, Panter SS, Neema M, et al Iron in chronic brain disorders: Imaging and neurotherapeutic implications. Neurotherapeutics. 2007;4(3):371–386. 17599703 10.1016/j.nurt.2007.05.006 PMC1963417

[92] Wills AJ, Sawle GV, Guilbert PR, et al Palatal tremor and cognitive decline in neuroferritinopathy. J Neurol Neurosurg Psychiatry. 2002;73:91–92. 12082064 10.1136/jnnp.73.1.91 PMC1757327

[93] McKee AC, Daneshvar DH. The neuropathology of traumatic brain injury. In: Connolly ES Jr, ed. Handbook of clinical neurology. Elsevier B.V.; 2015:45–66. 10.1016/B978-0-444-52892-6.00004-0 PMC4694720 25702209

[94] Murray HC, Osterman C, Bell P, et al Neuropathology in chronic traumatic encephalopathy: A systematic review of comparative post-mortem histology literature. Acta Neuropathol Commun. 2022;10:109. 35933388 10.1186/s40478-022-01413-9 PMC9356428

[95] McKee AC, Stein TD, Huber BR, et al Chronic traumatic encephalopathy (CTE): Criteria for neuropathological diagnosis and relationship to repetitive head impacts. Acta Neuropathol. 2023;145(4):371–394. 36759368 10.1007/s00401-023-02540-w PMC10020327

[96] Bigler ED, Maxwell WL. Neuropathology of mild traumatic brain injury: Relationship to neuroimaging findings. Brain Imaging Behav. 2012;6(2):108–136. 22434552 10.1007/s11682-011-9145-0

[97] Basil RA, Westwater ML, Wiener M, et al A causal role of the right superior temporal sulcus in emotion recognition from biological motion. Open Mind (Camb). 2017;2(1):26–36.

[98] Beauchamp MS . The social mysteries of the superior temporal sulcus. Trends Cogn Sci. 2015;19(9):489–490. 26208834 10.1016/j.tics.2015.07.002 PMC4556565

[99] Deen B, Koldewyn K, Kanwisher N, et al Functional organization of social perception and cognition in the superior temporal sulcus. Cereb Cortex. 2015;25(11):4596–4609. 26048954 10.1093/cercor/bhv111 PMC4816802

[100] Leaver AM, Renier L, Chevillet MA, et al Dysregulation of limbic and auditory networks in tinnitus. Neuron. 2011;69(1):33–43. 21220097 10.1016/j.neuron.2010.12.002 PMC3092532

[101] Hein G, Knight RT. Superior temporal sulcus - It’s my area: Or is it? J Cogn Neurosci. 2008;20(12):2125–2136. 18457502 10.1162/jocn.2008.20148

[102] Dieterich M, Brandt T. Functional brain imaging of peripheral and central vestibular disorders. Brain. 2008;131(10):2538–2552. 18515323 10.1093/brain/awn042

[103] Boulloche N, Denuelle M, Payoux P, et al Photophobia in migraine: An interictal PET study of cortical hyperexcitability and its modulation by pain. J Neurol Neurosurg Psychiatry. 2010;81(9):978–984. 20595138 10.1136/jnnp.2009.190223

[104] Levin HS, Amparo E, Eisenberg HM, et al Magnetic resonance imaging and computerized tomography in relation to the neurobehaviorial sequelae of mild and moderate head injuries. J Neurosurg. 1987;66(5):706–713. 3572497 10.3171/jns.1987.66.5.0706

[105] Kornguth S, Rutledge N, Perlaza G, et al A proposed mechanism for development of CTE following concussive events: Head impact, water hammer injury, neurofilament release, and autoimmune processes. Brain Sci. 2017;7(12):164. 29257064 10.3390/brainsci7120164 PMC5742767

[106] King JB, Lopez-Larson MP, Yurgelun-Todd DA. Mean cortical curvature reflects cytoarchitecture restructuring in mild traumatic brain injury. NeuroImage Clin. 2016;11:81–89. 26909332 10.1016/j.nicl.2016.01.003 PMC4735656

[107] Ghajari M, Hellyer PJ, Sharp DJ. Computational modelling of traumatic brain injury predicts the location of chronic traumatic encephalopathy pathology. Brain. 2017;140(2):333–343. 28043957 10.1093/brain/aww317 PMC5278309

[108] Fukunaga M, Li TQ, Van Gelderen P, et al Layer-specific variation of iron content in cerebral cortex as a source of MRI contrast. Proc Natl Acad Sci U S A. 2010;107(8):3834–3839. 20133720 10.1073/pnas.0911177107 PMC2840419

[109] Shin HG, Lee J, Yun YH, et al χ-separation: Magnetic susceptibility source separation toward iron and myelin mapping in the brain. NeuroImage. 2021;240:118371. 34242783 10.1016/j.neuroimage.2021.118371

[110] Miyashita Y . Operating principles of the cerebral cortex as a six-layered network in primates: Beyond the classic canonical circuit model. Proc Japan Acad B. 2022;98(3):93–111. 35283409 10.2183/pjab.98.007 PMC8948418

[111] Pankatz L, Rojczyk P, Seitz-Holland J, et al Adverse outcome following mild traumatic brain injury is associated with microstructure alterations at the gray and white matter boundary. J Clin Med. 2023;12(16):5415. 37629457 10.3390/jcm12165415 PMC10455493

[112] Liu J, Kou ZF, Tian YQ. Diffuse axonal injury after traumatic cerebral microbleeds: An evaluation of imaging techniques. Brain Inj. 2014;9(12):1222–1230. 10.4103/1673-5374.135330 PMC4146289 25206786

[113] McKee AC, Stein TD, Nowinski CJ, et al The spectrum of disease in chronic traumatic encephalopathy. Brain. 2013;136(Pt 1):43–64. 23208308 10.1093/brain/aws307 PMC3624697

[114] Sandsmark DK, Bashir A, Wellington CL, et al Cerebral microvascular injury: A potentially treatable endophenotype of traumatic brain injury-induced neurodegeneration. Neuron. 2019;103(3):367–379. 31394062 10.1016/j.neuron.2019.06.002 PMC6688649

[115] Wu Y, Wu H, Guo X, et al Blood–brain barrier dysfunction in mild traumatic brain injury: Evidence from preclinical murine models. Front Physiol. 2020;11:1030. 32973558 10.3389/fphys.2020.01030 PMC7472692

[116] Ward RJ, Zucca FA, Duyn JH, et al The role of iron in brain ageing and neurodegenerative disorders. Lancet Neurol. 2014;13(10):1045–1060. 25231526 10.1016/S1474-4422(14)70117-6 PMC5672917

[117] Tohka J . Partial volume effect modeling for segmentation and tissue classification of brain magnetic resonance images: A review. World J Radiol. 2014;6(11):855–864. 25431640 10.4329/wjr.v6.i11.855 PMC4241492

[118] Sui YV, Masurkar AV, Rusinek H, et al Cortical myelin profile variations in healthy aging brain: A T1w/T2w ratio study. NeuroImage. 2022;264:119743. 36368498 10.1016/j.neuroimage.2022.119743 PMC9904172

[119] Northall A, Doehler J, Weber M, et al Layer-specific vulnerability is a mechanism of topographic map aging. Neurobiol Aging. 2023;128:17–32. 37141729 10.1016/j.neurobiolaging.2023.04.002

[120] Aggarwal M, Nauen DW, Troncoso JC, et al Probing region-specific microstructure of human cortical areas using high angular and spatial resolution diffusion MRI. NeuroImage. 2015;105:198–207. 25449747 10.1016/j.neuroimage.2014.10.053 PMC4262592

[121] Hallgren B, Sourander P. The effect of age on the non-haemin iron in the human brain. J Neurochem. 1958;3(1):41–51. 13611557 10.1111/j.1471-4159.1958.tb12607.x

[122] Marquand AF, Rezek I, Buitelaar J, et al Understanding heterogeneity in clinical cohorts using normative models: Beyond case-control studies. Biol Psychiatry. 2016;80(7):552–561. 26927419 10.1016/j.biopsych.2015.12.023 PMC5023321

[123] Wunderle K, Hoeger KM, Wasserman E, et al Menstrual phase as predictor of outcome after mild traumatic brain injury in women. J Head Trauma Rehabil. 2014;29(5):E1–E8. 10.1097/HTR.0000000000000006 PMC5237582 24220566

[124] Gallagher V, Kramer N, Abbott K, et al The effects of sex differences and hormonal contraception on outcomes after collegiate sports-related concussion. J Neurotrauma. 2018;35(11):1242–1247. 29336208 10.1089/neu.2017.5453 PMC10331147

[125] Tierney RT, Sitler MR, Swanik CB, et al Gender differences in head-neck segment dynamic stabilization during head acceleration. Med Sci Sports Exerc. 2005;37(2):272–279. 15692324 10.1249/01.mss.0000152734.47516.aa

[126] Lee J, Ji S, Oh SH. So you want to image myelin using MRI: Magnetic susceptibility source separation for myelin imaging. Magn Reson Med Sci. 2024;23(3):291–306. 38644201 10.2463/mrms.rev.2024-0001 PMC11234950

[127] Li Z, Feng R, Liu Q, et al APART-QSM: An improved sub-voxel quantitative susceptibility mapping for susceptibility source separation using an iterative data fitting method. NeuroImage. 2023;274:120148. 37127191 10.1016/j.neuroimage.2023.120148

