# Supplementary material for: Mild traumatic brain injury increases cortical iron: evidence from individual susceptibility mapping
Source: Brain Commun. 2025 Mar 12;7(2):fcaf110. doi: 10.1093/braincomms/fcaf110 (PMC11954555; doi:10.1093/braincomms/fcaf110)

Supplementary Figure 1

Comparative graphs illustrate mean susceptibility values averaged across all participants ($N = 60$) when sampling at six (left) versus three (right) cortical depths for each curvature bin (crown = blue; bank = orange; fundus = yellow) for the frontal pole. While sampling at six depths may introduce some redundancy into the model, the overall susceptibility patterns remain similar. Restricting the analysis to three depths appears to risk overlooking subtle yet informative differences that only emerge at finer depth granularity. Susceptibility is measured in parts per million (ppm).

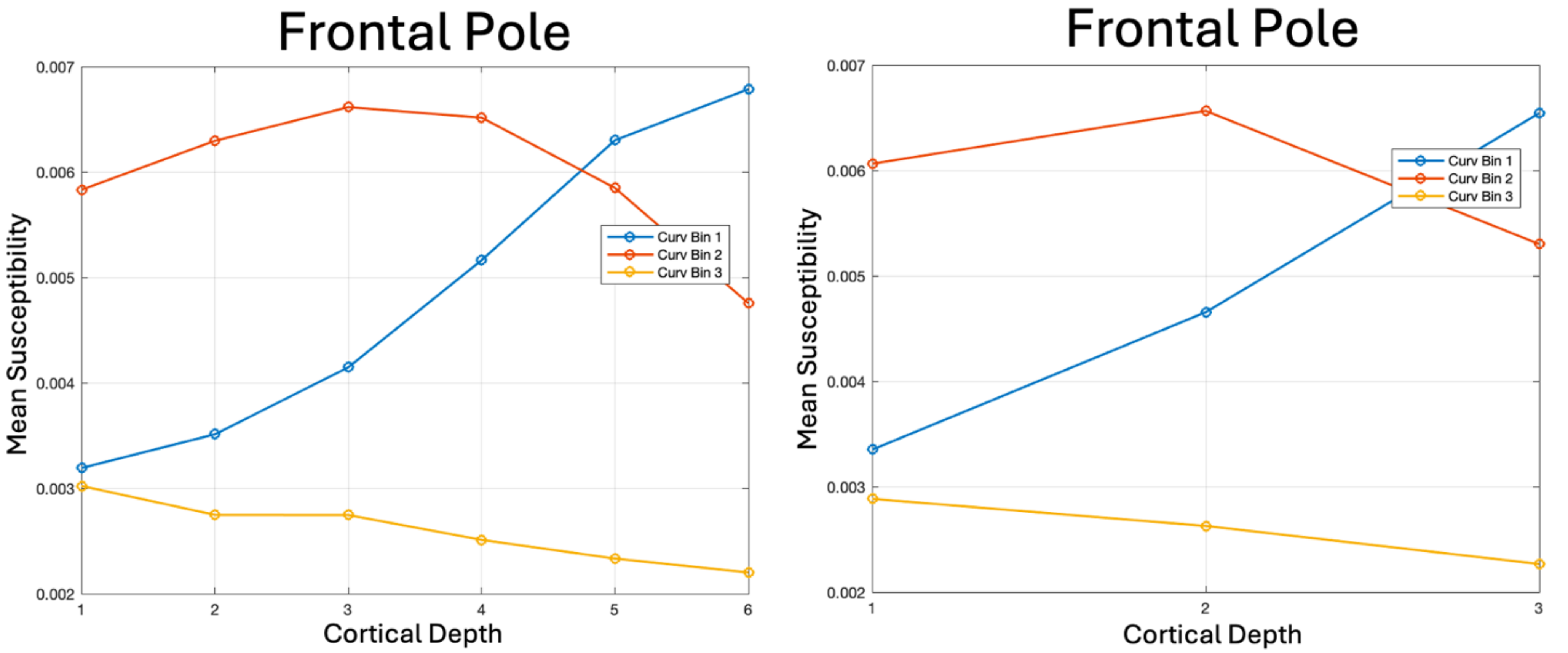

Supplementary Figure 2

Comparative graphs illustrate mean susceptibility values averaged across all participants (*N* = 60) when sampling at six (left) versus three (right) cortical depths for each curvature bin (crown = blue; bank = orange; fundus = yellow) for the medial orbitofrontal cortex. While sampling at six depths may introduce some redundancy into the model, the overall susceptibility patterns remain similar. Restricting the analysis to three depths appears to risk overlooking subtle yet informative differences that only emerge at finer depth granularity. Susceptibility is measured in parts per million (ppm).

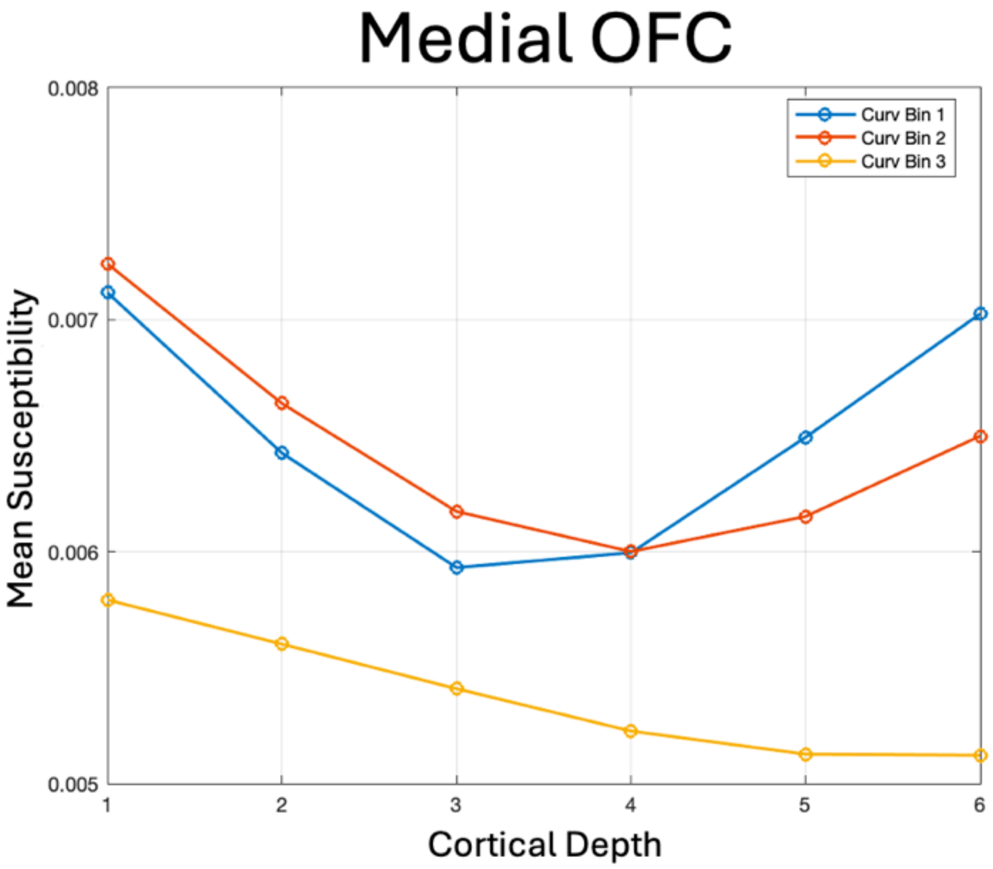

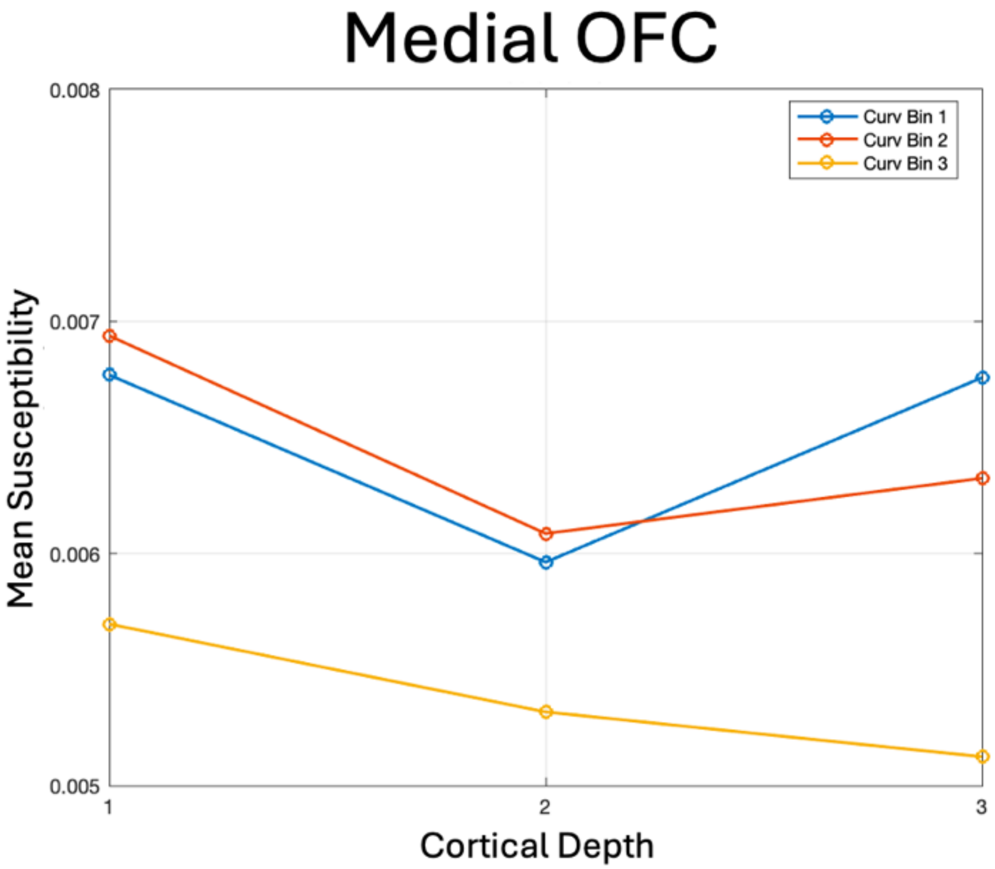

Supplementary Figure 3

Comparative graphs illustrate mean susceptibility values averaged across all participants ($N = 60$) when sampling at six (left) versus three (right) cortical depths for each curvature bin (crown = blue; bank = orange; fundus = yellow) for the lateral orbitofrontal cortex. While sampling at six depths may introduce some redundancy into the model, the overall susceptibility patterns remain similar. Restricting the analysis to three depths appears to risk overlooking subtle yet informative differences that only emerge at finer depth granularity. Susceptibility is measured in parts per million (ppm).

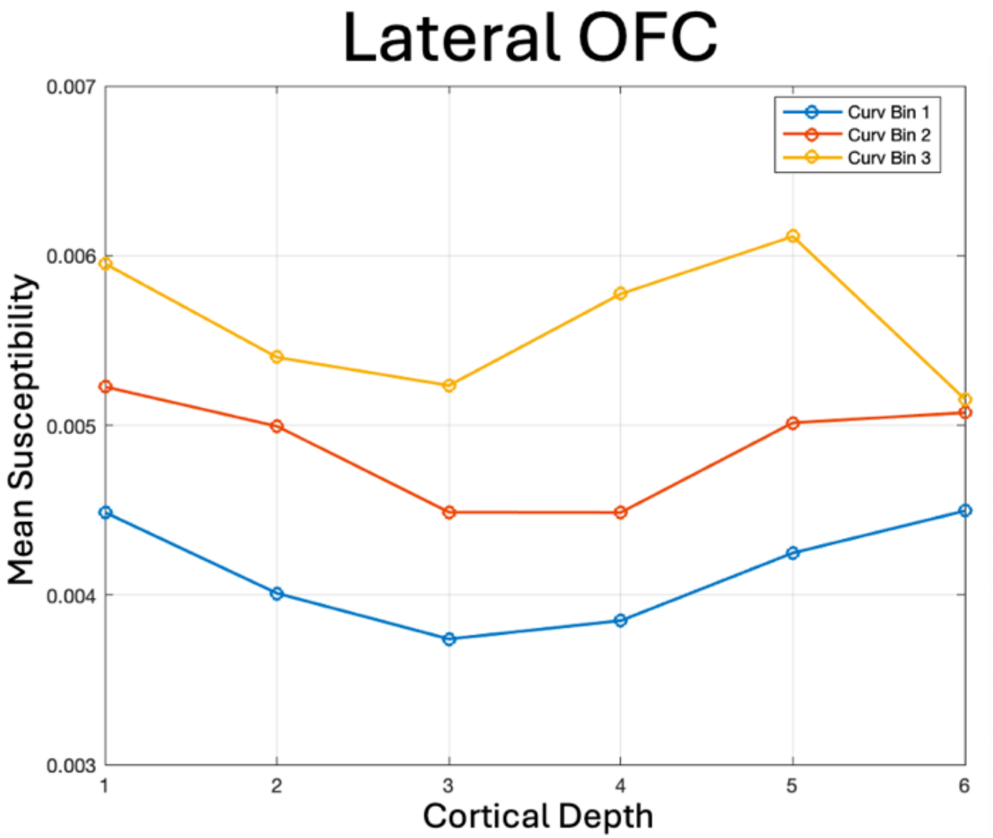

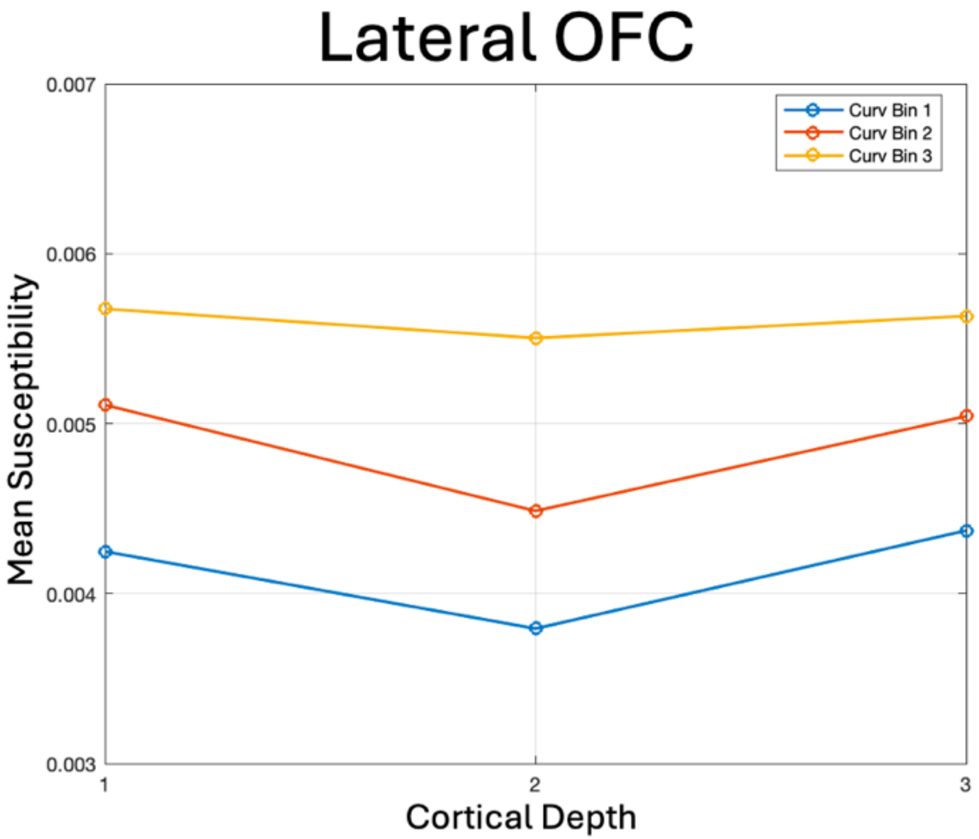

Supplementary Figure 4

Comparative graphs illustrate mean susceptibility values averaged across all participants (*N* = 60) when sampling at six (left) versus three (right) cortical depths for each curvature bin (crown = blue; bank = orange; fundus = yellow) for the pars orbitalis. While sampling at six depths may introduce some redundancy into the model, the overall susceptibility patterns remain similar. Restricting the analysis to three depths appears to risk overlooking subtle yet informative differences that only emerge at finer depth granularity. Susceptibility is measured in parts per million (ppm).

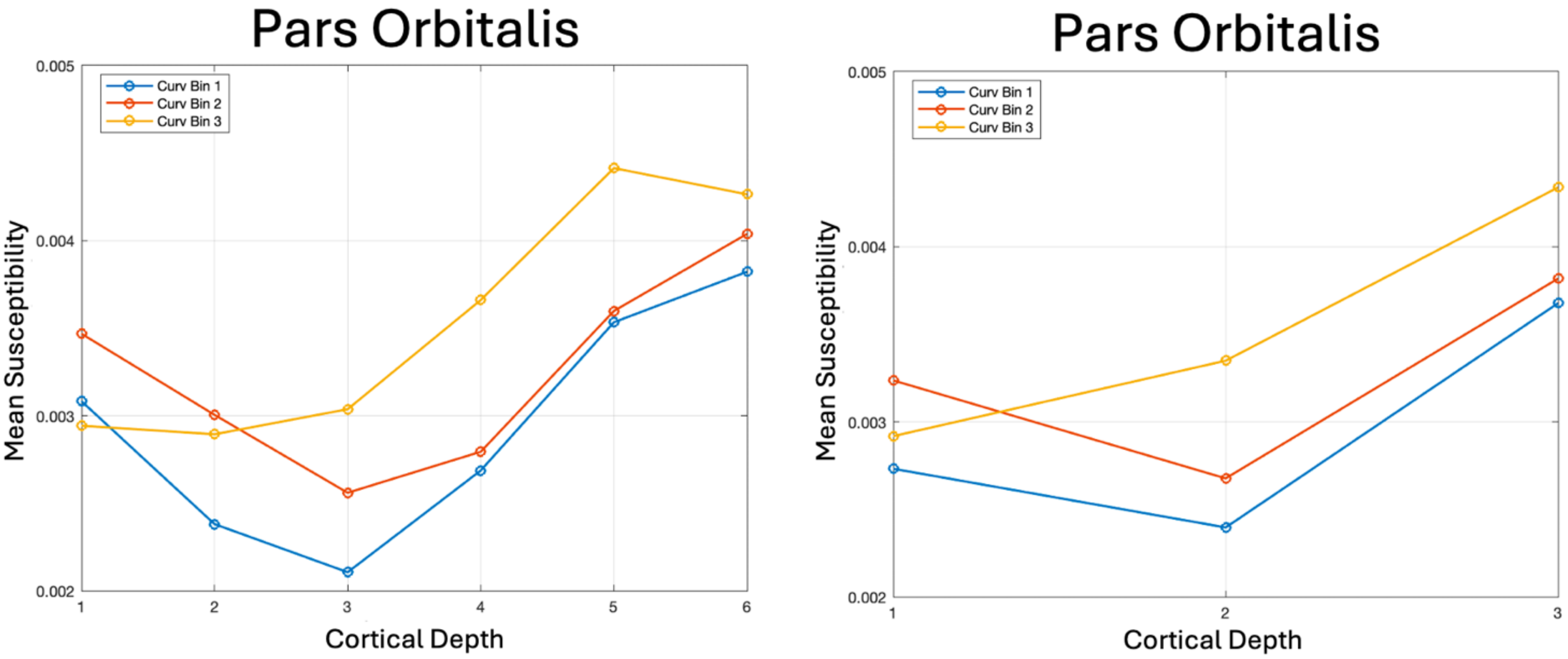

Supplementary Figure 5

Comparative graphs illustrate mean susceptibility values averaged across all participants (*N* = 60) when sampling at six (left) versus three (right) cortical depths for each curvature bin (crown = blue; bank = orange; fundus = yellow) for the rostral medial prefrontal cortex. While sampling at six depths may introduce some redundancy into the model, the overall susceptibility patterns remain similar. Restricting the analysis to three depths appears to risk overlooking subtle yet informative differences that only emerge at finer depth granularity. Susceptibility is measured in parts per million (ppm).

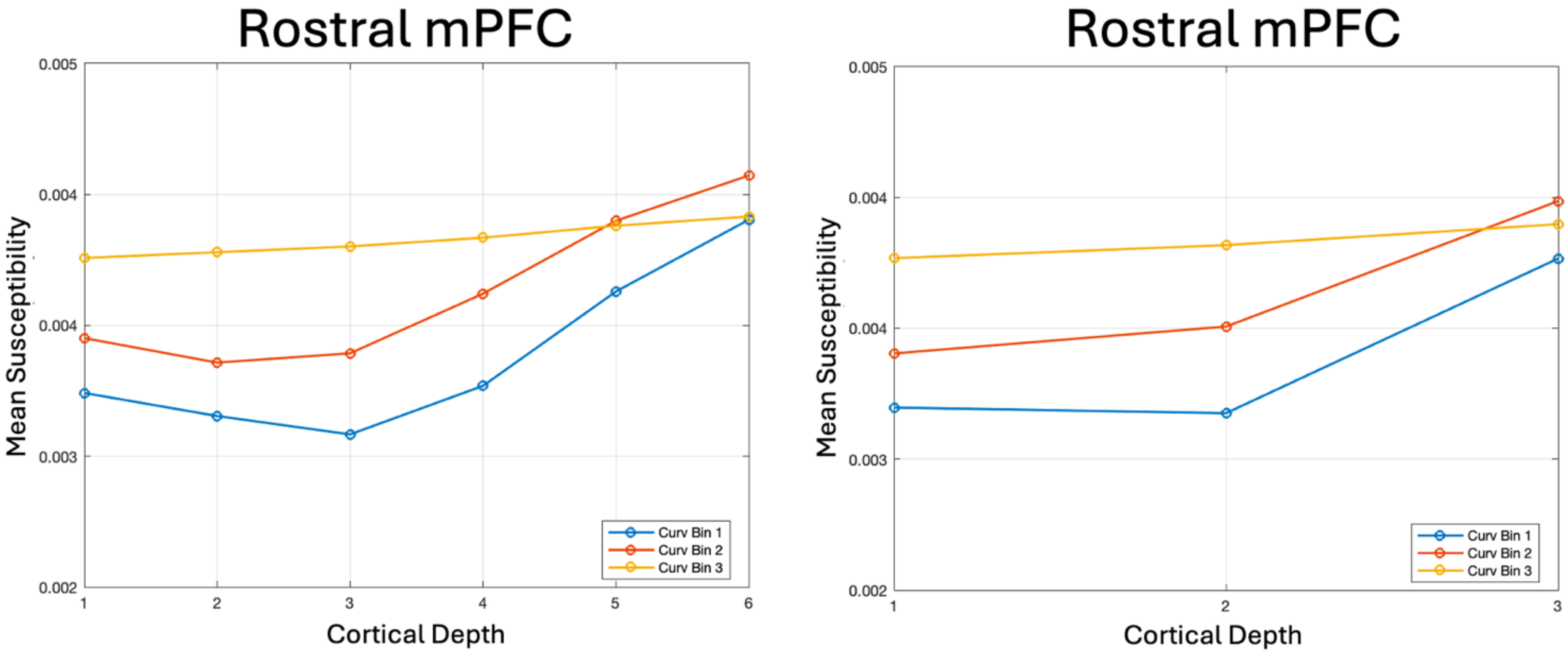

Supplementary Figure 6

Comparative graphs illustrate mean susceptibility values averaged across all participants ($N = 60$) when sampling at six (left) versus three (right) cortical depths for each curvature bin (crown = blue; bank = orange; fundus = yellow) for the caudal medial prefrontal cortex. While sampling at six depths may introduce some redundancy into the model, the overall susceptibility patterns remain similar. Restricting the analysis to three depths appears to risk overlooking subtle yet informative differences that only emerge at finer depth granularity. Susceptibility is measured in parts per million (ppm).

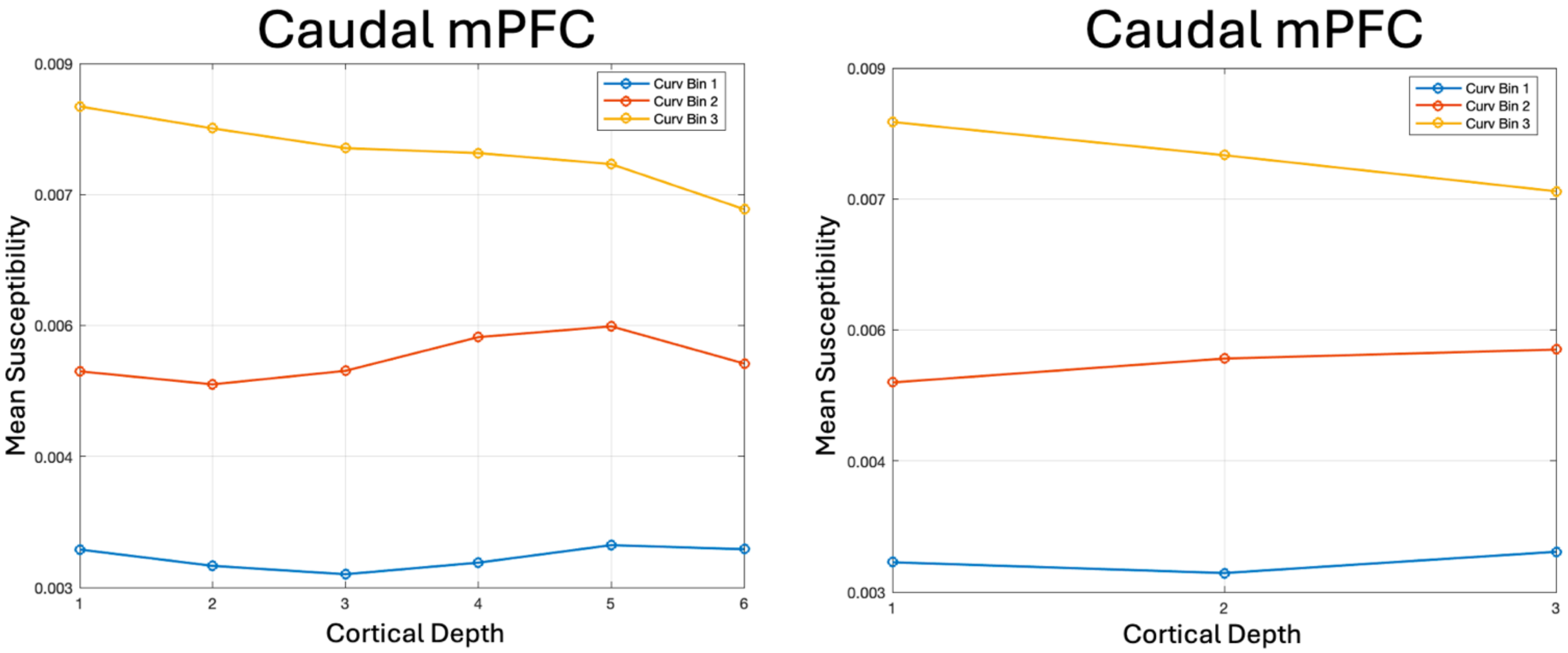

Supplementary Figure 7

Comparative graphs illustrate mean susceptibility values averaged across all participants (*N* = 60) when sampling at six (left) versus three (right) cortical depths for each curvature bin (crown = blue; bank = orange; fundus = yellow) for the pars opercularis. While sampling at six depths may introduce some redundancy into the model, the overall susceptibility patterns remain similar. Restricting the analysis to three depths appears to risk overlooking subtle yet informative differences that only emerge at finer depth granularity. Susceptibility is measured in parts per million (ppm).

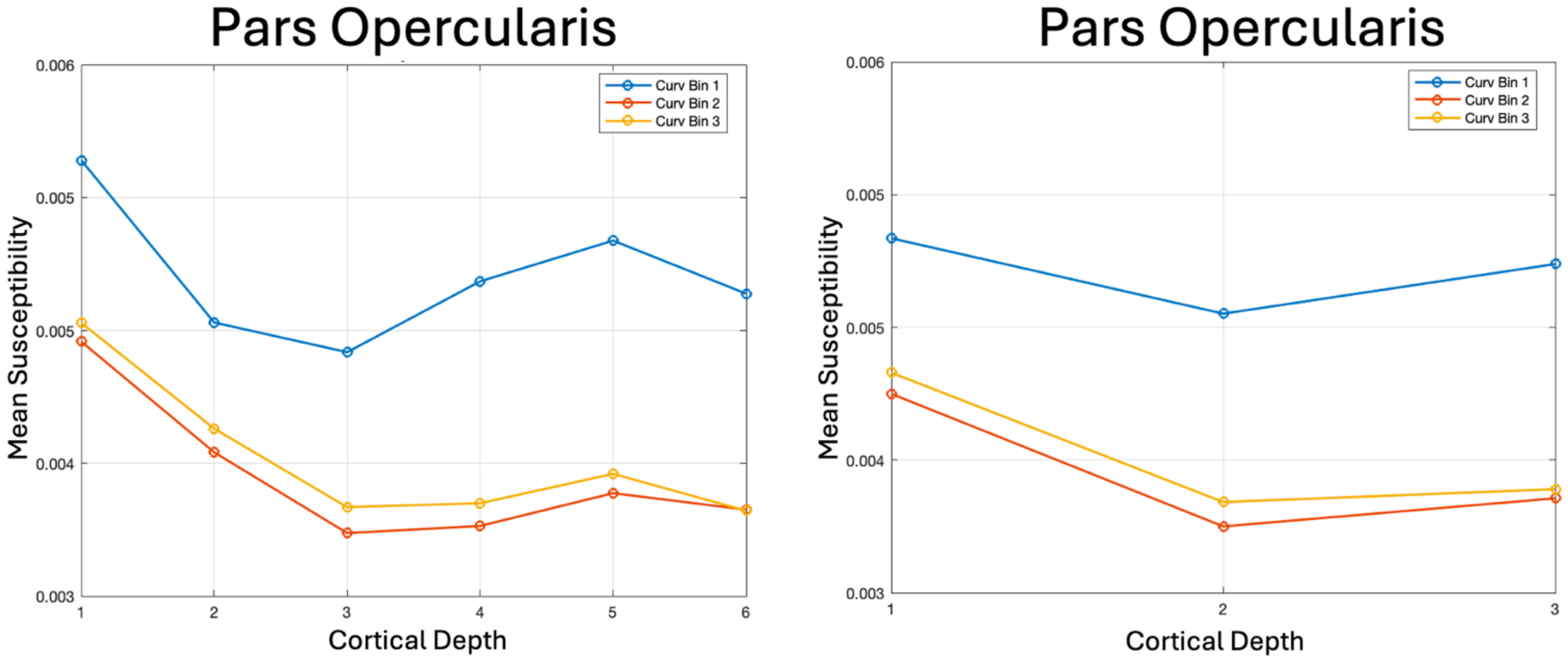

Supplementary Figure 8

Comparative graphs illustrate mean susceptibility values averaged across all participants ($N = 60$) when sampling at six (left) versus three (right) cortical depths for each curvature bin (crown = blue; bank = orange; fundus = yellow) for the superior frontal gyrus. While sampling at six depths may introduce some redundancy into the model, the overall susceptibility patterns remain similar. Restricting the analysis to three depths appears to risk overlooking subtle yet informative differences that only emerge at finer depth granularity. Susceptibility is measured in parts per million (ppm).

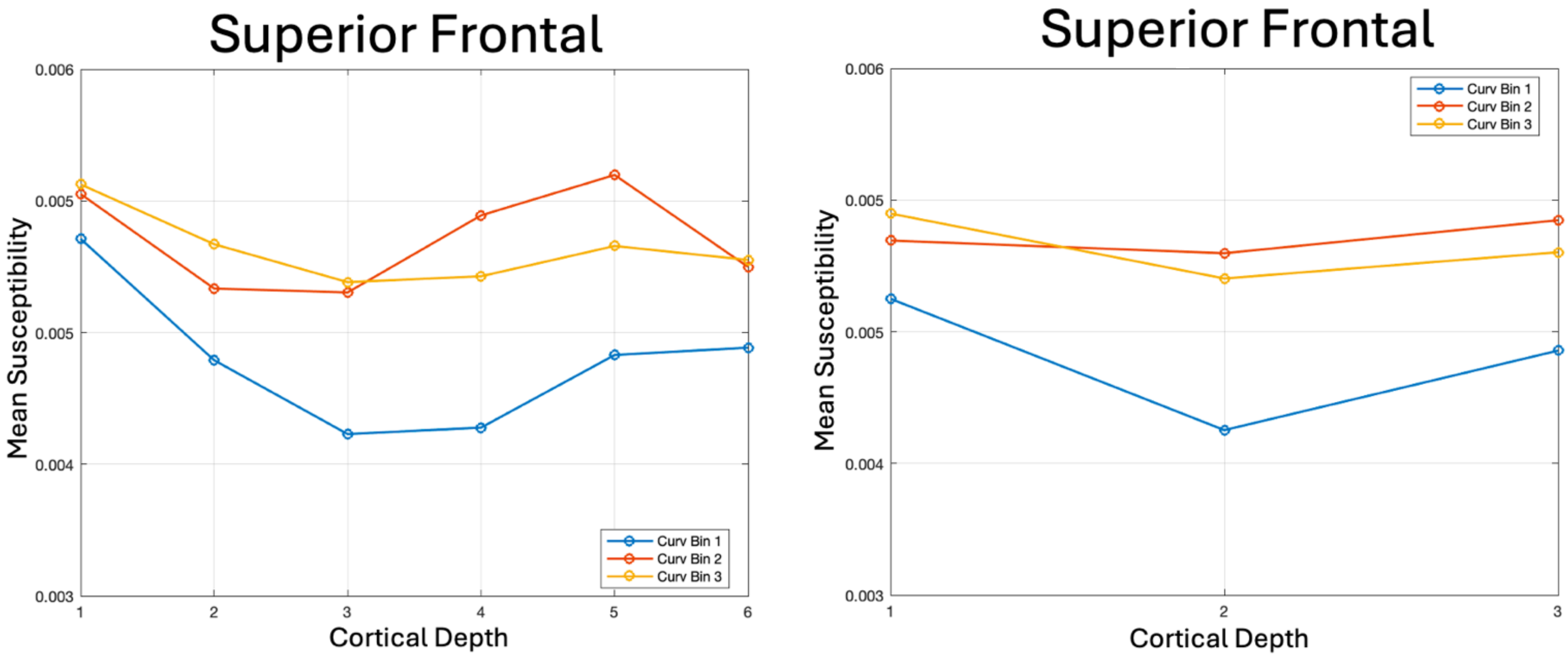

Supplementary Figure 9

Comparative graphs illustrate mean susceptibility values averaged across all participants (*N* = 60) when sampling at six (left) versus three (right) cortical depths for each curvature bin (crown = blue; bank = orange; fundus = yellow) for the pars triangularis. While sampling at six depths may introduce some redundancy into the model, the overall susceptibility patterns remain similar. Restricting the analysis to three depths appears to risk overlooking subtle yet informative differences that only emerge at finer depth granularity. Susceptibility is measured in parts per million (ppm).

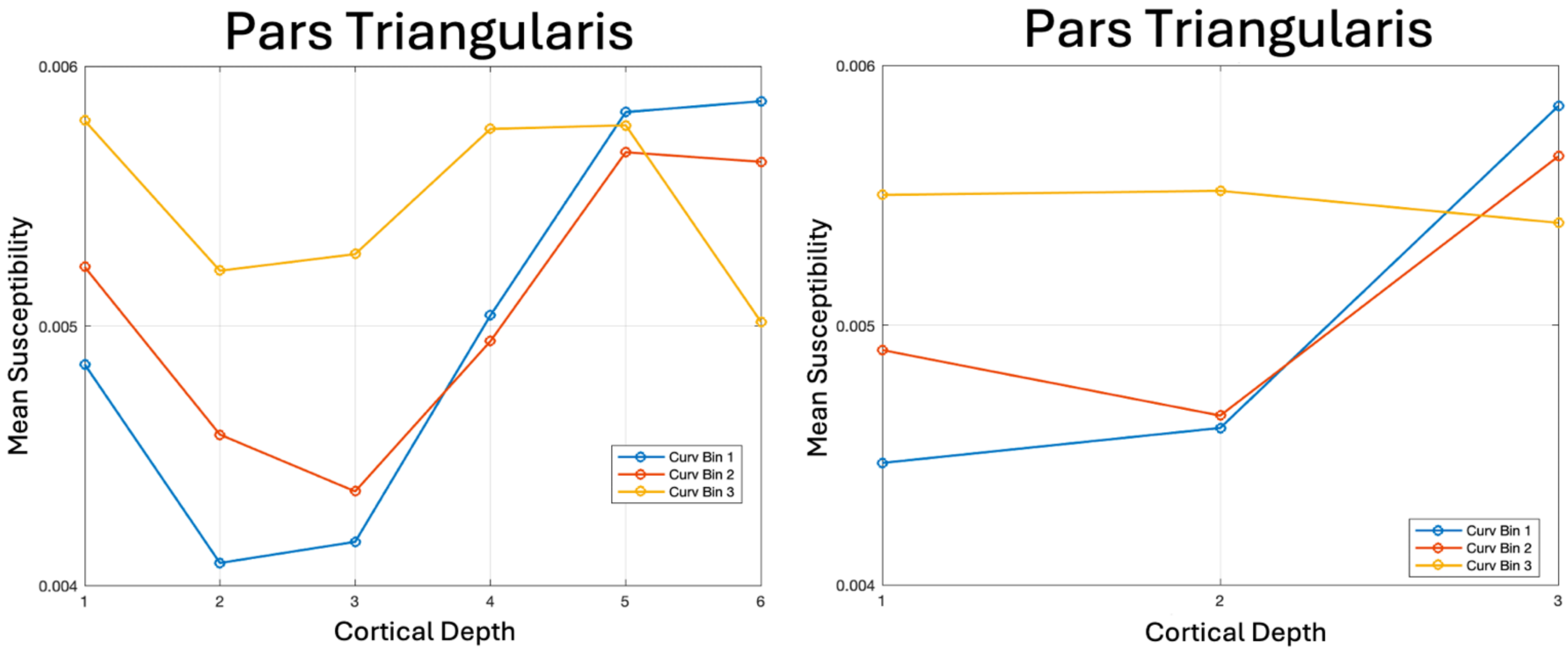

Supplementary Figure 10

Comparative graphs illustrate mean susceptibility values averaged across all participants ($N = 60$) when sampling at six (left) versus three (right) cortical depths for each curvature bin (crown = blue; bank = orange; fundus = yellow) for the precentral gyrus. While sampling at six depths may introduce some redundancy into the model, the overall susceptibility patterns remain similar. Restricting the analysis to three depths appears to risk overlooking subtle yet informative differences that only emerge at finer depth granularity. Susceptibility is measured in parts per million (ppm).

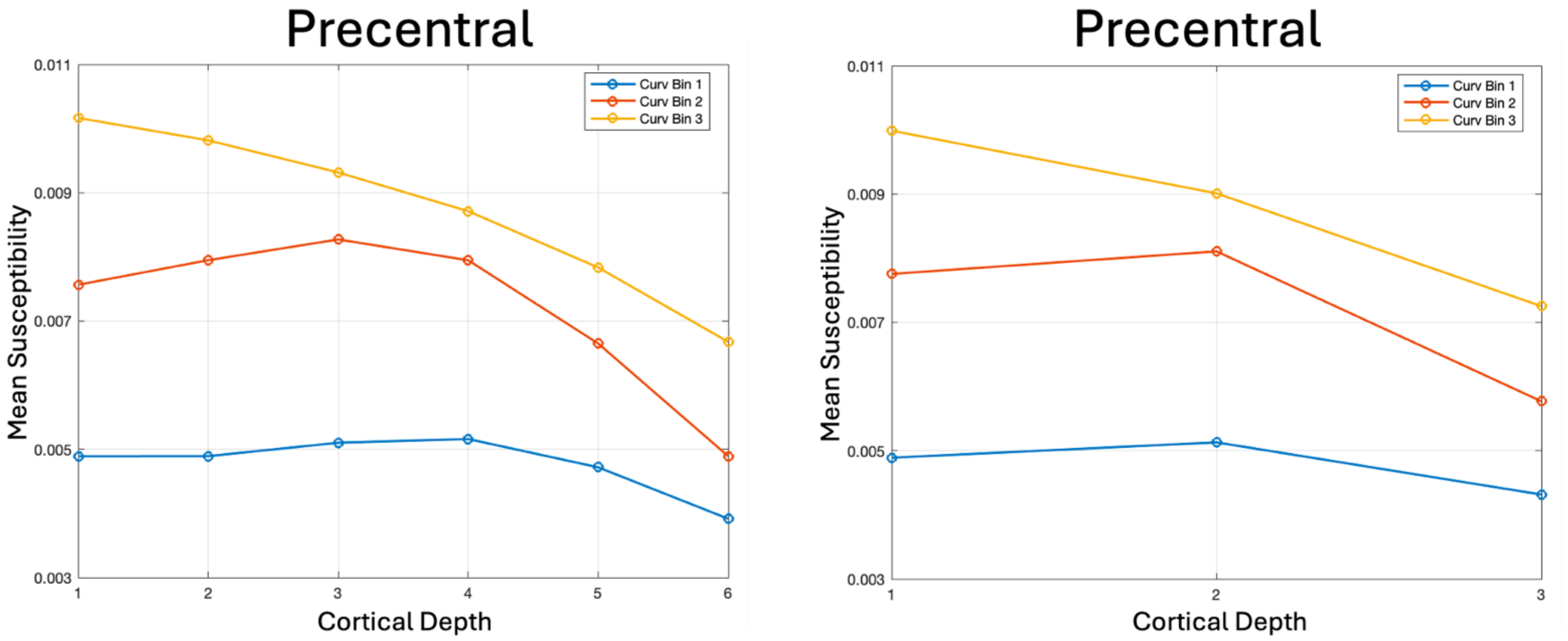

Supplementary Figure 11

Comparative graphs illustrate mean susceptibility values averaged across all participants (*N* = 60) when sampling at six (left) versus three (right) cortical depths for each curvature bin (crown = blue; bank = orange; fundus = yellow) for the superior parietal lobule. While sampling at six depths may introduce some redundancy into the model, the overall susceptibility patterns remain similar. Restricting the analysis to three depths appears to risk overlooking subtle yet informative differences that only emerge at finer depth granularity. Susceptibility is measured in parts per million (ppm).

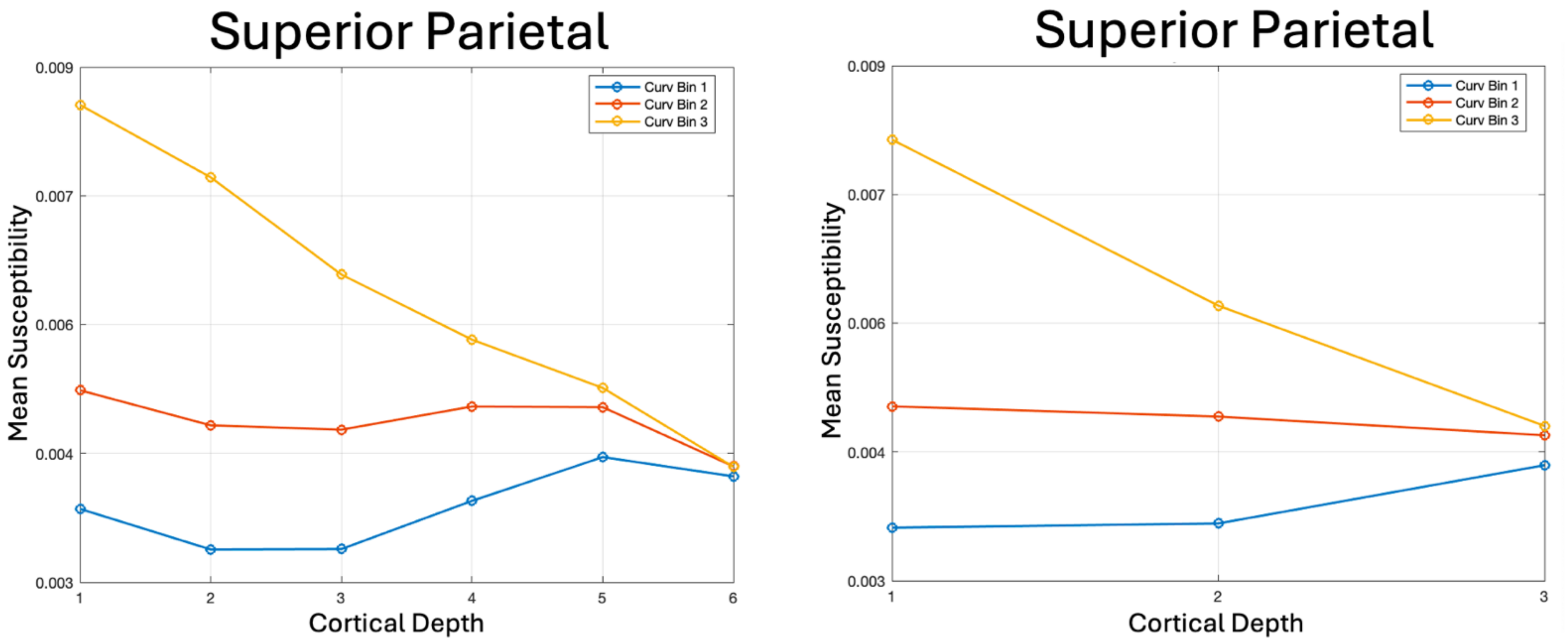

Supplementary Figure 12

Comparative graphs illustrate mean susceptibility values averaged across all participants ($N = 60$) when sampling at six (left) versus three (right) cortical depths for each curvature bin (crown = blue; bank = orange; fundus = yellow) for the inferior parietal lobule. While sampling at six depths may introduce some redundancy into the model, the overall susceptibility patterns remain similar. Restricting the analysis to three depths appears to risk overlooking subtle yet informative differences that only emerge at finer depth granularity. Susceptibility is measured in parts per million (ppm).

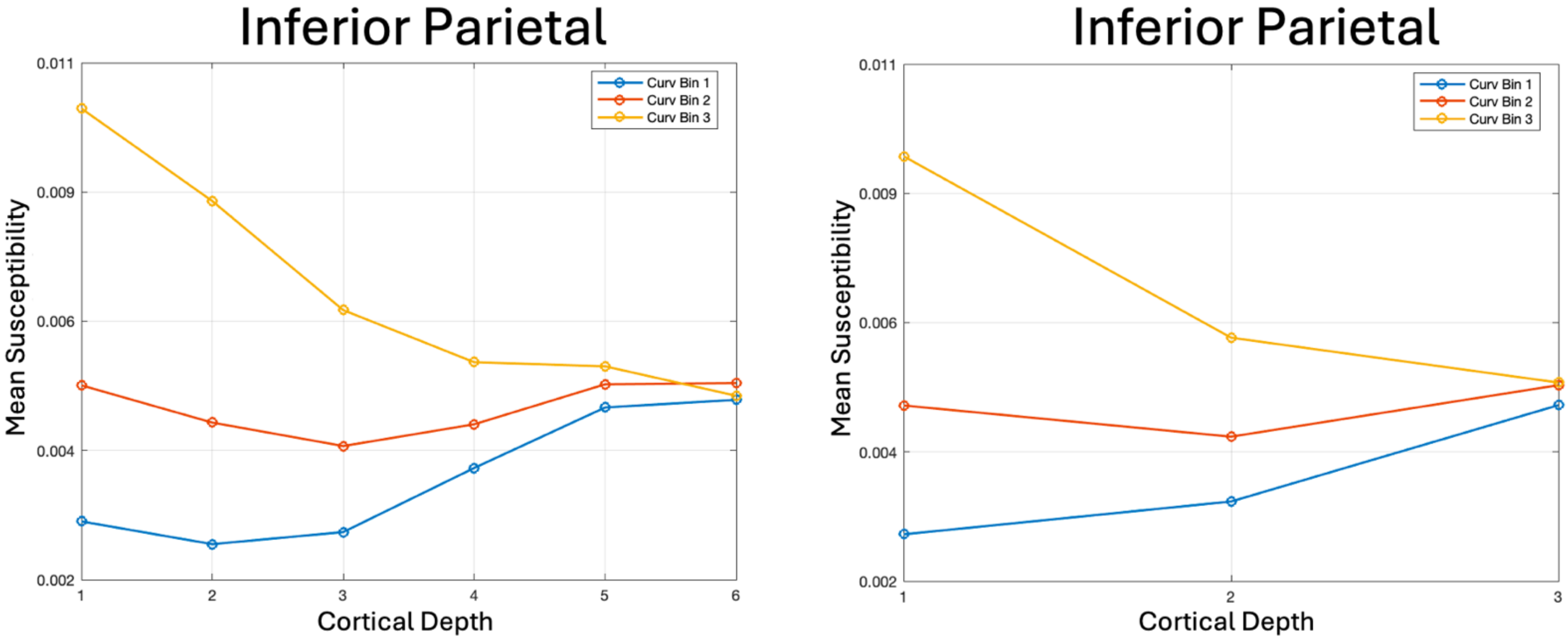

Supplementary Figure 13

Comparative graphs illustrate mean susceptibility values averaged across all participants ($N = 60$) when sampling at six (left) versus three (right) cortical depths for each curvature bin (crown = blue; bank = orange; fundus = yellow) for the postcentral gyrus. While sampling at six depths may introduce some redundancy into the model, the overall susceptibility patterns remain similar. Restricting the analysis to three depths appears to risk overlooking subtle yet informative differences that only emerge at finer depth granularity. Susceptibility is measured in parts per million (ppm).

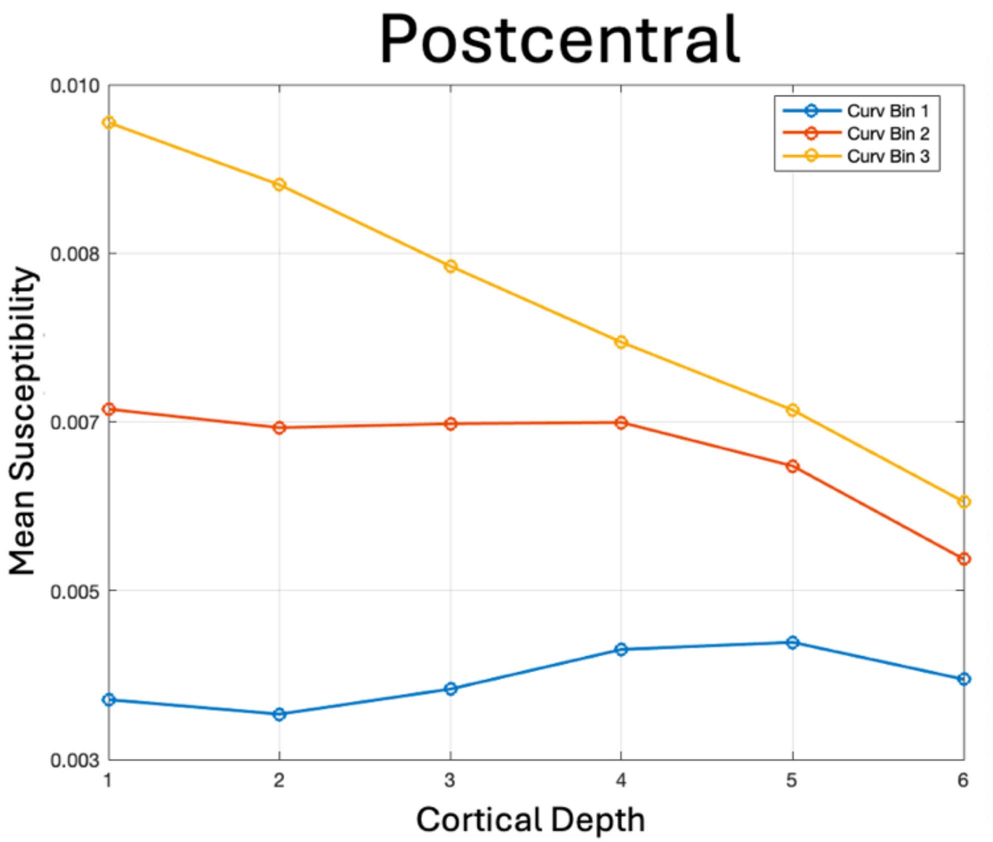

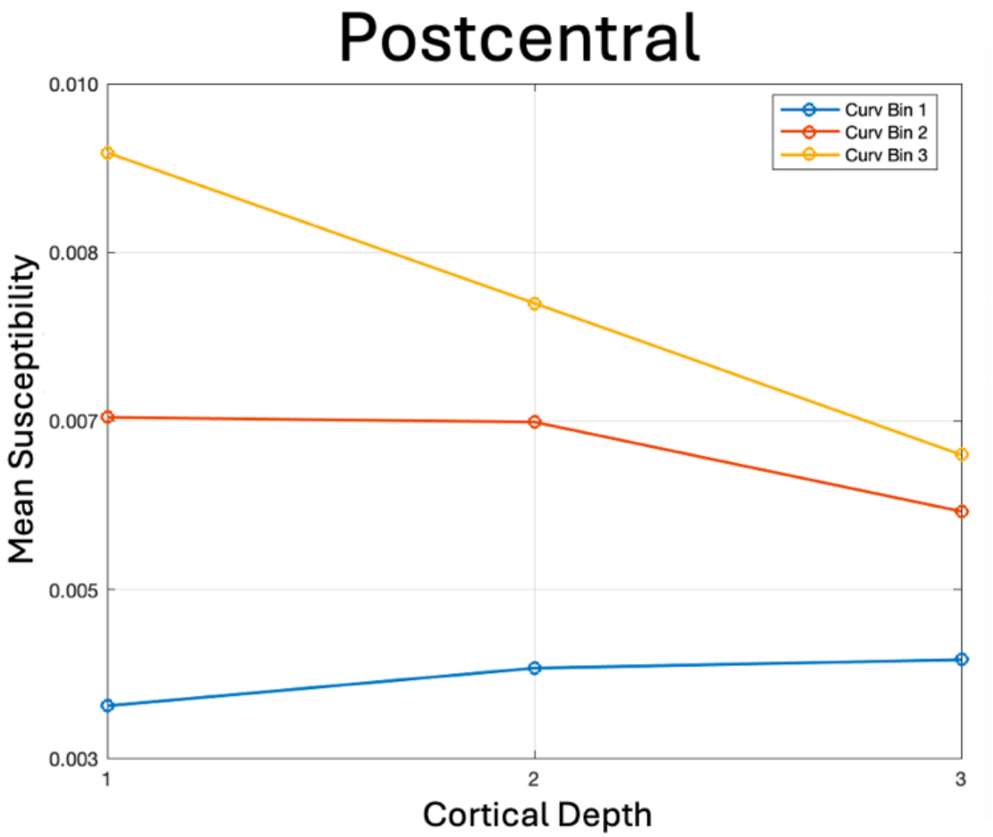

Supplementary Figure 14

Comparative graphs illustrate mean susceptibility values averaged across all participants ($N = 60$) when sampling at six (left) versus three (right) cortical depths for each curvature bin (crown = blue; bank = orange; fundus = yellow) for the supramarginal gyrus. While sampling at six depths may introduce some redundancy into the model, the overall susceptibility patterns remain similar. Restricting the analysis to three depths appears to risk overlooking subtle yet informative differences that only emerge at finer depth granularity. Susceptibility is measured in parts per million (ppm).

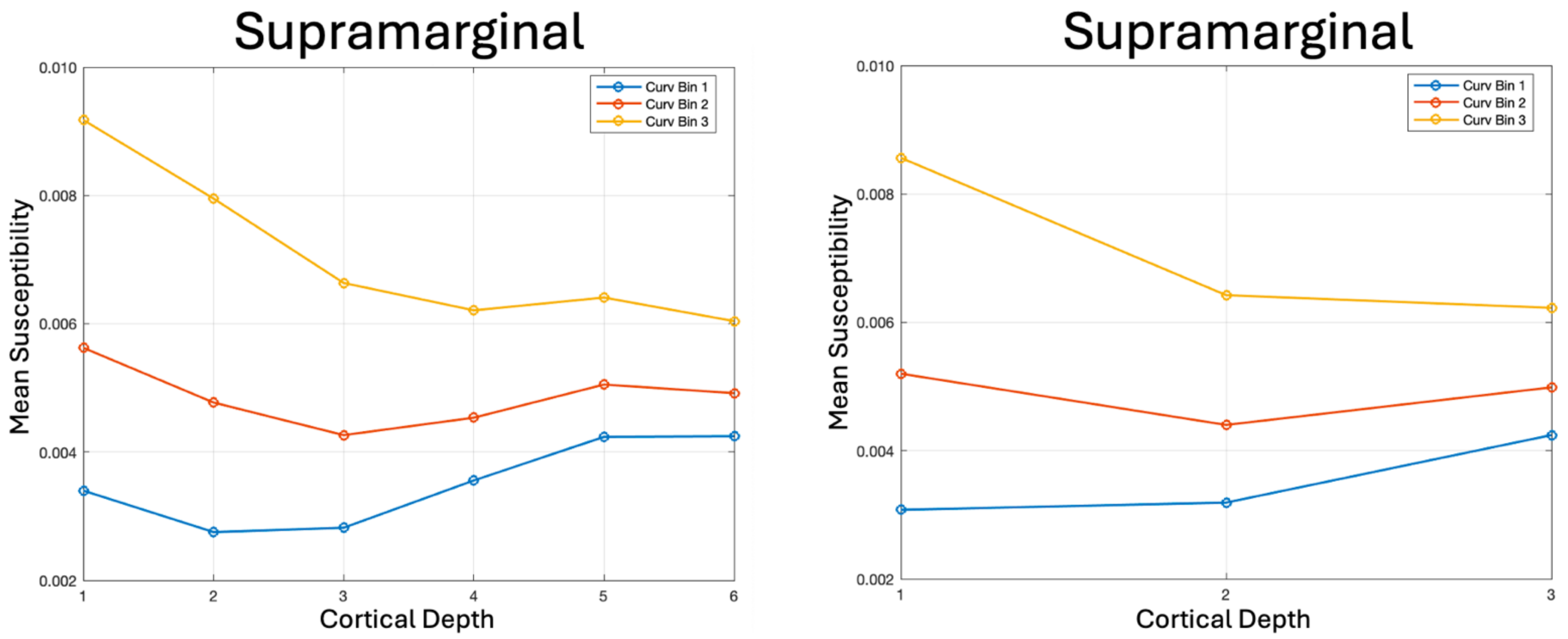

Supplementary Figure 15

Comparative graphs illustrate mean susceptibility values averaged across all participants ($N = 60$) when sampling at six (left) versus three (right) cortical depths for each curvature bin (crown = blue; bank = orange; fundus = yellow) for the precuneus. While sampling at six depths may introduce some redundancy into the model, the overall susceptibility patterns remain similar. Restricting the analysis to three depths appears to risk overlooking subtle yet informative differences that only emerge at finer depth granularity. Susceptibility is measured in parts per million (ppm).

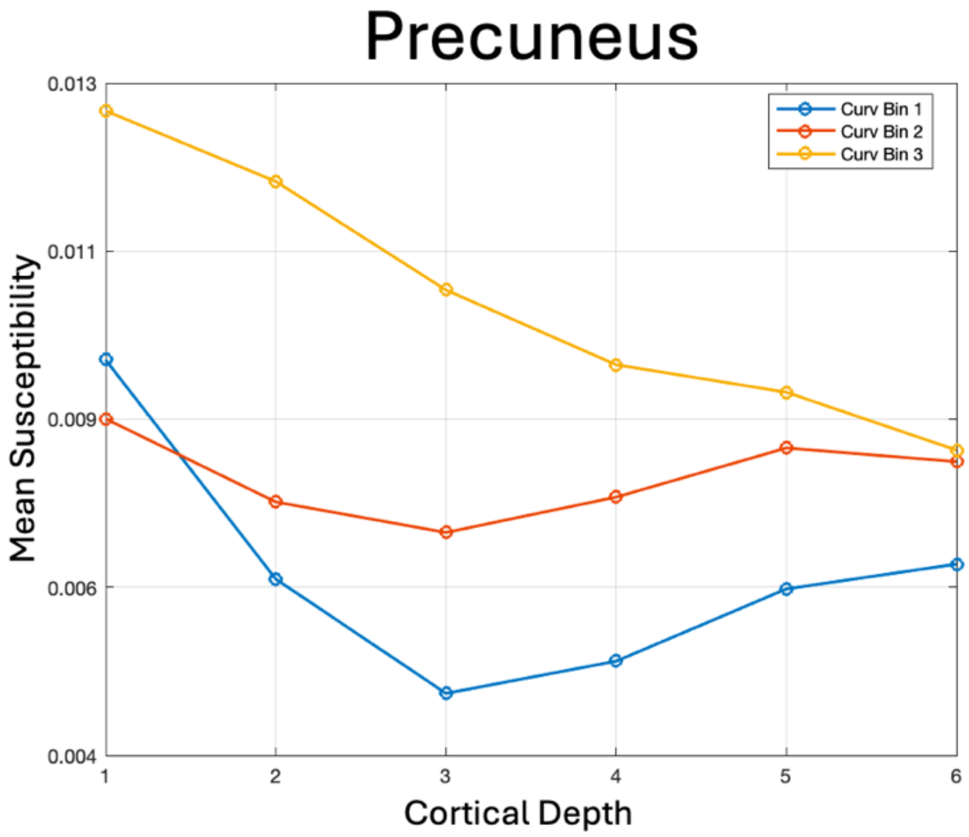

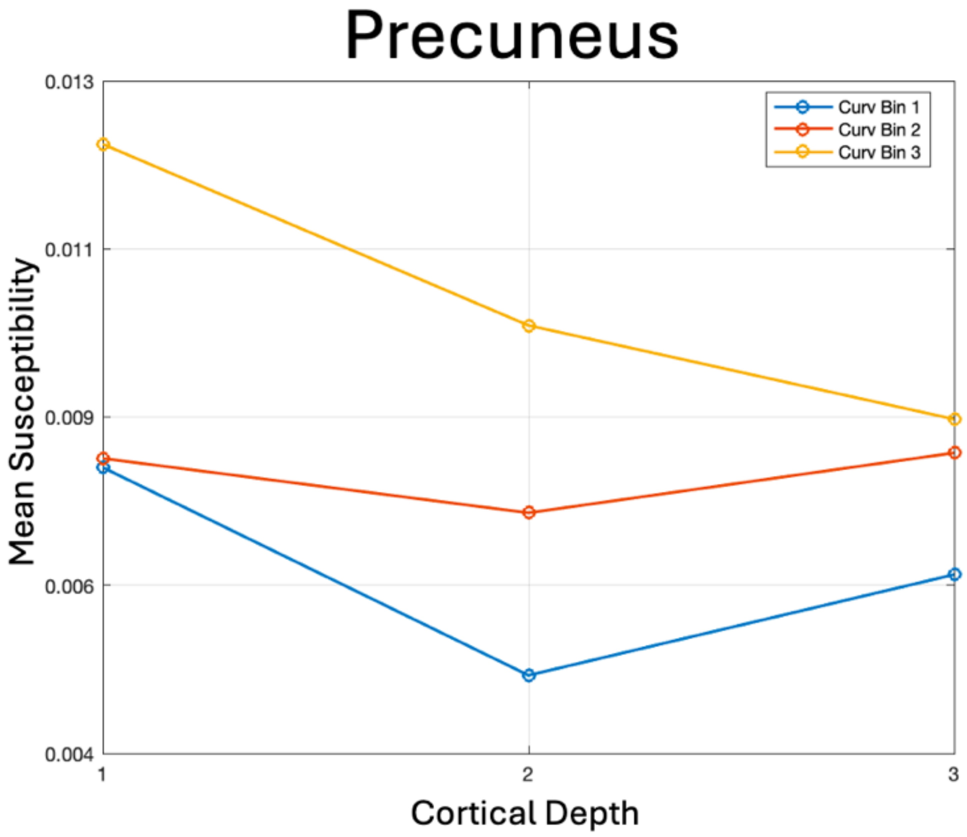

Supplementary Figure 16

Comparative graphs illustrate mean susceptibility values averaged across all participants ($N = 60$) when sampling at six (left) versus three (right) cortical depths for each curvature bin (crown = blue; bank = orange; fundus = yellow) for the paracentral lobule. While sampling at six depths may introduce some redundancy into the model, the overall susceptibility patterns remain similar. Restricting the analysis to three depths appears to risk overlooking subtle yet informative differences that only emerge at finer depth granularity. Susceptibility is measured in parts per million (ppm).

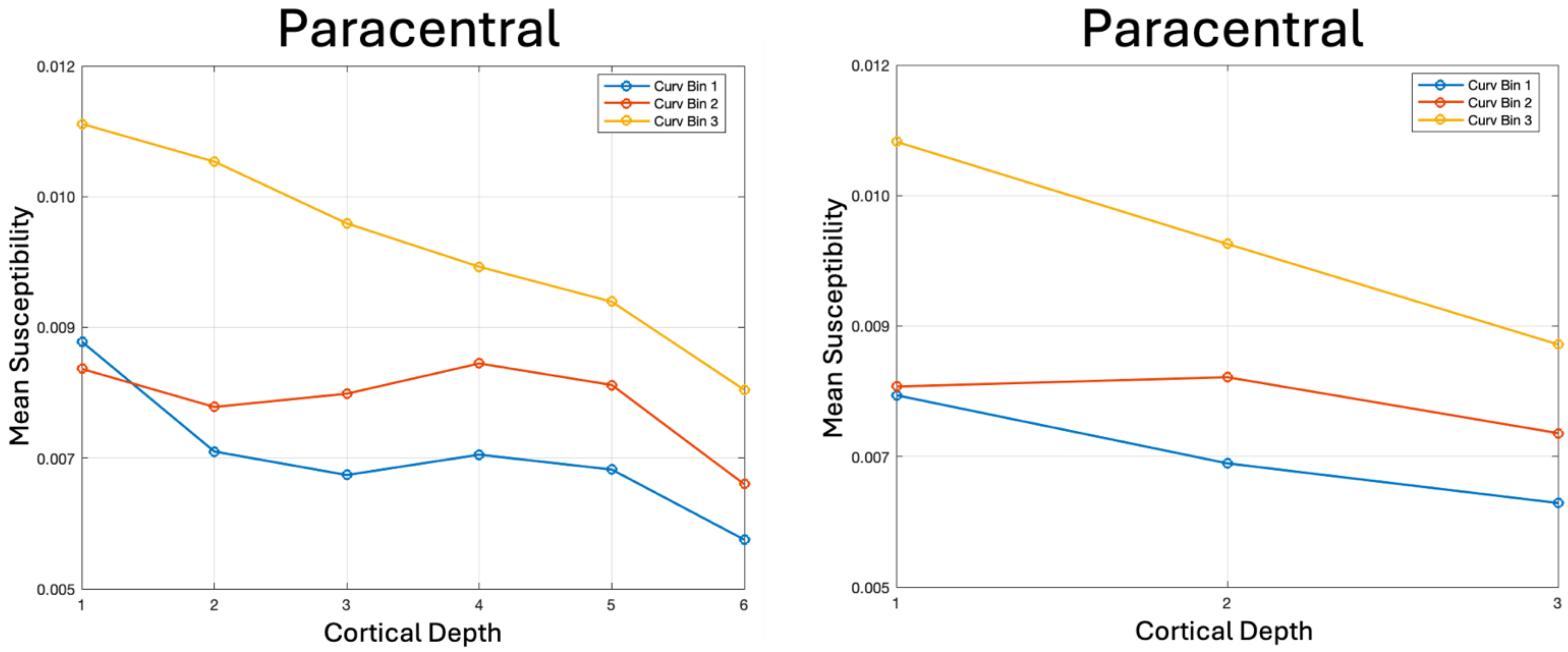

Supplementary Figure 17

Comparative graphs illustrate mean susceptibility values averaged across all participants ($N = 60$) when sampling at six (left) versus three (right) cortical depths for each curvature bin (crown = blue; bank = orange; fundus = yellow) for the bank of the superior temporal sulcus. While sampling at six depths may introduce some redundancy into the model, the overall susceptibility patterns remain similar. Restricting the analysis to three depths appears to risk overlooking subtle yet informative differences that only emerge at finer depth granularity. Susceptibility is measured in parts per million (ppm).

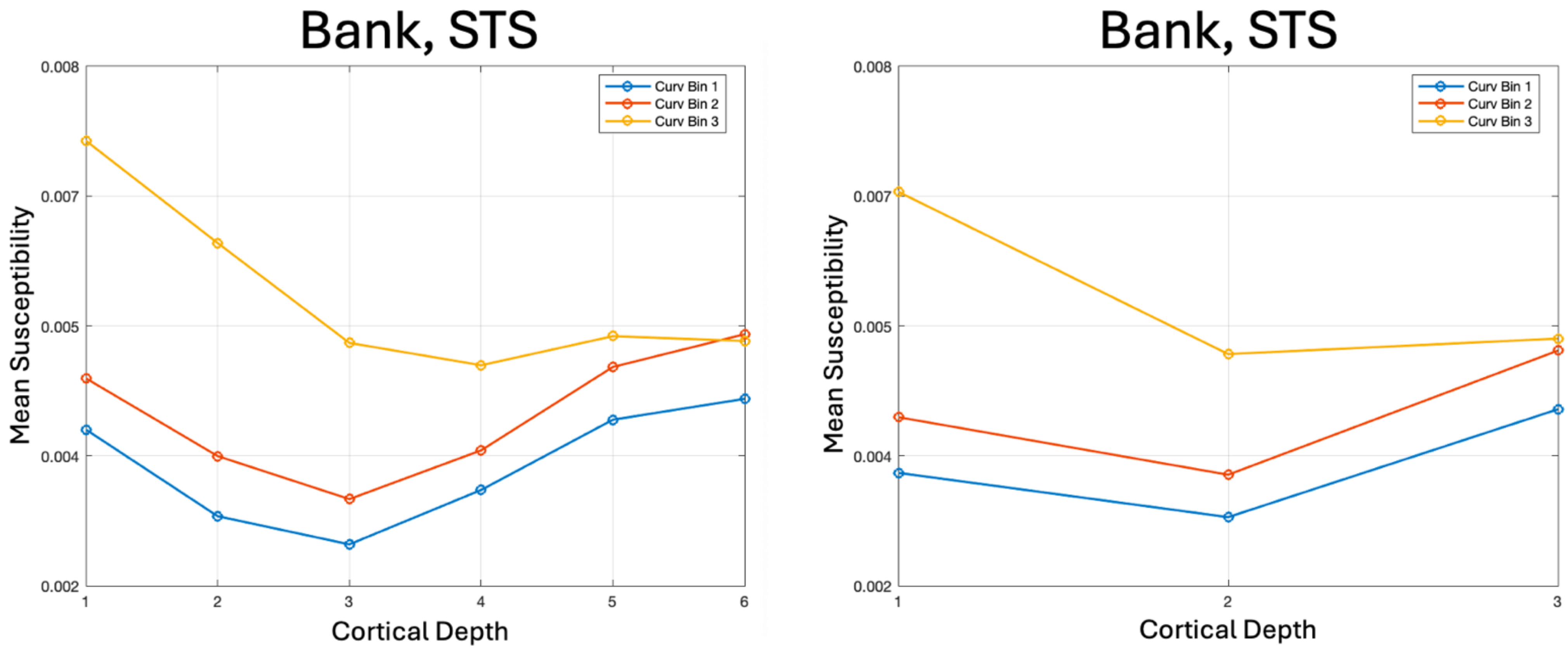

Supplementary Figure 18

Comparative graphs illustrate mean susceptibility values averaged across all participants (*N* = 60) when sampling at six (left) versus three (right) cortical depths for each curvature bin (crown = blue; bank = orange; fundus = yellow) for the superior temporal sulcus. While sampling at six depths may introduce some redundancy into the model, the overall susceptibility patterns remain similar. Restricting the analysis to three depths appears to risk overlooking subtle yet informative differences that only emerge at finer depth granularity. Susceptibility is measured in parts per million (ppm).

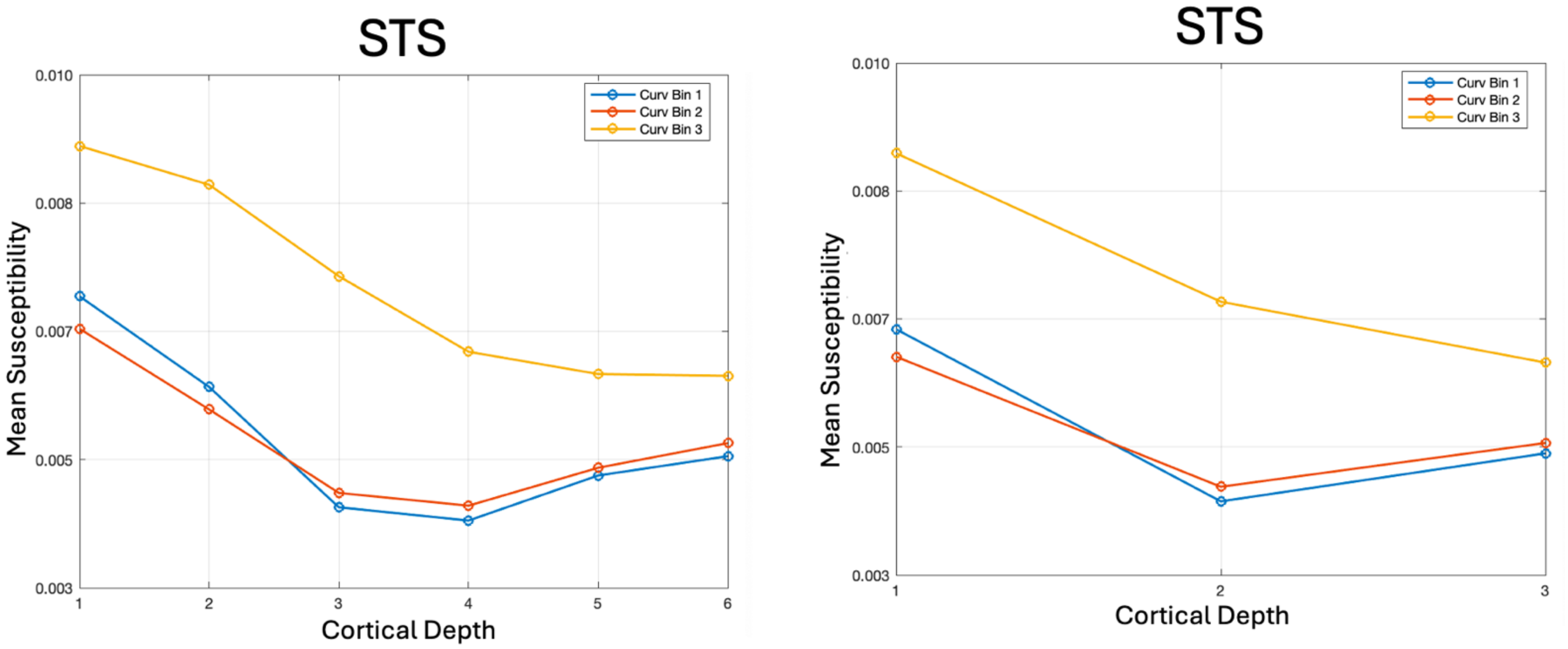

Supplementary Figure 19

Comparative graphs illustrate mean susceptibility values averaged across all participants (*N* = 60) when sampling at six (left) versus three (right) cortical depths for each curvature bin (crown = blue; bank = orange; fundus = yellow) for the middle temporal gyrus. While sampling at six depths may introduce some redundancy into the model, the overall susceptibility patterns remain similar. Restricting the analysis to three depths appears to risk overlooking subtle yet informative differences that only emerge at finer depth granularity. Susceptibility is measured in parts per million (ppm).

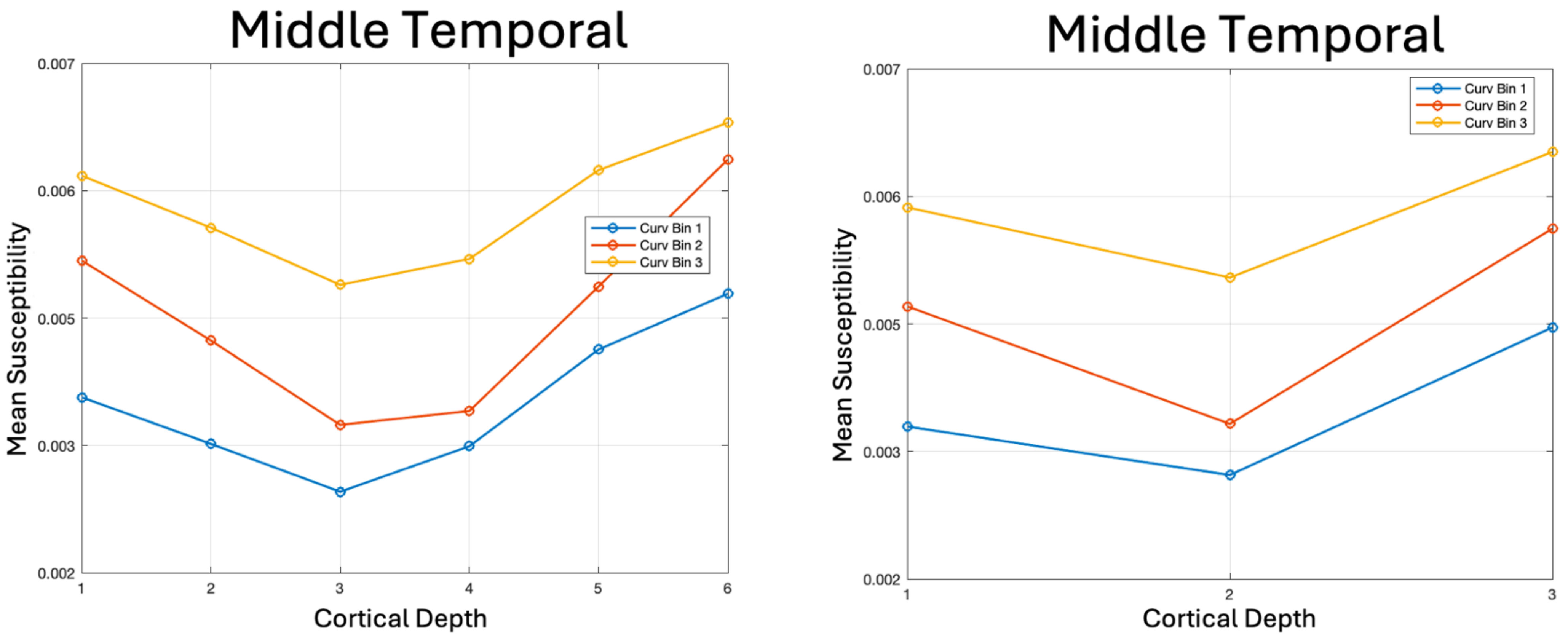

Supplementary Figure 20

Comparative graphs illustrate mean susceptibility values averaged across all participants (*N* = 60) when sampling at six (left) versus three (right) cortical depths for each curvature bin (crown = blue; bank = orange; fundus = yellow) for the temporal pole. While sampling at six depths may introduce some redundancy into the model, the overall susceptibility patterns remain similar. Restricting the analysis to three depths appears to risk overlooking subtle yet informative differences that only emerge at finer depth granularity. Susceptibility is measured in parts per million (ppm).

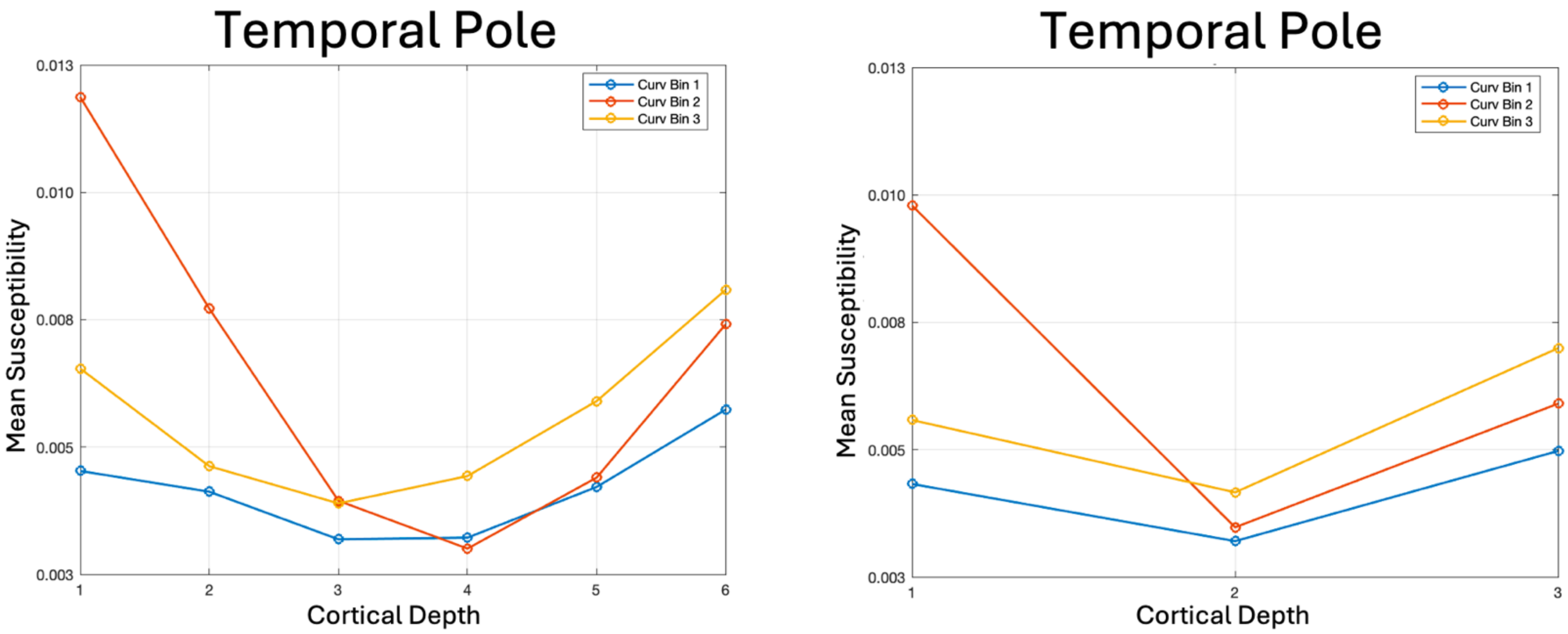

Supplementary Figure 21

Comparative graphs illustrate mean susceptibility values averaged across all participants (*N* = 60) when sampling at six (left) versus three (right) cortical depths for each curvature bin (crown = blue; bank = orange; fundus = yellow) for the transverse temporal gyrus. While sampling at six depths may introduce some redundancy into the model, the overall susceptibility patterns remain similar. Restricting the analysis to three depths appears to risk overlooking subtle yet informative differences that only emerge at finer depth granularity. Susceptibility is measured in parts per million (ppm).

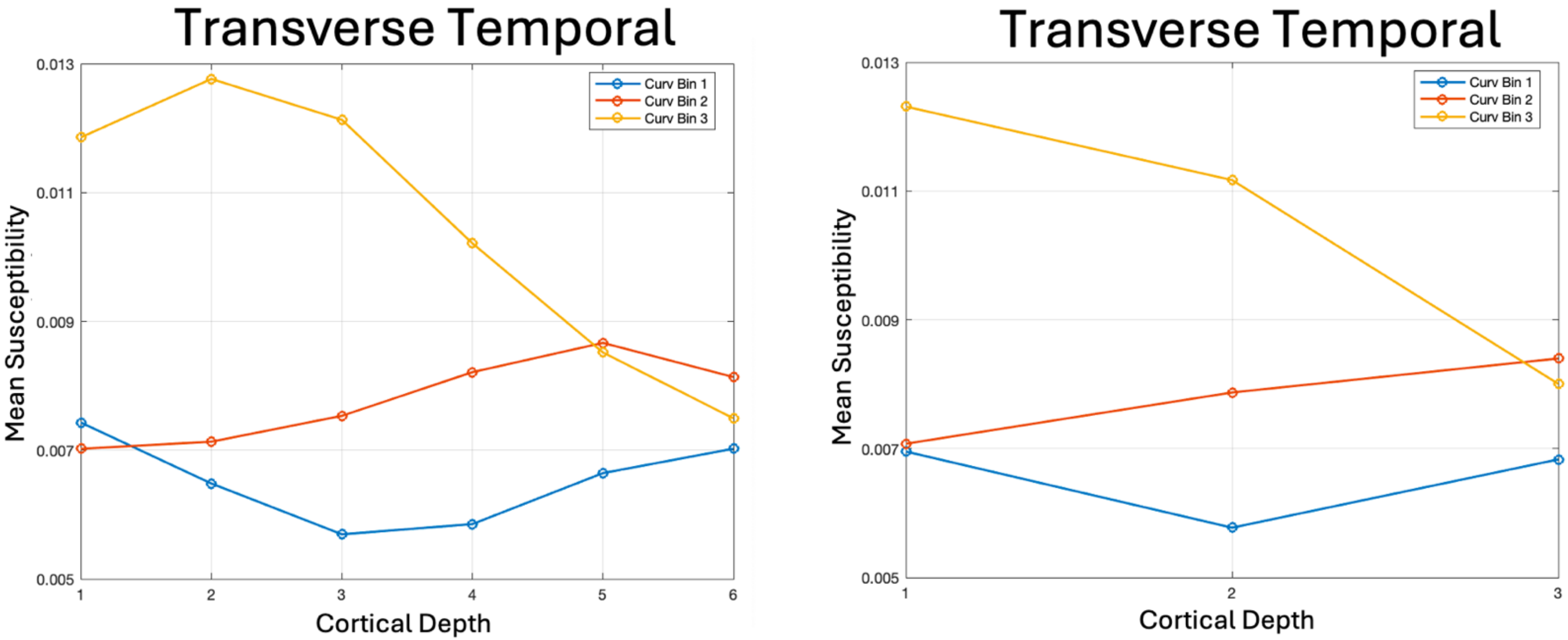

Supplementary Figure 22

Comparative graphs illustrate mean susceptibility values averaged across all participants ($N = 60$) when sampling at six (left) versus three (right) cortical depths for each curvature bin (crown = blue; bank = orange; fundus = yellow) for the parahippocampal gyrus. While sampling at six depths may introduce some redundancy into the model, the overall susceptibility patterns remain similar. Restricting the analysis to three depths appears to risk overlooking subtle yet informative differences that only emerge at finer depth granularity. Susceptibility is measured in parts per million (ppm).

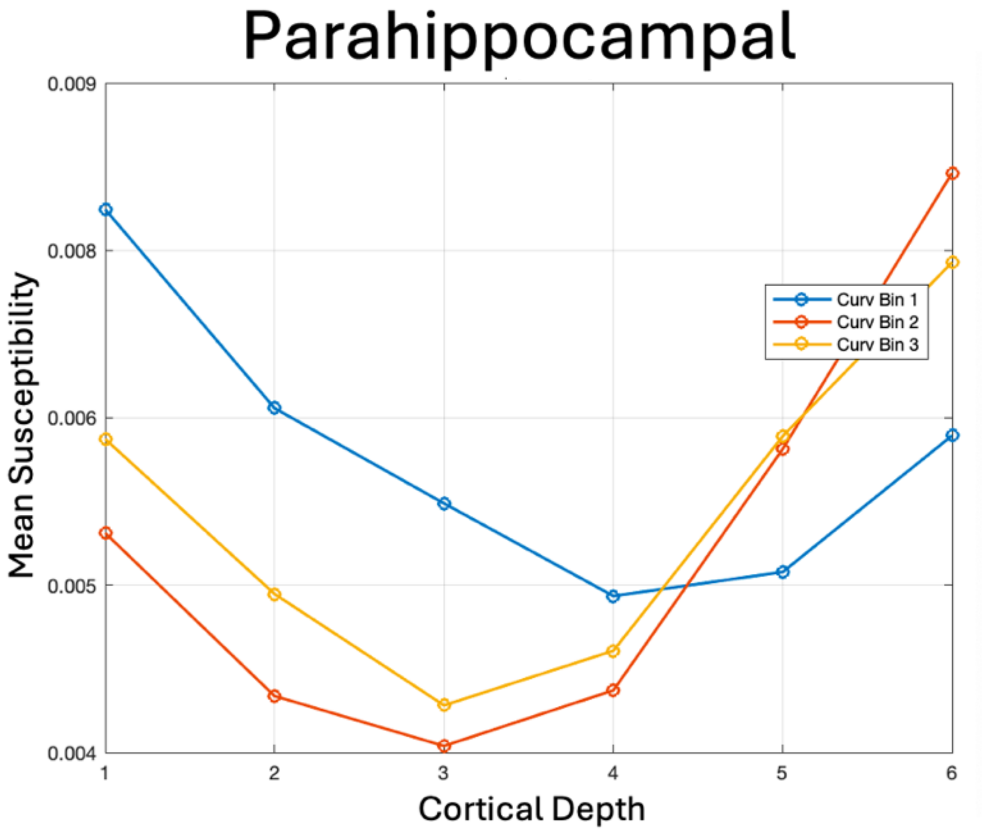

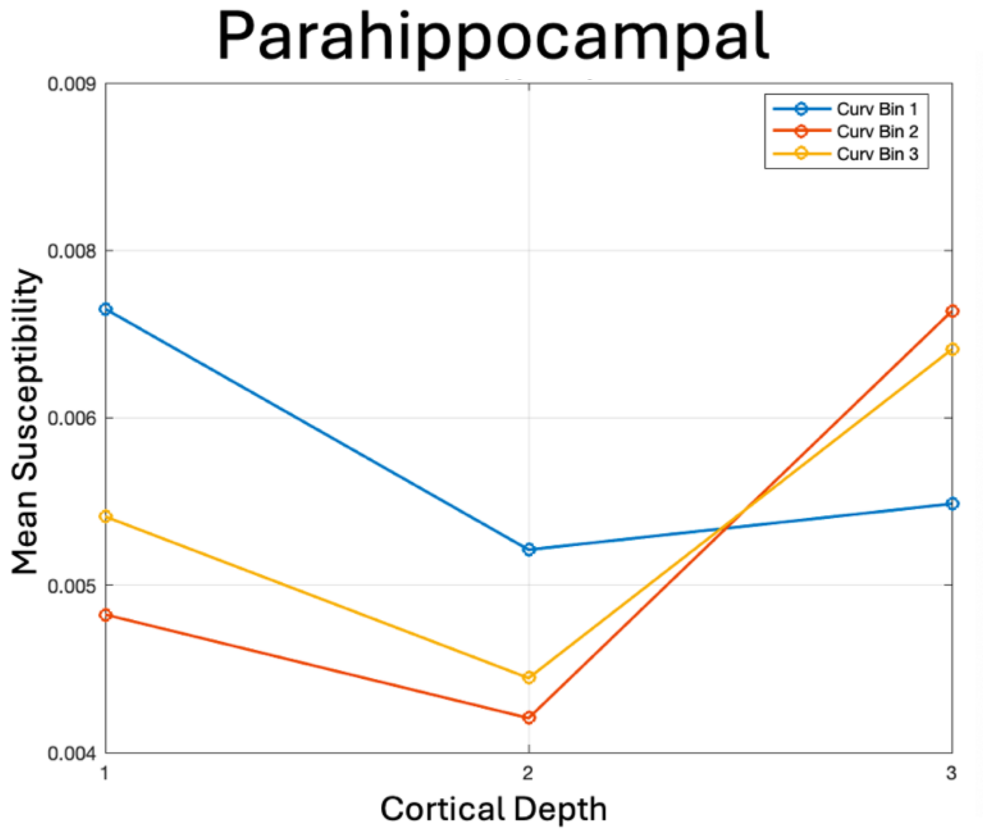

Supplementary Figure 23

Comparative graphs illustrate mean susceptibility values averaged across all participants ($N = 60$) when sampling at six (left) versus three (right) cortical depths for each curvature bin (crown = blue; bank = orange; fundus = yellow) for the entorhinal cortex. While sampling at six depths may introduce some redundancy into the model, the overall susceptibility patterns remain similar. Restricting the analysis to three depths appears to risk overlooking subtle yet informative differences that only emerge at finer depth granularity. Susceptibility is measured in parts per million (ppm).

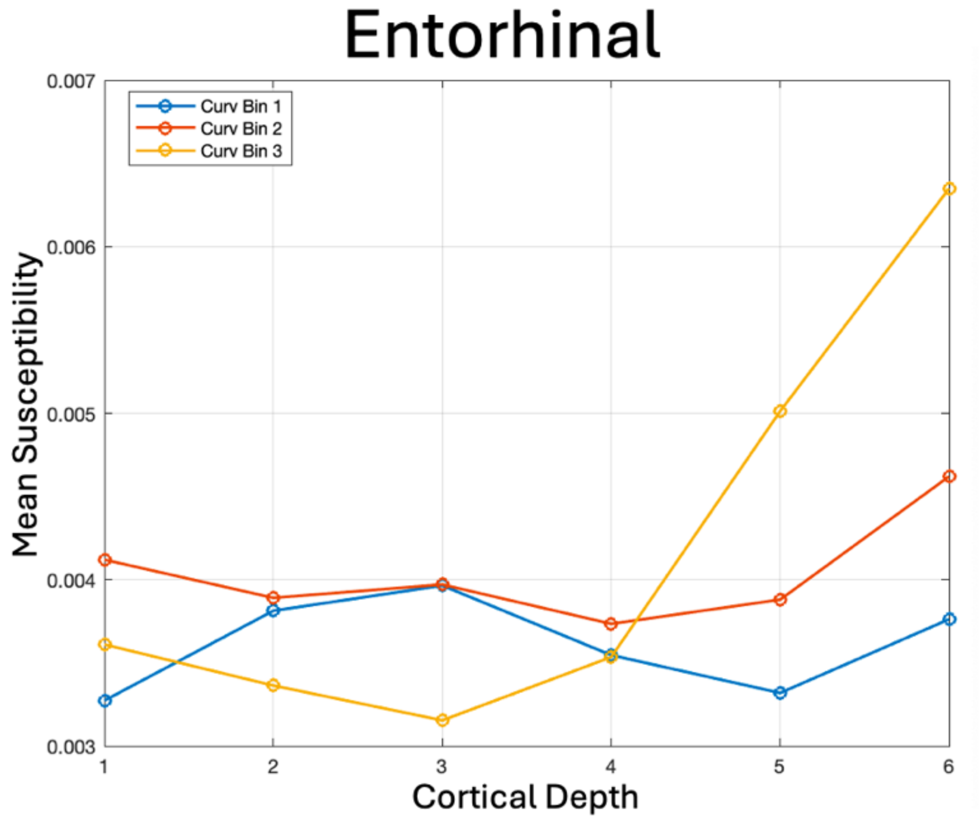

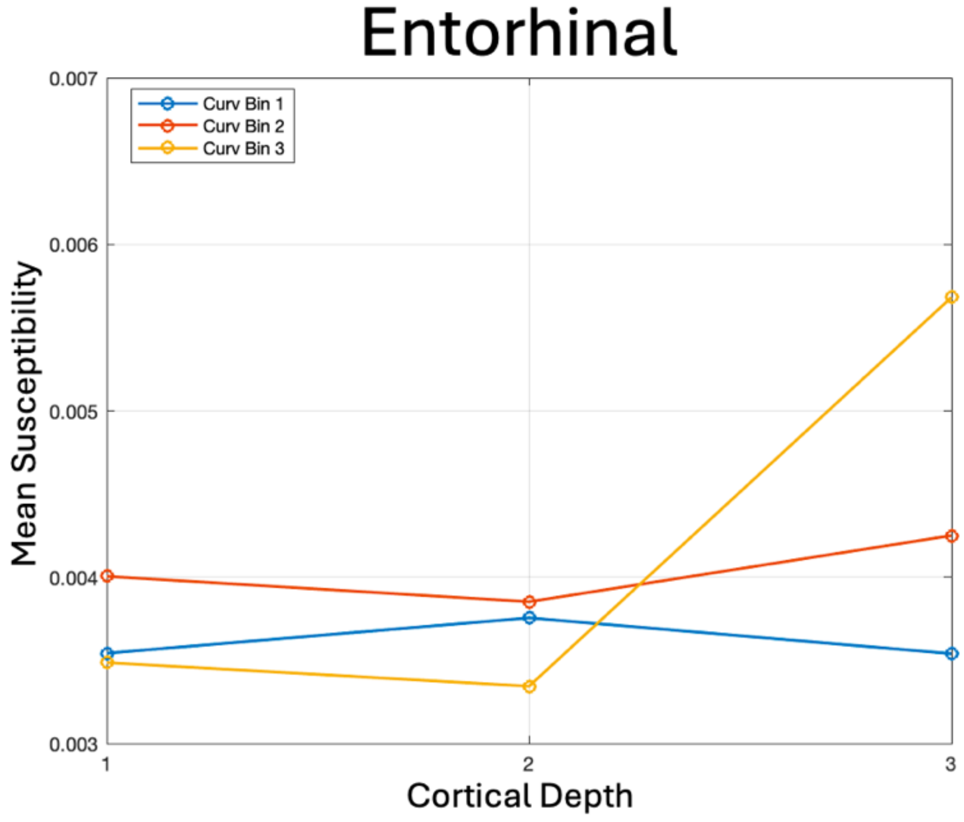

Supplementary Figure 24

Comparative graphs illustrate mean susceptibility values averaged across all participants ($N = 60$) when sampling at six (left) versus three (right) cortical depths for each curvature bin (crown = blue; bank = orange; fundus = yellow) for the fusiform gyrus. While sampling at six depths may introduce some redundancy into the model, the overall susceptibility patterns remain similar. Restricting the analysis to three depths appears to risk overlooking subtle yet informative differences that only emerge at finer depth granularity. Susceptibility is measured in parts per million (ppm).

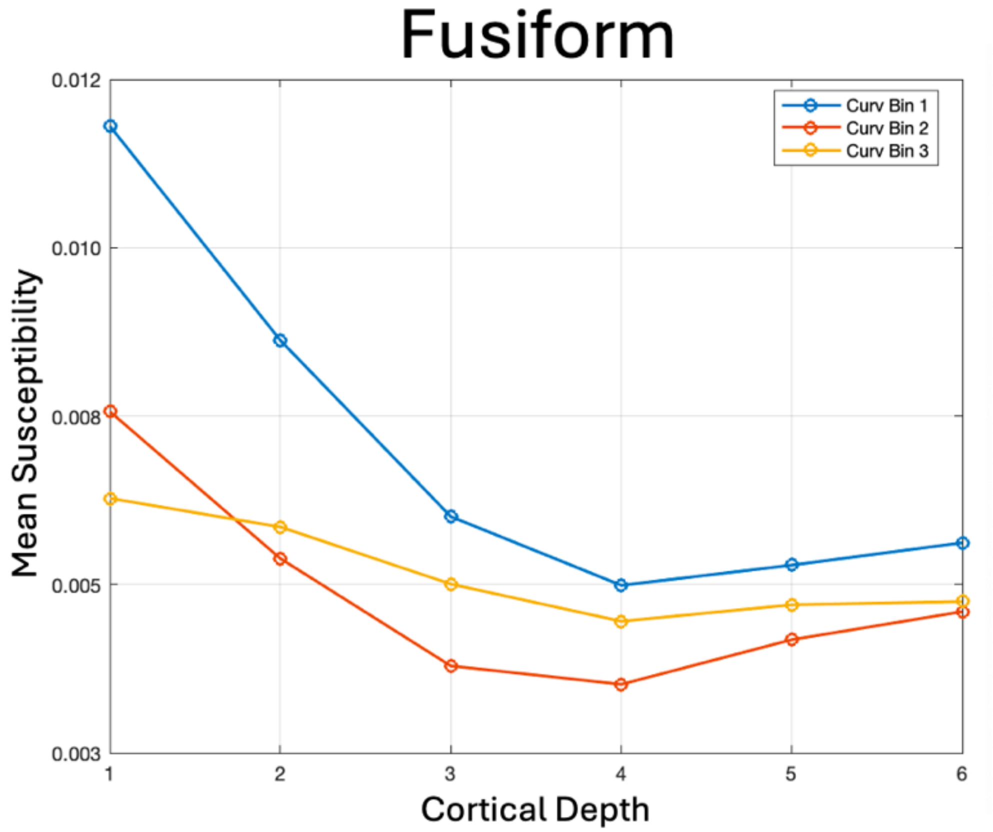

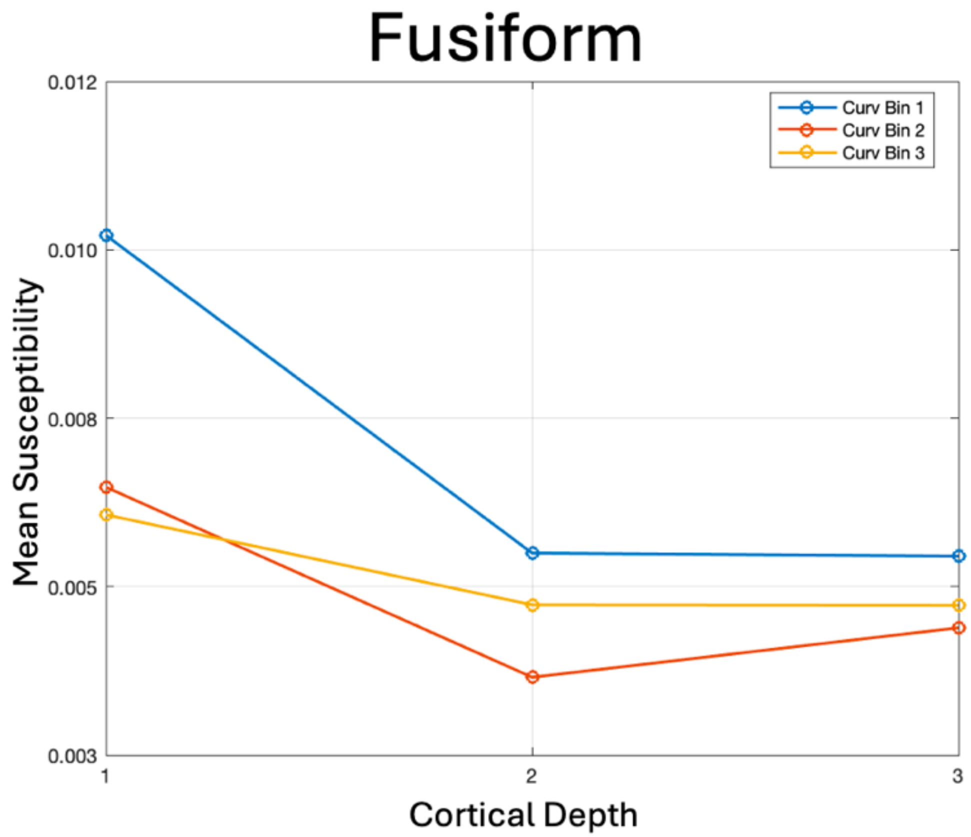

Supplementary Figure 25

Comparative graphs illustrate mean susceptibility values averaged across all participants ($N = 60$) when sampling at six (left) versus three (right) cortical depths for each curvature bin (crown = blue; bank = orange; fundus = yellow) for the inferior temporal gyrus. While sampling at six depths may introduce some redundancy into the model, the overall susceptibility patterns remain similar. Restricting the analysis to three depths appears to risk overlooking subtle yet informative differences that only emerge at finer depth granularity. Susceptibility is measured in parts per million (ppm).

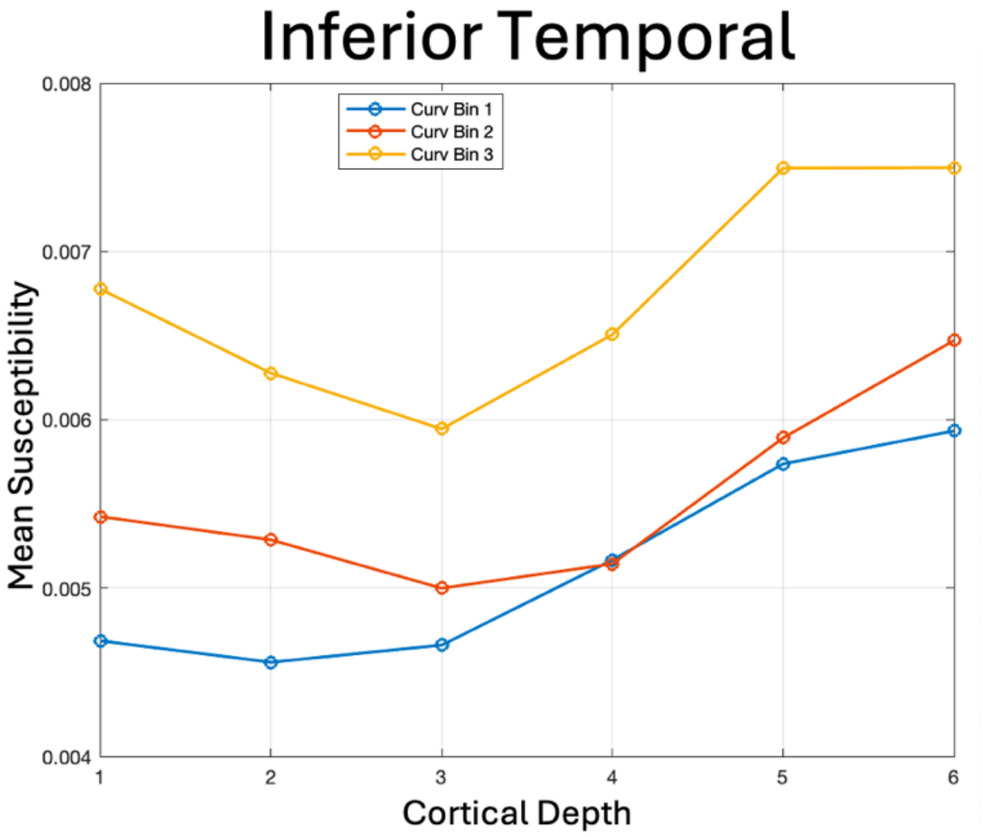

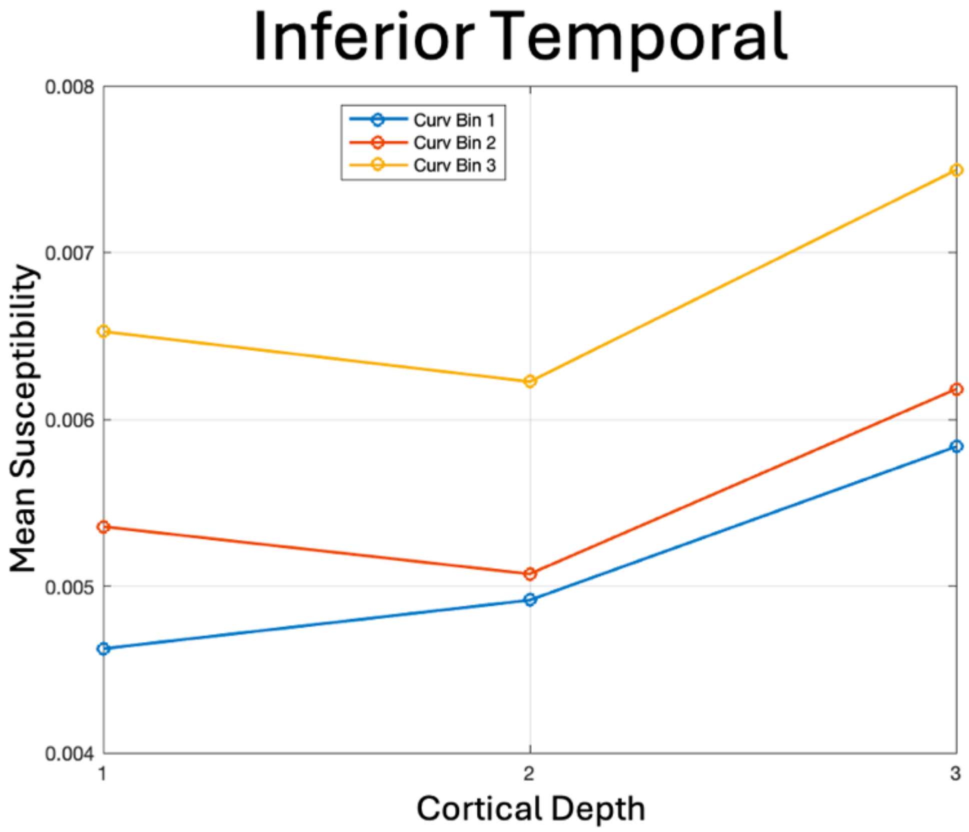

Supplementary Figure 26

Comparative graphs illustrate mean susceptibility values averaged across all participants (*N* = 60) when sampling at six (left) versus three (right) cortical depths for each curvature bin (crown = blue; bank = orange; fundus = yellow) for the lateral occipital cortex. While sampling at six depths may introduce some redundancy into the model, the overall susceptibility patterns remain similar. Restricting the analysis to three depths appears to risk overlooking subtle yet informative differences that only emerge at finer depth granularity. Susceptibility is measured in parts per million (ppm).

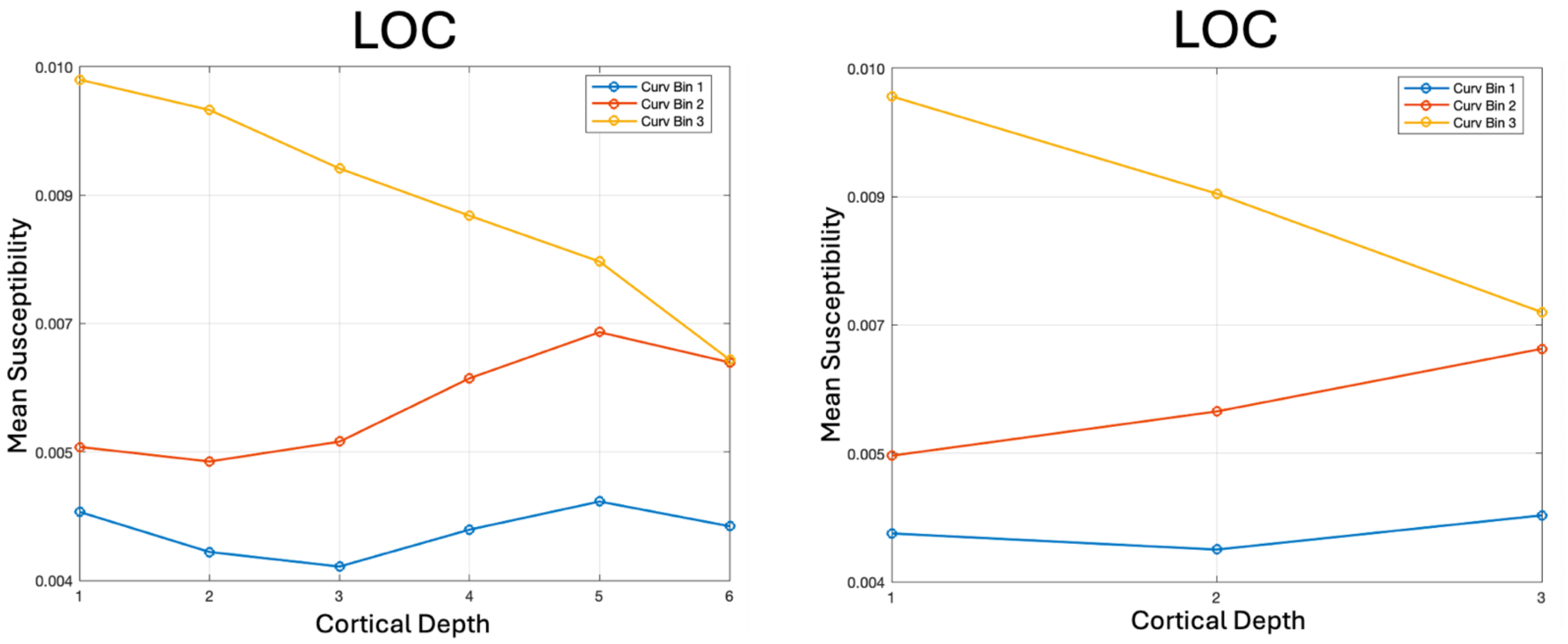

Supplementary Figure 27

Comparative graphs illustrate mean susceptibility values averaged across all participants (*N* = 60) when sampling at six (left) versus three (right) cortical depths for each curvature bin (crown = blue; bank = orange; fundus = yellow) for the lingual gyrus. While sampling at six depths may introduce some redundancy into the model, the overall susceptibility patterns remain similar. Restricting the analysis to three depths appears to risk overlooking subtle yet informative differences that only emerge at finer depth granularity. Susceptibility is measured in parts per million (ppm).

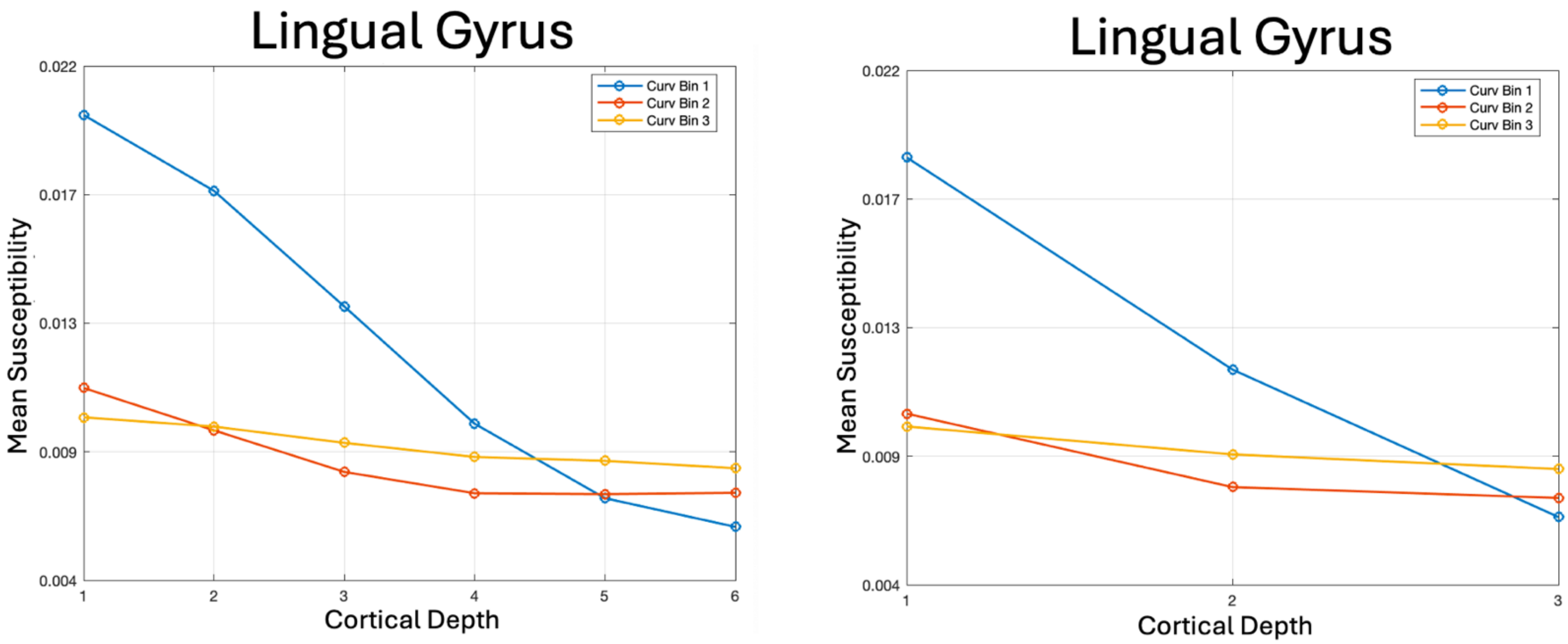

Supplementary Figure 28

Comparative graphs illustrate mean susceptibility values averaged across all participants ($N = 60$) when sampling at six (left) versus three (right) cortical depths for each curvature bin (crown = blue; bank = orange; fundus = yellow) for the pericalcarine cortex. While sampling at six depths may introduce some redundancy into the model, the overall susceptibility patterns remain similar. Restricting the analysis to three depths appears to risk overlooking subtle yet informative differences that only emerge at finer depth granularity. Susceptibility is measured in parts per million (ppm).

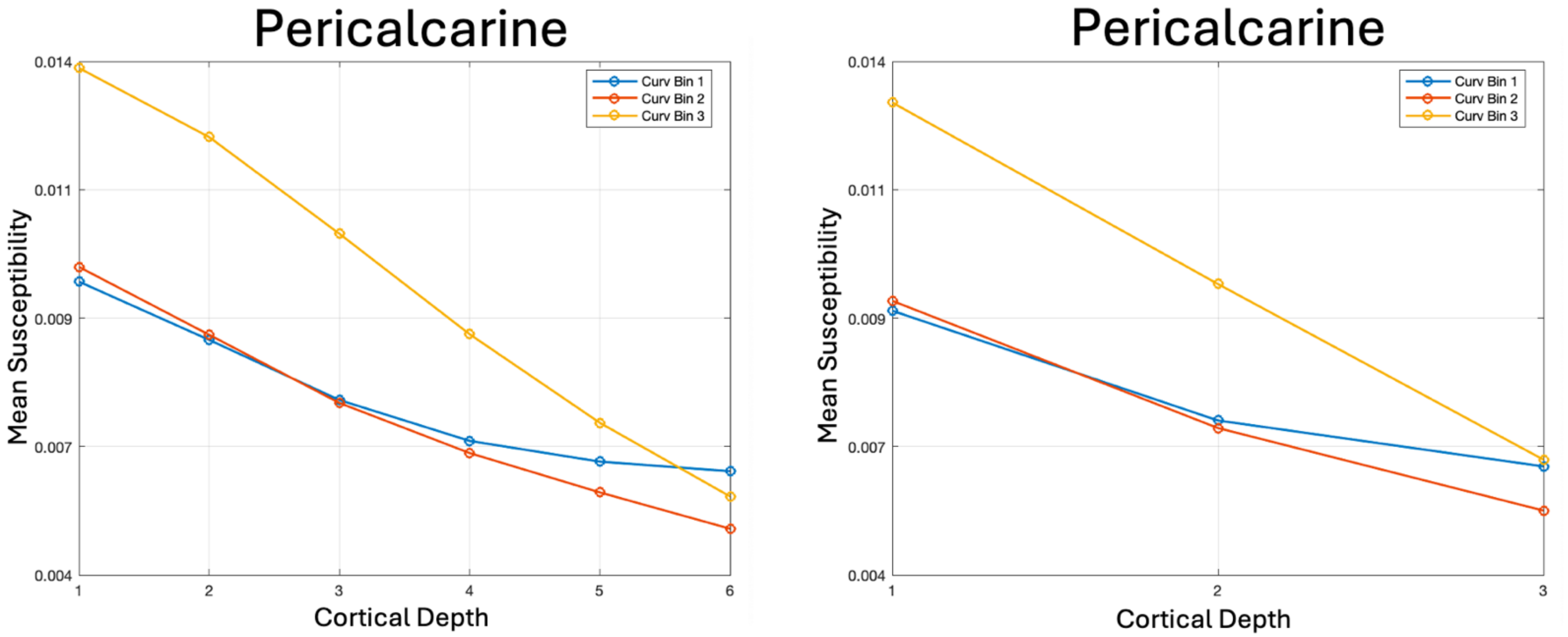

Supplementary Figure 29

Comparative graphs illustrate mean susceptibility values averaged across all participants (*N* = 60) when sampling at six (left) versus three (right) cortical depths for each curvature bin (crown = blue; bank = orange; fundus = yellow) for the cuneus. While sampling at six depths may introduce some redundancy into the model, the overall susceptibility patterns remain similar. Restricting the analysis to three depths appears to risk overlooking subtle yet informative differences that only emerge at finer depth granularity. Susceptibility is measured in parts per million (ppm).

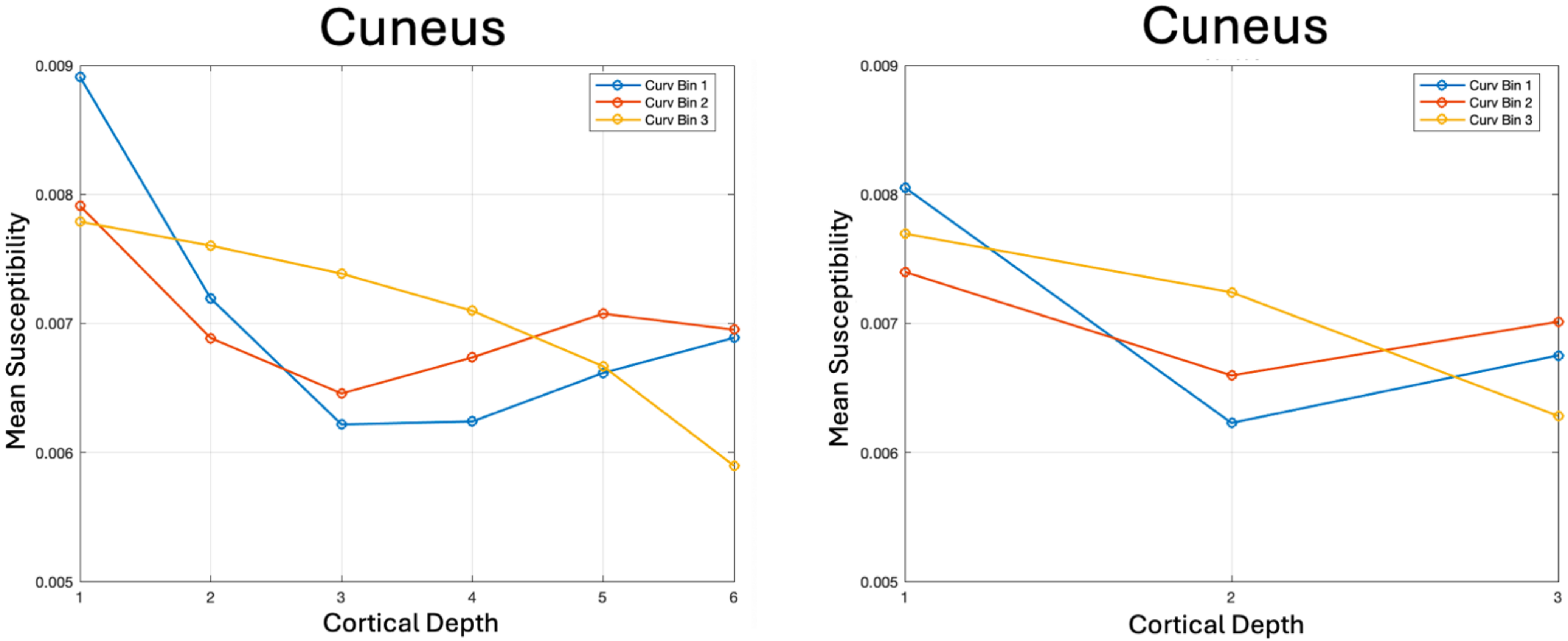

Supplementary Figure 30

Comparative graphs illustrate mean susceptibility values averaged across all participants ($N = 60$) when sampling at six (left) versus three (right) cortical depths for each curvature bin (crown = blue; bank = orange; fundus = yellow) for the insula. While sampling at six depths may introduce some redundancy into the model, the overall susceptibility patterns remain similar. Restricting the analysis to three depths appears to risk overlooking subtle yet informative differences that only emerge at finer depth granularity. Susceptibility is measured in parts per million (ppm).

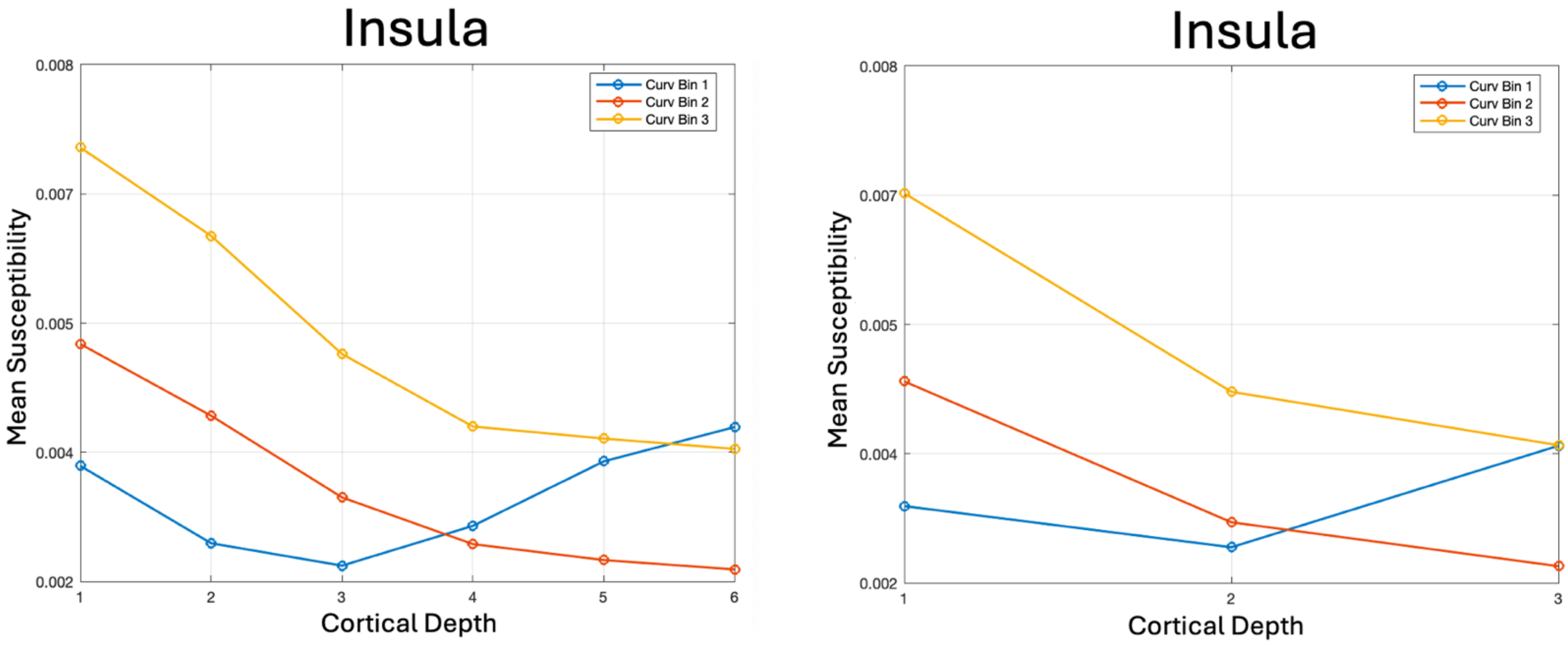

Supplementary Figure 31

Comparative graphs illustrate mean susceptibility values averaged across all participants ($N = 60$) when sampling at six (left) versus three (right) cortical depths for each curvature bin (crown = blue; bank = orange; fundus = yellow) for the rostral anterior cingulate cortex. While sampling at six depths may introduce some redundancy into the model, the overall susceptibility patterns remain similar. Restricting the analysis to three depths appears to risk overlooking subtle yet informative differences that only emerge at finer depth granularity. Susceptibility is measured in parts per million (ppm).

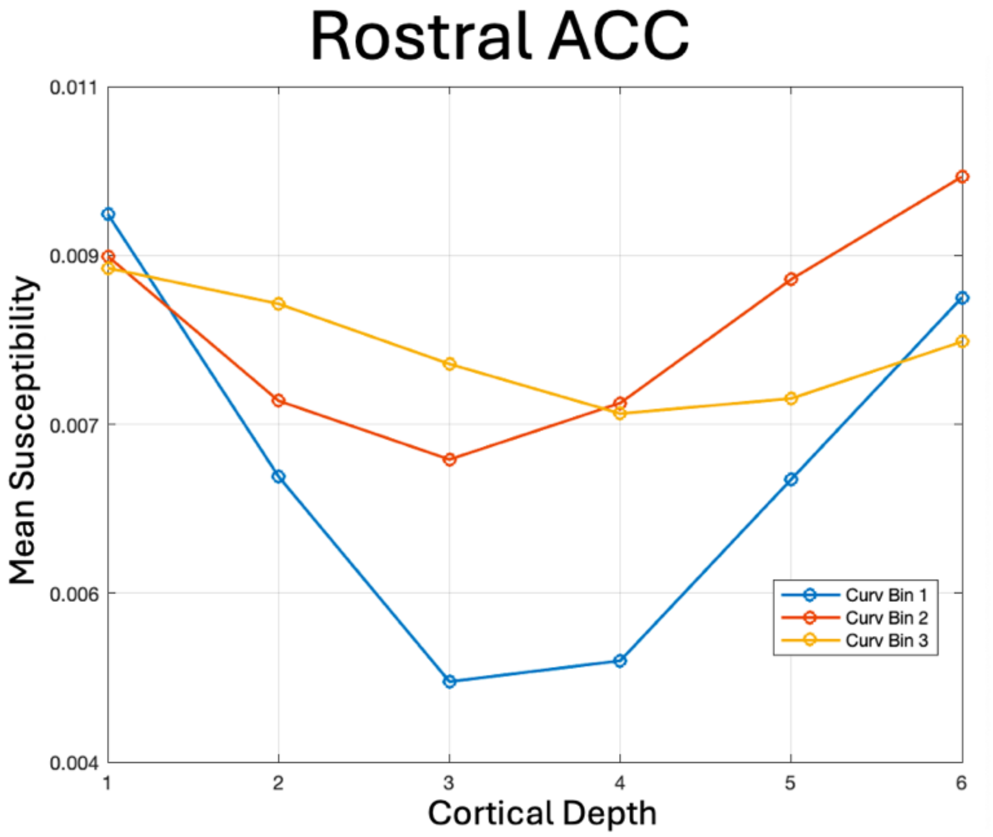

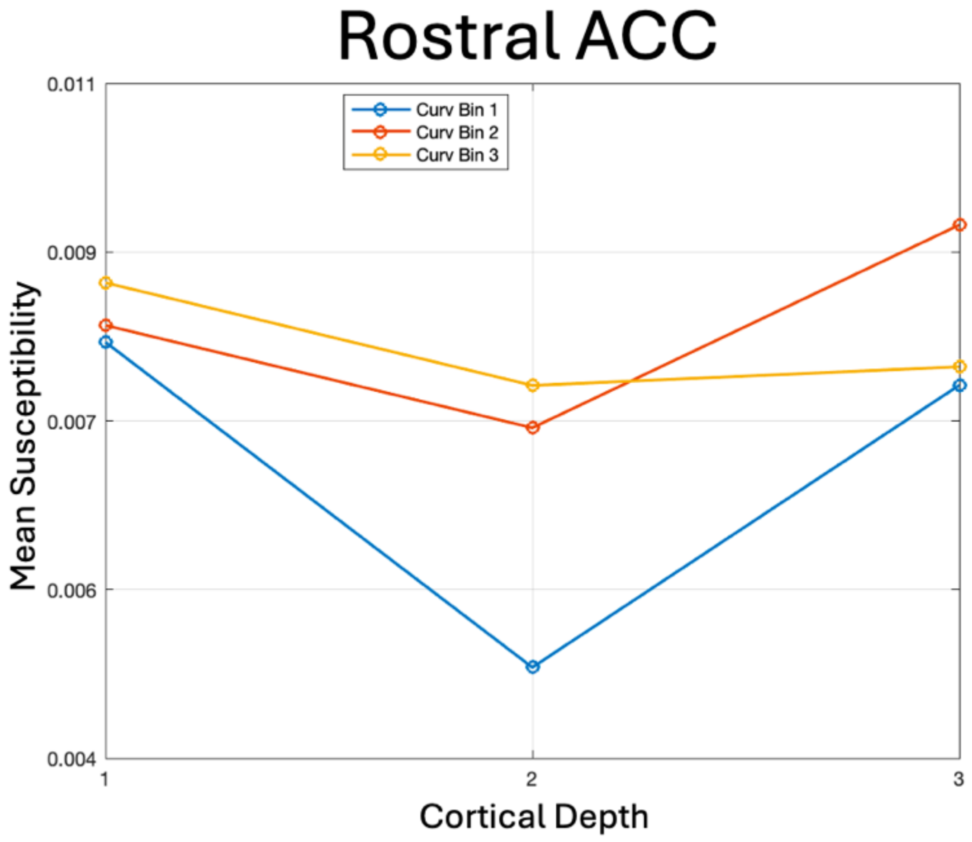

Supplementary Figure 32

Comparative graphs illustrate mean susceptibility values averaged across all participants ($N = 60$) when sampling at six (left) versus three (right) cortical depths for each curvature bin (crown = blue; bank = orange; fundus = yellow) for the caudal anterior cingulate cortex. While sampling at six depths may introduce some redundancy into the model, the overall susceptibility patterns remain similar. Restricting the analysis to three depths appears to risk overlooking subtle yet informative differences that only emerge at finer depth granularity. Susceptibility is measured in parts per million (ppm).

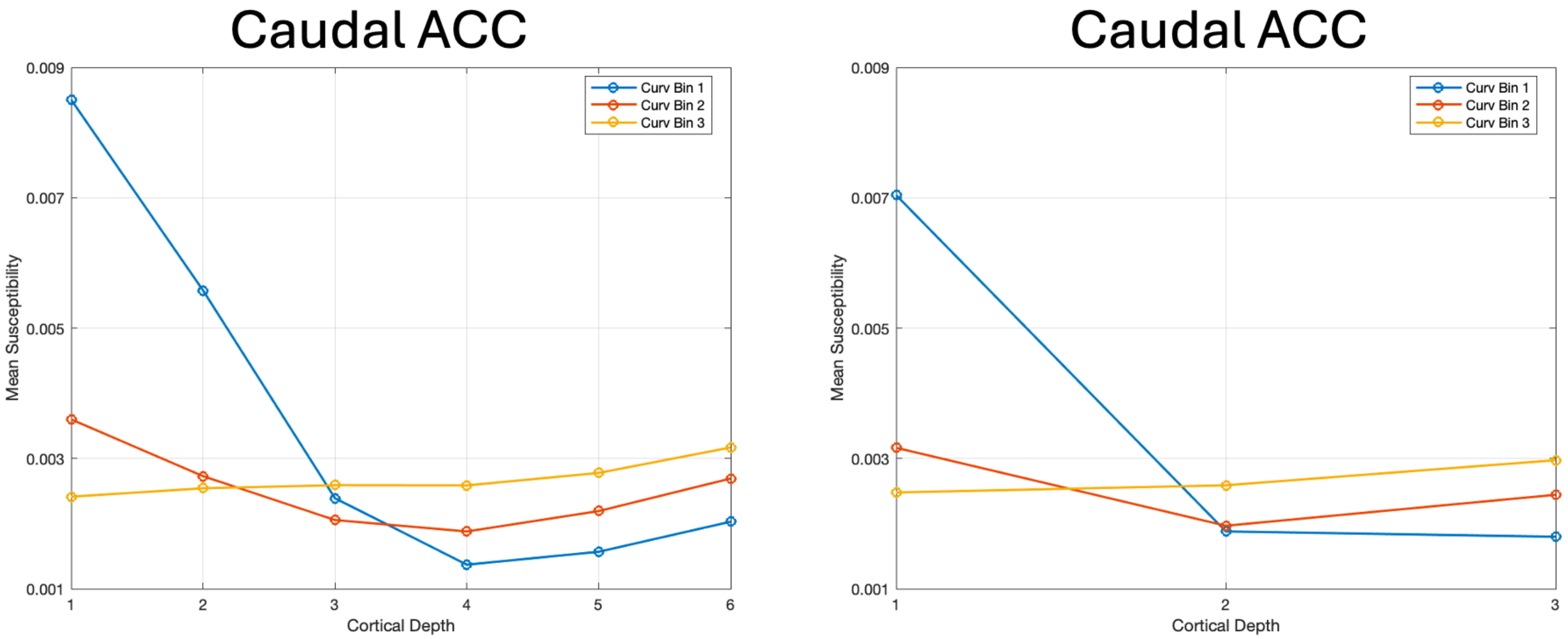

Supplementary Figure 33

Comparative graphs illustrate mean susceptibility values averaged across all participants ($N = 60$) when sampling at six (left) versus three (right) cortical depths for each curvature bin (crown = blue; bank = orange; fundus = yellow) for the posterior cingulate. While sampling at six depths may introduce some redundancy into the model, the overall susceptibility patterns remain similar. Restricting the analysis to three depths appears to risk overlooking subtle yet informative differences that only emerge at finer depth granularity. Susceptibility is measured in parts per million (ppm).

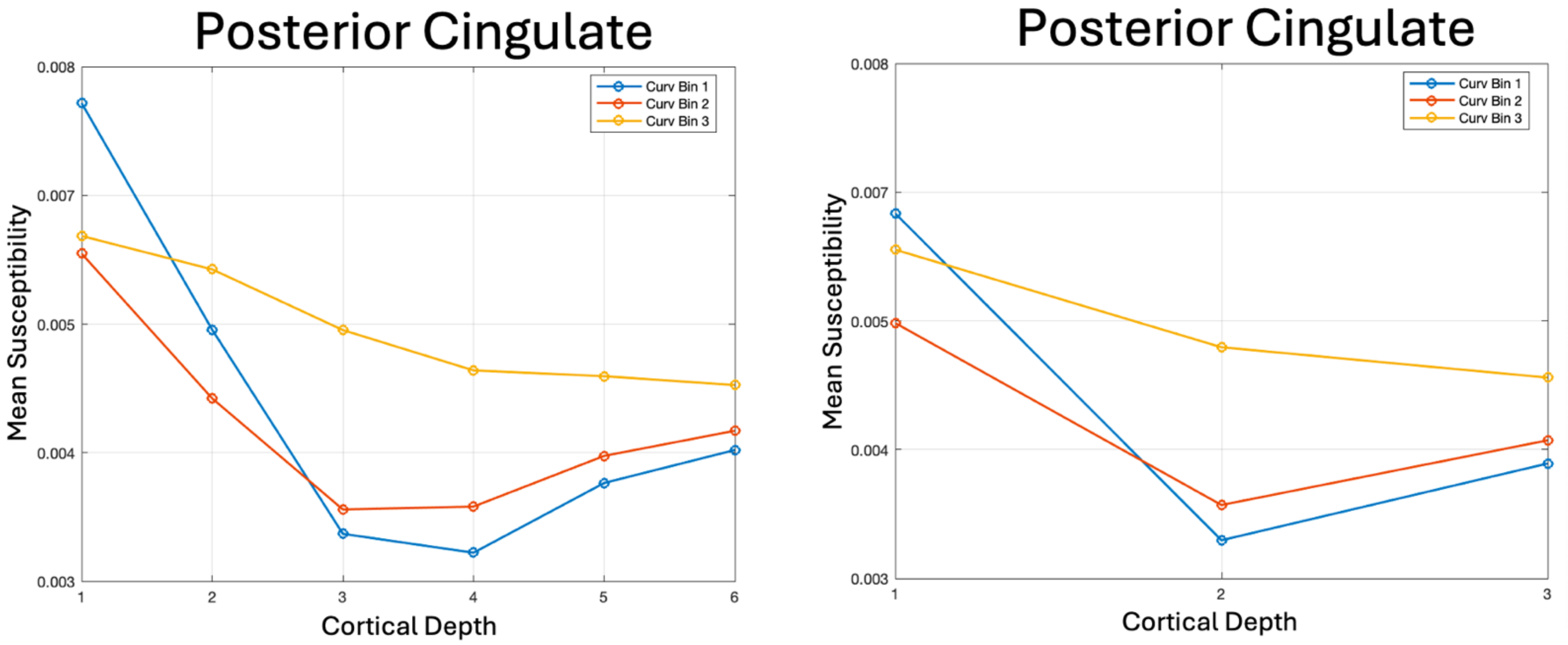

Supplementary Figure 34

Comparative graphs illustrate mean susceptibility values averaged across all participants ($N = 60$) when sampling at six (left) versus three (right) cortical depths for each curvature bin (crown = blue; bank = orange; fundus = yellow) for the isthmus of the cingulate. While sampling at six depths may introduce some redundancy into the model, the overall susceptibility patterns remain similar. Restricting the analysis to three depths appears to risk overlooking subtle yet informative differences that only emerge at finer depth granularity. Susceptibility is measured in parts per million (ppm).

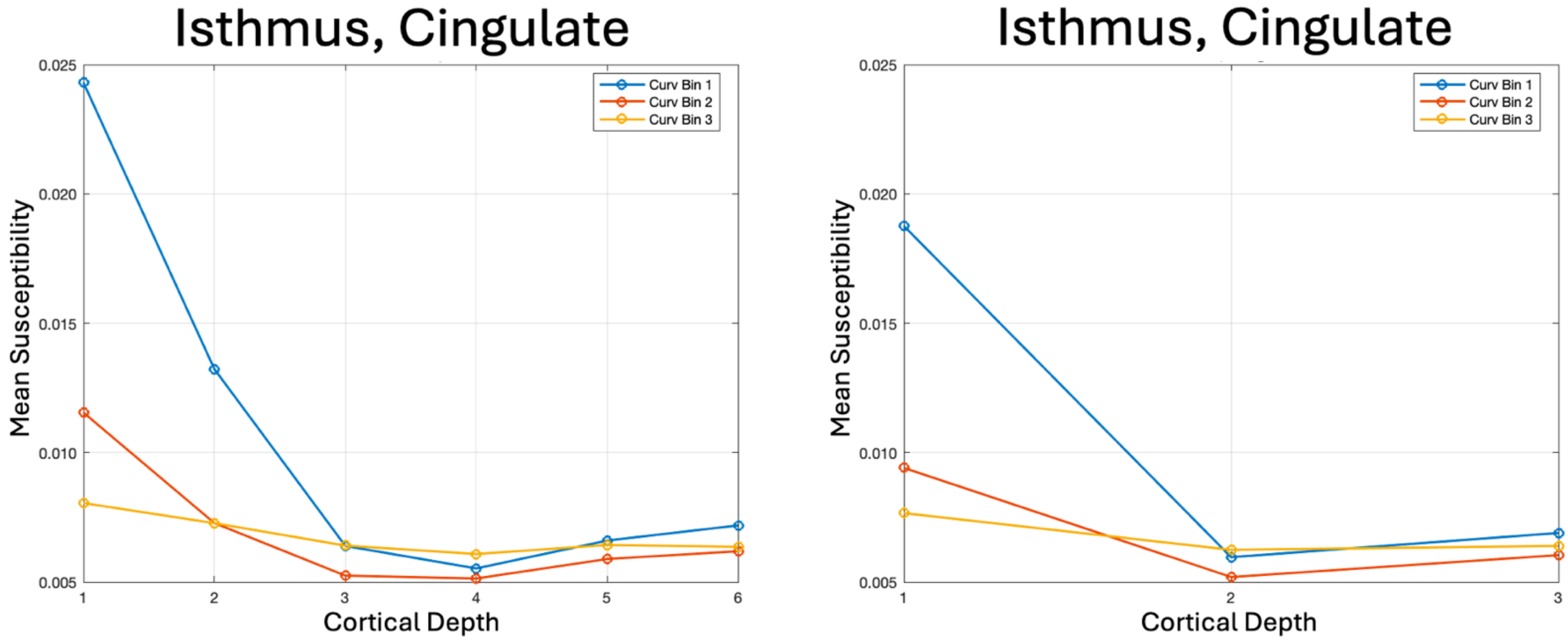

Supplement: fcaf110_Supplementary_Data [file fcaf110_supplementary_data.zip › Supplementary Materials.pdf]
